# Fusion Molecules Between the STAT5b Inhibitor Stafib‐2‐CR and a Cereblon Ligand

**DOI:** 10.1002/open.202600007

**Published:** 2026-04-20

**Authors:** Theresa Münzel, Karl Christian Seidenstücker, Christoph Protzel, Angela Berg, Thorsten Berg

**Affiliations:** ^1^ Institute of Organic Chemistry Leipzig University Leipzig Germany

**Keywords:** biological activity, inhibitors, protein–protein interactions, transcription factors

## Abstract

Selective inhibition of the transcription factor STAT5b is challenging owing to the high degree of similarity to the family member STAT5a. We recently reported catechol bisphosphates as selective inhibitors of STAT5b, with Stafib‐2‐CR as the most potent selective STAT5b inhibitor reported to date. Here, we describe the design and synthesis of fusion molecules between Stafib‐2‐CR and a ligand of the E3 ligase cereblon in an effort toward STAT5b‐selective proteolysis‐targeting chimeras (PROTACs). The fusion molecules retain their activity against STAT5b in competitive fluorescence polarization assays, and the most potent compound is equally active against STAT5b as Stafib‐2‐CR, indicating that the choice of exit vector and linker is suitable for binding to STAT5b. However, conversion of the most potent fusion molecule into a cell‐permeable prodrug turned out to be difficult, pointing toward the challenges of combining prodrug strategies with PROTAC technology.

## Graphical abstract

The design and synthesis of fusion molecules between the selective STAT5b inhibitor Stafib‐2‐CR and a ligand of the E3 ubiquitin ligase cereblon is reported. Activity analysis in fluorescence polarization assays and isothermal titration calorimetry indicated that the chosen attachment point for the linker is well‐suited to retain activity of the fusion molecules against STAT5b.

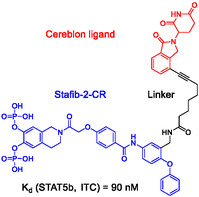

## 1 Introduction

Signal transducer and activator of transcription (STAT) proteins are a family of latent cytoplasmic transcription factors that convey signals from activated cell surface receptors and/or non‐receptor tyrosine kinases to the nucleus [1, 2, 3]. Constitutive activation of the closely related family members STAT5a and STAT5b is observed in numerous human tumor types, including human leukemias expressing the Bcr‐Abl fusion protein, prostate cancer, nasophyryngal carcinoma, and squamous carcinoma of the head and neck (SCCHN) [2]. However, recent data indicate that STAT5b is the main driver of tumorigenesis [4, 5, 6, 7, 8], making selective inhibition of STAT5b an attractive approach for cancer biology.

**FIGURE 1:**
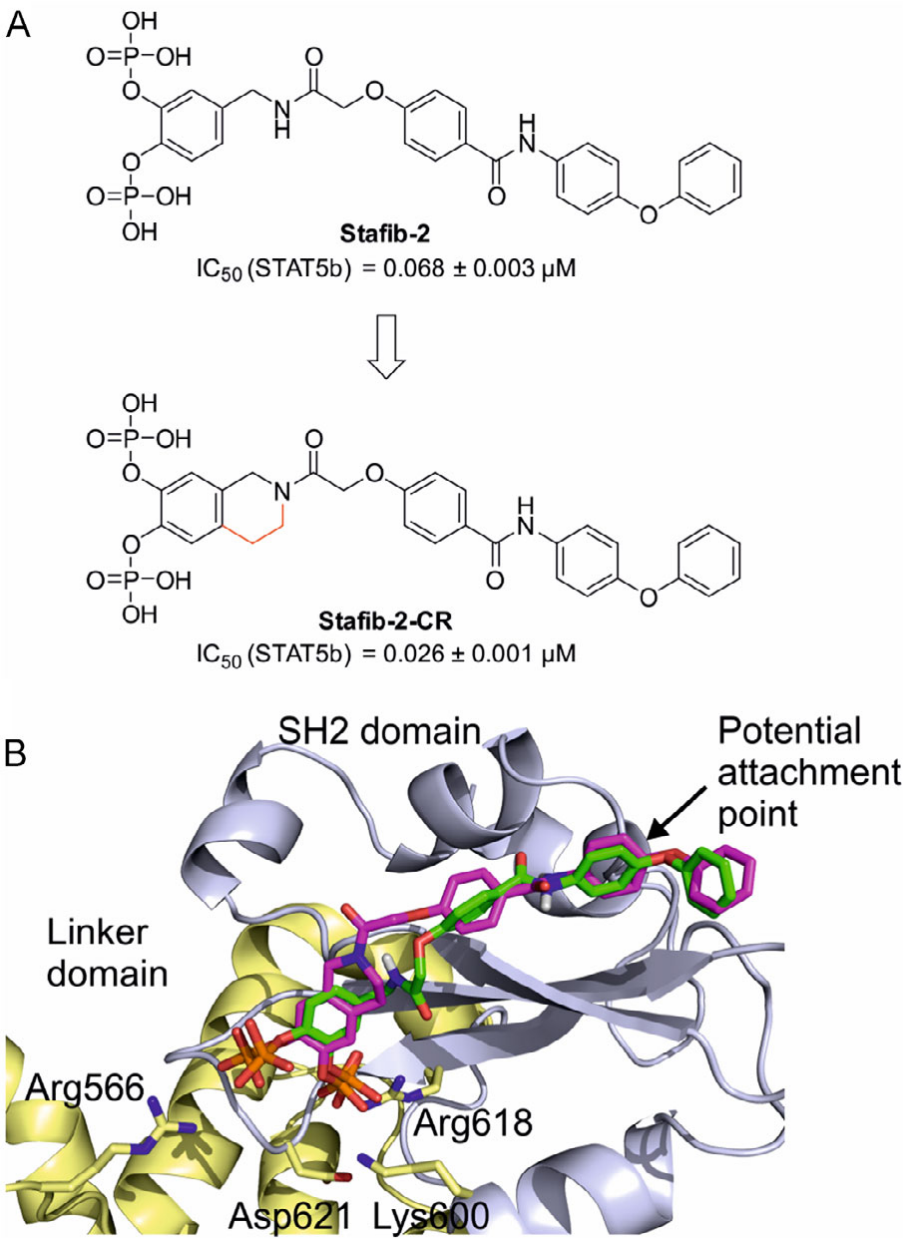
(A) Development of Stafib‐2‐CR from Stafib‐2 [12]. (B) Docking of Stafib‐2‐CR (carbon atoms in magenta) into a previously published homology model of human STAT5b [13] based on mouse STAT5a (PDB:1Y1U) [14] and comparison with the previously proposed binding mode of Stafib‐2 (carbon atoms in green) [15]. The SH2 domain is colored in light blue, and the linker domain in yellow. Amino acids for which side chain flexibility was allowed in the docking process (Arg566, Lys600, Arg618, Asp621) are highlighted. The figure was generated using PyMOL [16].

We recently presented catechol bisphosphates as the first chemical entities that inhibit STAT5b with selectivity over STAT5a [9, 10, 11]. The current most potent published compound is Stafib‐2‐CR [12], which was developed by applying a conformation restriction strategy to the STAT5b inhibitor Stafib‐2 (Figure 1A) [11]. Stafib‐2‐CR inhibits STAT5b in the low nanomolar concentration range (IC_50_ = 0.026 µM) and with ≈70‐fold selectivity over STAT5a [12]. Comparative analysis of wild‐type and point mutant proteins using various catechol bisphosphate inhibitors pointed to Arg566 in the STAT5b linker domain as the molecular reason for the selectivity for STAT5b over STAT5a (Figure 1B) [15]. One of the phosphoryl groups of catechol bisphosphates can engage in electrostatic interactions with the guanidinium side chain of Arg566 in STAT5b, but not with the side chain of Trp566 present in STAT5a in this position. Docking of Stafib‐2‐CR into a previously published homology model of STAT5b [13] using AutoDock FR [17, 18] suggests a very similar binding pose as was proposed for Stafib‐2 [15].

Besides occupancy‐based inhibition by ligands of protein domains, event‐driven inhibition by molecules that induce degradation of their target protein is a powerful way by which to inhibit protein functions [19, 20]. This can be implemented by connecting either a hydrophobic tag or a ligand of a E3 ubiquitin ligase to the protein of interest (POI) via a linker. The latter approach provides proteolysis‐targeting chimeras (PROTACs) [19, 20]. Formation of a ternary complex consisting of the POI, the PROTAC, and the E3 ligase leads to polyubiquitination of the POI, triggering its subsequent degradation via the proteasomal pathway [21]. Most PROTACs recruit the E3 ligases cereblon or von‐Hippel‐Lindau (VHL) via specific ligands of the respective E3 ligases. Recently, PROTACs that target and degrade STAT3 [22, 23, 24, 25] and STAT5a/5b [26, 27] have been published, utilizing a derivative of lenalidomide[22, 23, 24, 26, 27] or thalidomide [25] as cereblon ligands. However, STAT5b‐selective PROTACs have not yet been presented.

## 2 Results and Discussion

**SCHEME 1:**
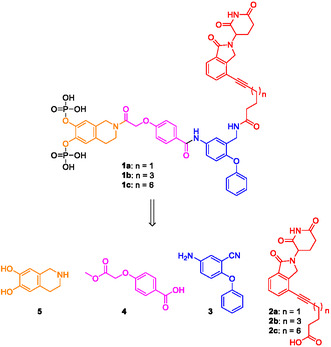
Structures of target compounds **1a**‐**c** and essential building blocks.

Docking of Stafib‐2‐CR into STAT5b suggested the phenoxyphenyl part of the molecule, especially the *meta*‐position relative to the amide function, as an appropriate attachment site for a linker reaching out to a ligand of an E3 ligase (Figure 1B). Our design was inspired by the structure of potent PROTACs against STAT3 [22, 23, 24] and STAT5 [26, 27], in which a STAT SH2 domain ligand was fused to a ligand of the E3 ubiquitin ligase cereblon via an alkynyl linker (Scheme 1). Synthetic access to the target molecules **1a**‐**c** can be achieved via the building blocks **2a**‐**c** comprising a derivative of the cereblon ligand lenalidomide, in which the amino group is replaced by an alkyne linker, the phenoxyphenyl derivative **3** comprising the attachment point for the linker, the *para*‐alkoxybenzoic acid derivative **4,** and the tetrahydroisoquinoline fragment **5** (Scheme 1).

**SCHEME 2:**
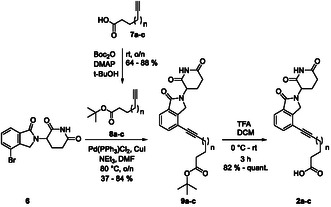
Synthesis of the linker/cereblon ligand building blocks **2a**‐**c**.

The building blocks **2a**‐**c** comprising the cereblon ligand and the linker were generated by Sonogashira coupling of the commercially available bromo derivative of lenalidomide **6** (Scheme 2) with the ω‐alkynyl *tert*‐butyl esters **8a**‐**c** in 37–84% yield. **8a**‐**c** had been generated by *tert*‐butylation of the corresponding ω‐alkynyl carboxylic acids **7a**‐**c** in 64–88% yield. Protection of **7a**‐**c** was essential, as coupling of the free carboxylic acid **7b** with **6** had resulted in an inseparable mixture of products. We decided to use the *tert*‐butyl esters as they can be cleaved under acidic conditions, which was crucial considering the possible hydrolytic cleavage of the glutarimide function under basic conditions [28]. After successful coupling, deprotection of **9a**‐**c** was carried out using trifluoroacetic acid in dichloromethane to provide the acids **2a**‐**c** (82% ‐ quantitative yield).

**SCHEME 3:**
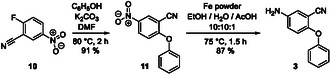
Synthesis of building block **3**.

The phenoxyphenyl derivative **3** comprising the attachment point for the linker on the STAT5b ligand was generated from 2‐fluoro‐5‐nitrobenzonitrile **10** and phenol in 91% yield (Scheme 3) [29]. The resulting nitro compound **11** was reduced to **3** (87% yield). The cyano group of **3** serves as a masked amino group for connecting the STAT5b inhibitor and the cereblon ligands **2a**‐**c** later in the synthesis.

**SCHEME 4:**
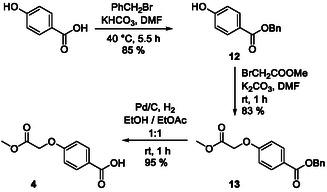
Synthesis of the *para*‐alkoxybenzoic acid building block **4**.

**SCHEME 5:**
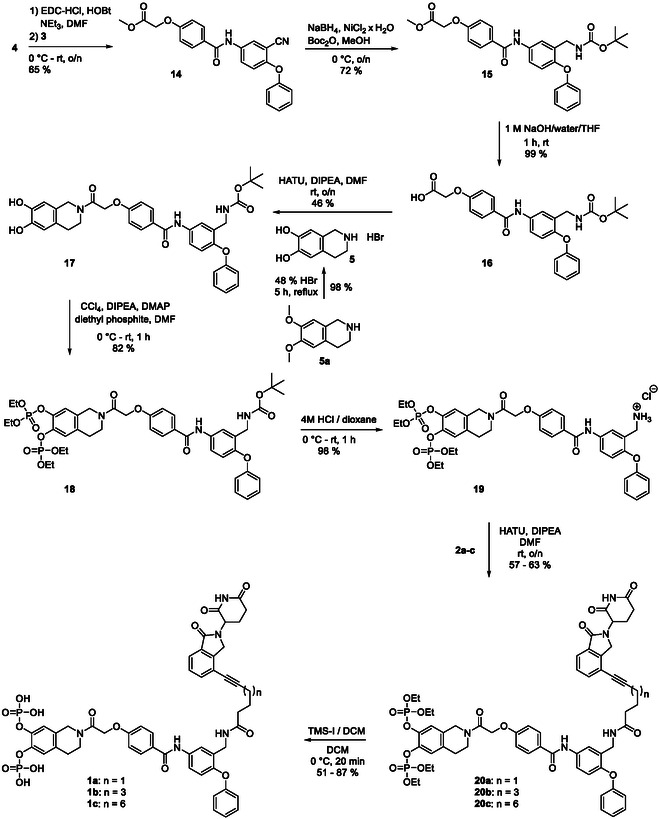
Synthesis of the target molecules **1a**‐**c**.

The *para*‐alkoxybenzoic acid building block **4** was synthesized starting from *para*‐hydroxybenzoic acid, which was converted to the benzyl ester **12** (85% yield) and extended to **13** by reaction with bromomethyl acetate (83% yield, Scheme 4). Hydrogenation of the latter provided **4** (95% yield). This synthesis is similar to our previously published synthesis of Stafib‐2 [11], but employs methyl bromoacetate instead of ethyl bromoacetate, as we otherwise observed a transesterification of the ethyl ester to the corresponding methyl ester in the following nitrile reduction in methanol (see Scheme 5).

Coupling between the amine **3** and the acid **4** provided the amide **14** (65% yield, Scheme 5). Reduction of its nitrile function by *in situ* generated nickel boride, and trapping of the generated amine with di‐*tert*‐butyl dicarbonate [28], provided the Boc‐protected amine **15** (72% yield). Ester hydrolysis under basic conditions gave the acid **16** (99% yield), which was coupled with the tetrahydroisoquinoline building block **5** (generated from the catechol ether **5a**) to provide the amide **17** (46% yield). Atherton–Todd phosphorylation [30] with diethyl phosphite afforded **18** (82% yield), which was converted to the free amine **19** by removal of the *N*‐Boc‐group under water‐free acidic conditions (98% yield). **19** was coupled to the building blocks **2a**‐**c** representing the cereblon ligands, providing the fusion molecules **20a**‐**c** in yields of 57–63%. Trimethylsilyl iodide‐mediated cleavage of the ethyl ester functionalities, followed by purification via RP‐HPLC, provided the target molecules **1a**‐**c** in yields of 51–87%.

**FIGURE 2:**
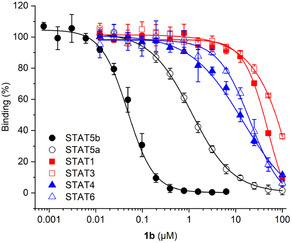
Activity of **1b** in FP assays against STAT proteins. Error bars represent standard deviations (*n* = 3).

Activity analysis of **1a**‐**c** against STAT5b in competitive fluorescence polarization (FP) assays was carried out using a tag‐free STAT5b construct designed for use in co‐crystallization studies and 5‐carboxyfluorescein‐GpYLVLDKW as a fluorescent‐labeled tracer (used at a final concentration of 10 nM) [31]. The linker length present in **1b** (*n* = 3, Scheme 5) was found to be the most suitable (IC_50_ = 0.058 ± 0.009 µM, Table 1, Figure S1A). A shorter linker present in **1a** (*n* = 1, Scheme 5) was tolerated almost as well (IC_50_ = 0.069 ± 0.008 µM, Table 1), but the longer linker (*n* = 6, Scheme 5) present in **1c** was less suited for STAT5b inhibition (IC_50_ = 0.14 ± 0.01 µM, Table 1). The fusion molecules **1a**‐**c** displayed high selectivity for STAT5b over STAT5a (Table 1). Further activity analysis of the most potent fusion molecule **1b** indicated excellent selectivity against other STAT proteins (Figure 2, Table S1). In FP assays using the maltose binding protein (MBP)‐tagged STAT5b construct, which was used in our previous studies,[9, 10, 11, 12, 31] the fusion molecule **1b** (IC_50_ = 0.027 ± 0.002 µM, Figure S1A) was twofold more potent than against the tag‐free STAT5b construct, and thus equally active as previously published for Stafib‐2‐CR against MBP‐tagged STAT5b (IC_50_ = 0.026 ± 0.001 µM) [12], indicating that the chosen attachment point of the linker‐cereblon unit in **1b** was very well tolerated. The twofold higher activity of **1b** against MBP‐tagged STAT5b than against tag‐free STAT5b is consistent with the twofold higher affinity of the fluorescent probe used in the FP assays, 5‐carboxyfluorescein‐GpYLVLDKW, for the MBP‐tagged STAT5b (K_d_ = 0.063 ± 0.003 µM, Figure S1B) as compared to the tag‐free STAT5b construct (K_d_ = 0.119 ± 0.006 µM, Figure S1B).

**TABLE 1:** Structures and activities of STAT5b inhibitors 1a‐c against STAT5a and STAT5b in FP assays using tag‐free STAT5b and the fluorescent tracer 5‐carboxyfluorescein‐GpYLVLDKW.

Compound	Structure	**STAT5b** **IC**_**50**_ **(µM)**	**STAT5a** **IC**_**50**_ **(µM)**	**Selectivity** **IC**_**50**_ **(STAT5a)/IC**_**50**_ **(STAT5b)**
**1a**	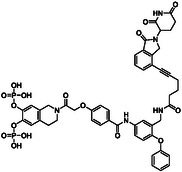	0.069 ± 0.008	1.46 ± 0.08	21
**1b**	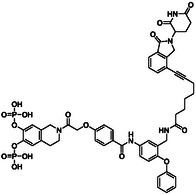	0.058 ± 0.009	1.19 ± 0.16	21
**1c**	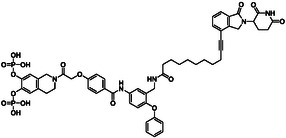	0.14 ± 0.01	5.87 ± 0.67	42

**FIGURE 3:**
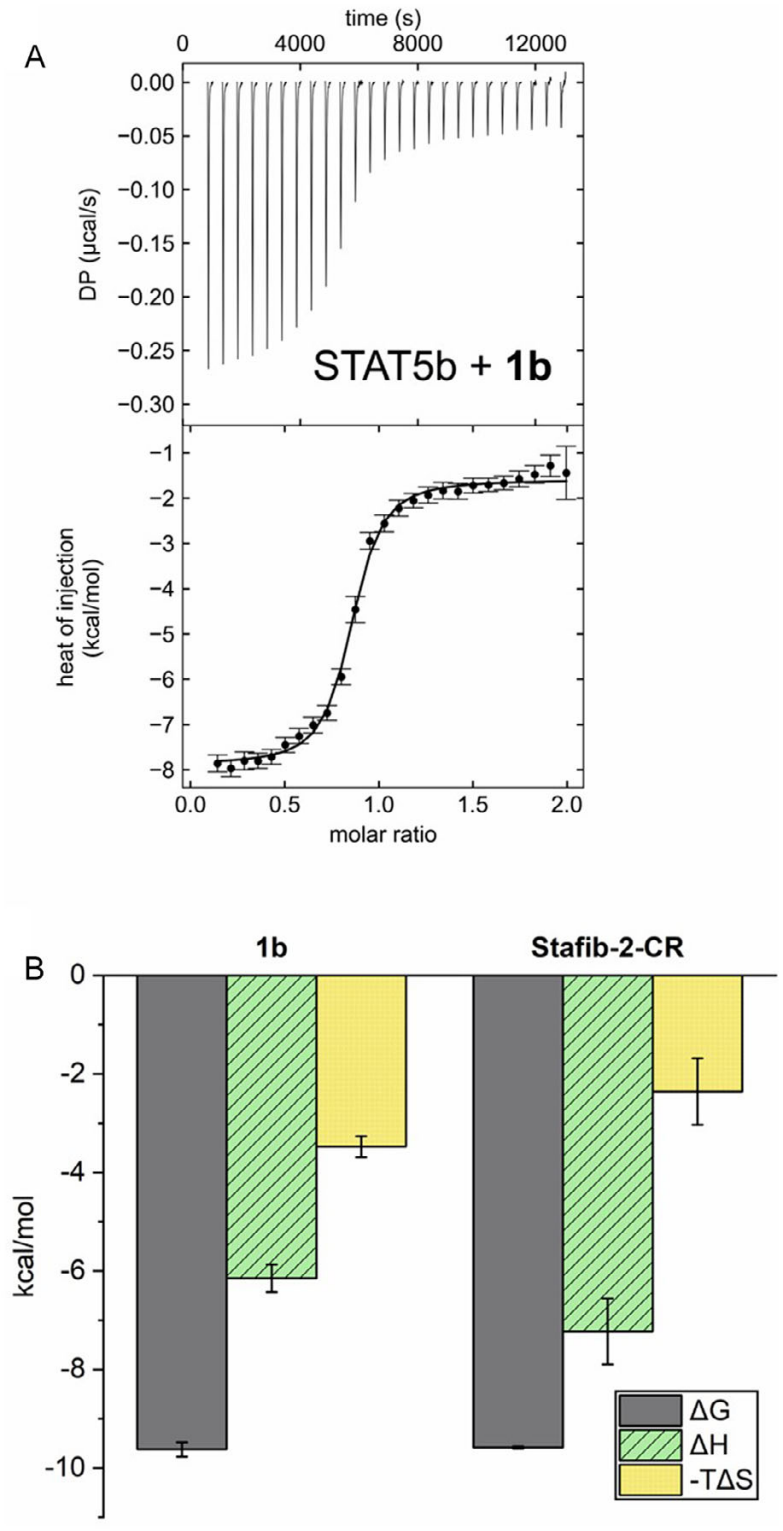
(A) Thermogram of binding between STAT5b and **1b** by ITC. Error bars represent integration errors assigned by the data analysis software NITPIC for the depicted individual experiment [32]. (B) Analysis of thermodynamic parameters for STAT5b binding of **1b** (*n* = 4) and Stafib‐2‐CR (*n* = 3) [12]. Mean values ± standard deviations are given.

In isothermal titration calorimetry (ITC), the dissociation constant for the interaction between **1b** and STAT5b (K_d_ = 0.090 ± 0.022 µM, Figure 3A) was almost identical to that of Stafib‐2‐CR (K_d_ = 0.094 ± 0.003 µM) [12], confirming the results of the FP assays (Figure S1) that the presence of the linker does not interfere with activity against STAT5b. Control titrations of **1b** into buffer did not show any heat release (Figure S2). Despite the virtually identical affinities, the entropic contribution is somewhat larger for **1b** than for Stafib‐2‐CR (Figure 3B) [12], while the enthalpic contribution is correspondingly lower.

**FIGURE 4:**
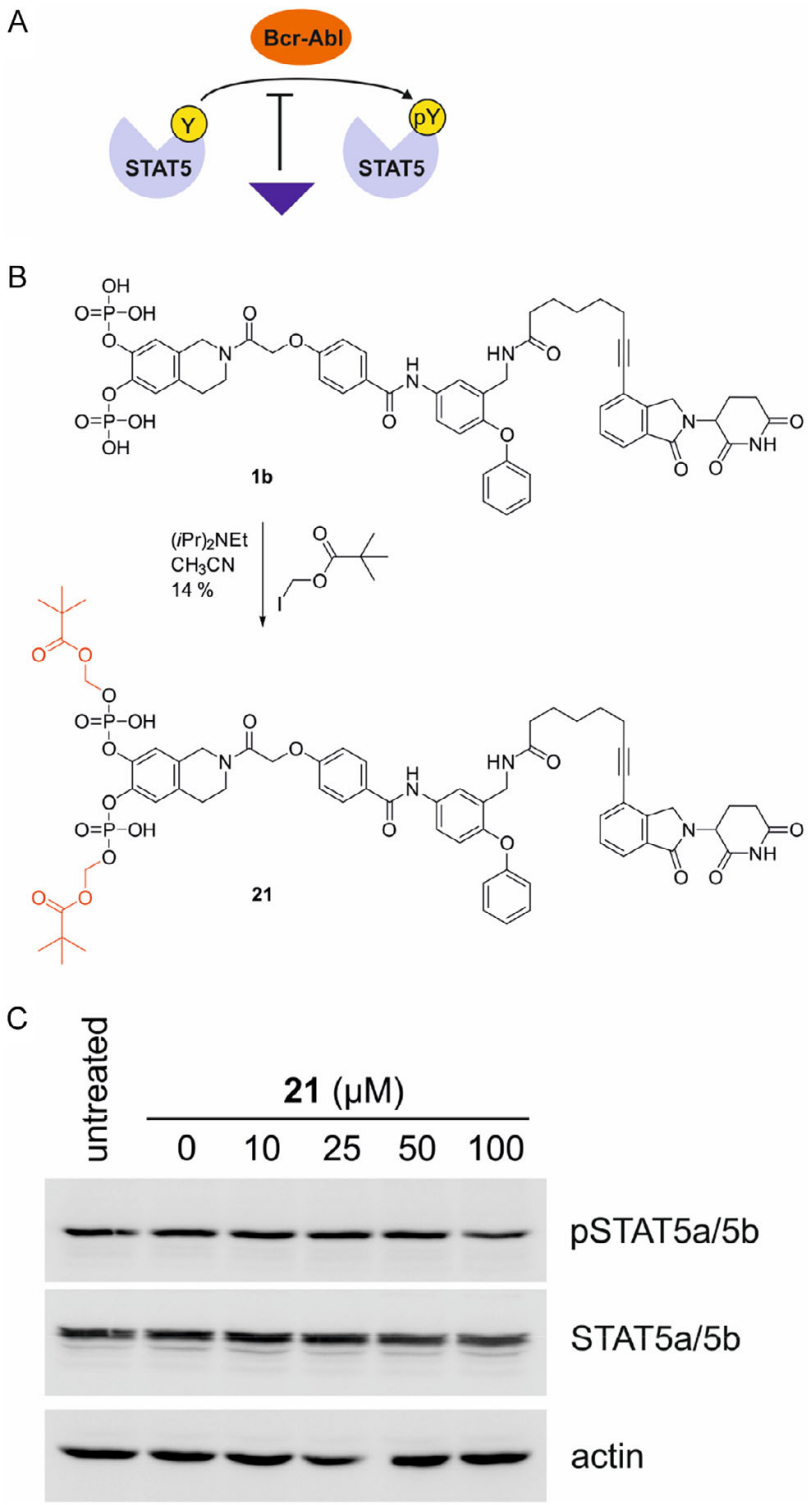
(A) Bcr‐Abl induces tyrosine phosphorylation of STAT5a/5b in K562 cells, which can be blocked by a ligand of the SH2 domain. (B) Synthesis of the diester **21** from **1b**. (C) Western blot analysis of K562 cells treated with **21** for 8 h.

In most cases of chronic myelogenous leukemia (CML), the non‐receptor tyrosine kinase Bcr‐Abl constitutively phosphorylates both STAT5 proteins on a conserved tyrosine residue (STAT5a: Tyr694; STAT5b: Tyr699) located C‐terminal of the SH2 domain [9]. Ligands of the SH2 domain will inhibit this process. Hence, ligand binding to the STAT5 SH2 domains can be analyzed by assessing the tyrosine phosphorylation state of STAT5a/5b using a phosphorylation‐specific antibody (Figure 4A).

A complicating factor when analyzing the activity of catechol bisphosphates on cells is their poor cell permeability caused by the presence of four negative charges at physiological pH. We previously showed that the catechol bisphosphate‐based STAT5b inhibitor Stafib‐2 is only active in cells when the four negative charges are masked as pivaloyloxymethyl esters [11]. Consistently, **1b** has no significant effect on STAT5a/5b phosphorylation or total STAT5a/5b levels (Figure S3) in K562 cells, which are derived from a CML patient. We therefore aimed to convert **1b** into the corresponding tetrapivaloyloxymethyl ester, which can be expected to liberate **1b** inside cells after hydrolysis catalyzed by intracellular esterases [33, 34]. However, these efforts resulted in the formation of only the diester **21** (Figure 4B), which turned out to have little activity against STAT5a/STAT5b phosphorylation (Figure 4C and S4) and protein levels in K562 cells probably owing to poor cell permeability. Since previous catechol bisphophates from our group could be fully converted to tetrapivaloyloxymethyl esters [9, 10, 11, 12], we hypothesize that the presence of the linker and the E3 ligase ligand may cause unfavorable intramolecular folding of **1b**, thereby limiting the accessibility of the phosphate oxygens during the esterification reaction. As an alternative, we converted **1b** into the tetraphenyl ester **S1** (Scheme S1), since phenyl esters of phosphonates have been described as being relatively labile to hydrolysis [35]. However, synthesis of **S1** was hampered by poor and irreproducible yields. **S1** showed a small degree of inhibition on STAT5 phosphorylation, but no apparent reduction of STAT5 protein levels (Figure S5). We did not investigate the effect of **21** or **S1** on STAT5a and STAT5b phosphorylation separately using the previously published transfection assay [9] because of the compounds’ low degree of potency against the phosphorylation of endogenous STAT5a/5b. The apparent lack of an effect on total levels of STAT5 protein by **21** and **S1**, despite a small degree of reduction of STAT5 phosphorylation, suggests that their PROTAC activity is at least weaker than their ability to interfere with tyrosine phosphorylation. Stronger effects on STAT5 phosphorylation, ideally mediated by fully protected pivaloyloxymethyl esters, would be necessary to judge this with more certainty. Based on the current data, it is also conceivable that the attachment point and/or the nature and length of the linkers in compounds **1a**‐**c** are unsuited for creating stable and degradation‐competent ternary complexes with STAT5b and cereblon.

## 3 Conclusion

In summary, we have developed fusion molecules between the most potent selective STAT5b inhibitor published to date, Stafib‐2‐CR, and a ligand of the E3 ubiquitin ligase cereblon, in an effort to generate PROTACs that induce degradation of STAT5b with selectivity over STAT5a. Three linker lengths were explored, all of which were tolerated with respect to STAT5b inhibition in vitro. The most potent compound **1b** binds to STAT5b with the same affinity as the underlying parent compound Stafib‐2‐CR in vitro. However, masking all four negative charges of **1b** by pivaloyloxymethyl esters, an established masking group for phosphates [9, 10, 11, 12] and phosphonates [36, 37, 38, 39] directed against STAT proteins, could not be achieved. The partially masked diester **21**, as well as the tetraphenyl ester **S1**, showed only weak inhibition of STAT5 phosphorylation, and no detectable reduction of STAT5 protein levels in K562 human leukemia cells. Stronger effects on STAT5 phosphorylation would be required before conclusions about the origin of the apparent lack of effect on STAT5 protein levels can be drawn. Of note, the two negative charges of the highly potent phosphonate‐based PROTACs targeting the SH2 domains of STAT3 [22, 23, 24] and STAT5a/5b [26, 27] were not masked in a prodrug. This suggests that the remaining two negative charges of the diester **21** may not necessarily be the sole reason for its poor activity. A low degree of chameleonicity [40], the ability of large molecules such as PROTACs to adapt their conformation to the polarity of the environment and thereby hide polar elements while permeating the lipophilic cell membrane, may be another important factor determining the cellular potencies of **21** and **S1**. Fusion molecules with fully masked negative charges will be required to assess whether the chosen attachment point and/or the nature and length of the linkers in compounds **1a**‐**c** are suited for creating degradation‐competent ternary complexes with STAT5b and cereblon.

## Supporting Information

Additional supporting information can be found online in the Supporting Information section. The authors have cited additional references within the Supporting Information [41, 42, 43, 44, 45, 46, 47].

## Funding

This study was supported by Deutsche Forschungsgemeinschaft (BE 4572/4‐2) and Bundesministerium für Bildung und Forschung (02NUK046C).

## Conflicts of Interest

The authors declare no conflicts of interest.

## Supporting information

Supplementary Material

## Data Availability

The data that support the findings of this study are available in the supplementary material of this article.
