# Supplementary material for: Fusion Molecules Between the STAT5b Inhibitor Stafib‐2‐CR and a Cereblon Ligand
Source: ChemistryOpen. 2026 Apr 20;15(5):e202600007. doi: 10.1002/open.202600007 (PMC13093178; doi:10.1002/open.202600007)
Supplement: Supplementary file 1 — Supplementary Material [file OPEN-15-e202600007-s001.pdf]

**Supporting Information**

# Fusion Molecules between the STAT5b Inhibitor Stafib-2-CR and a Cereblon Ligand

Theresa Münzel,[a] Karl Christian Seidenstücker,[a] Christoph Protzel,[a] Angela Berg,[a] and Thorsten Berg*[a]

## Table of Contents

Table S1 .......... 2

Figure S1 .......... 2

Figure S2 .......... 3

Figure S3 .......... 3

Figure S4 .......... 4

Figure S5 .......... 4

Scheme S1 .......... 5

Plasmids, protein expression and purification .......... 5

Fluorescence polarization assays .......... 6

Isothermal titration calorimetry .......... 6

Cell culture and treatment .......... 7

Western blotting .......... 7

General information on compound synthesis .......... 7

Synthesis and spectroscopic characterization of compounds .......... 8

NMR spectra .......... 38

Supporting references .......... 46

**Table S1:** Structure of **1b** and its activity against STATs in competitive FP assays.

| Structure | STAT1 $IC_{50}$ [μM] | STAT3 $IC_{50}$ [μM] | STAT4 $IC_{50}$ [μM] | STAT5a $IC_{50}$ [μM] | STAT5b $IC_{50}$ [μM] | STAT6 $IC_{50}$ [μM] |
|---|---|---|---|---|---|---|
| 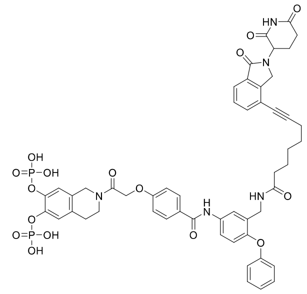 | 40.5 ± 1.3 | 73.1 ± 2.4 | 12.4 ± 2.2 | 1.19 ± 0.16 | 0.058 ± 0.009 | 18.5 ± 2.4 |

## Figure S1

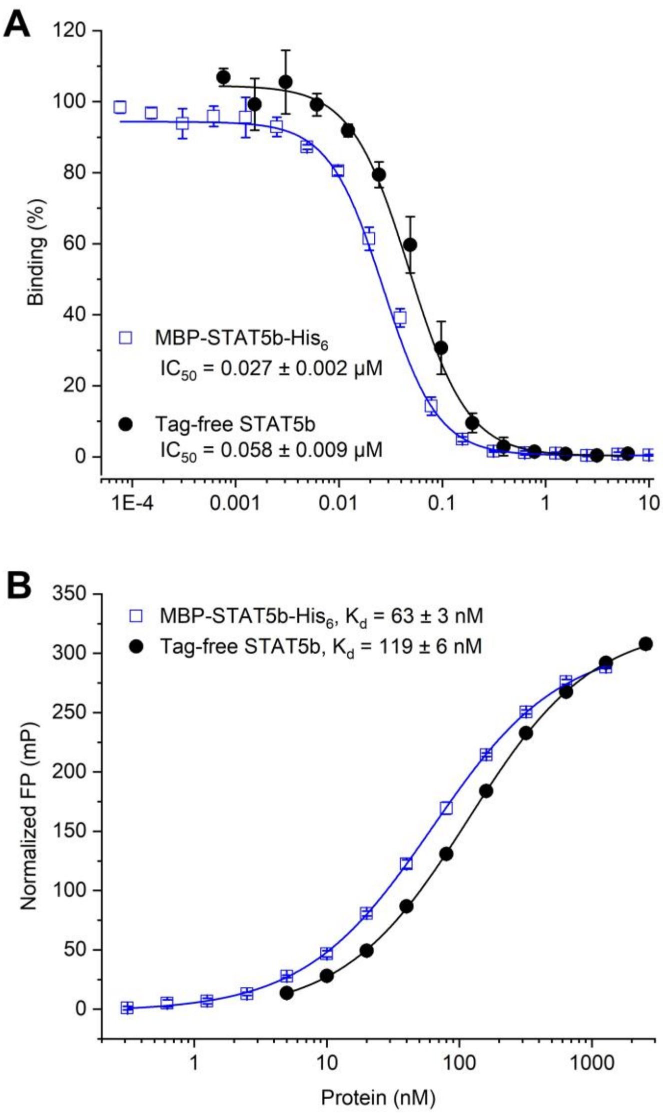


**Figure S1:** A) Activity of **1b** against MBP-STAT5b-$His_6$ and tag-free STAT5b in competitive FP assays. Error bars represent standard deviations (n = 3). B) Affinity of the probe 5-carboxyfluorescein-GpYLVLDKW for MBP-STAT5b-$His_6$ and tag-free STAT5b in FP assays. Error bars represent standard deviations (n = 3).

**Figure S2**

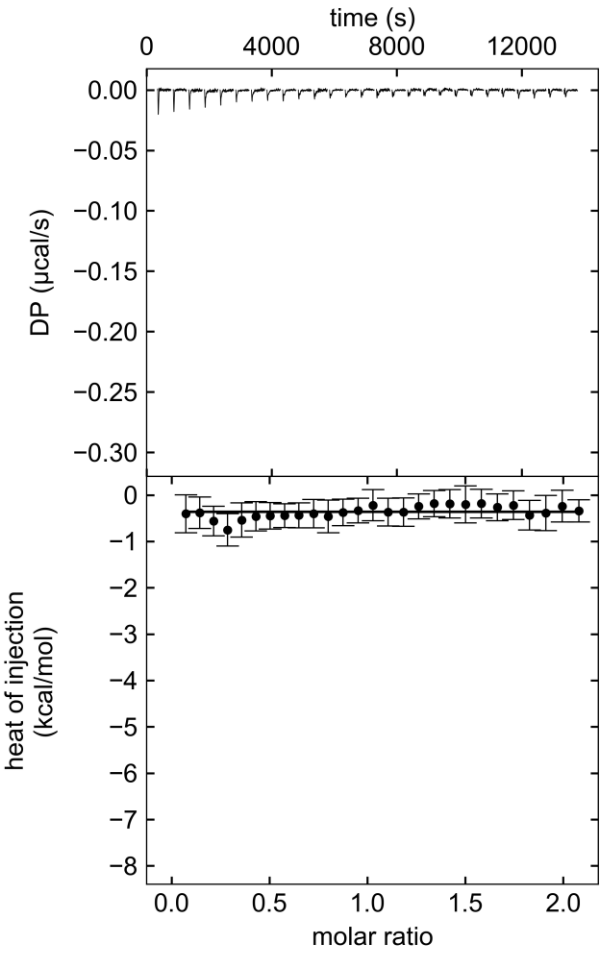


**Figure S2:** Titration of 100 µM **1b** dissolved in ITC buffer to a final DMSO concentration of 2 % (*v*/*v*) into ITC buffer supplemented with 2 % (*v*/*v*) DMSO.

**Figure S3**

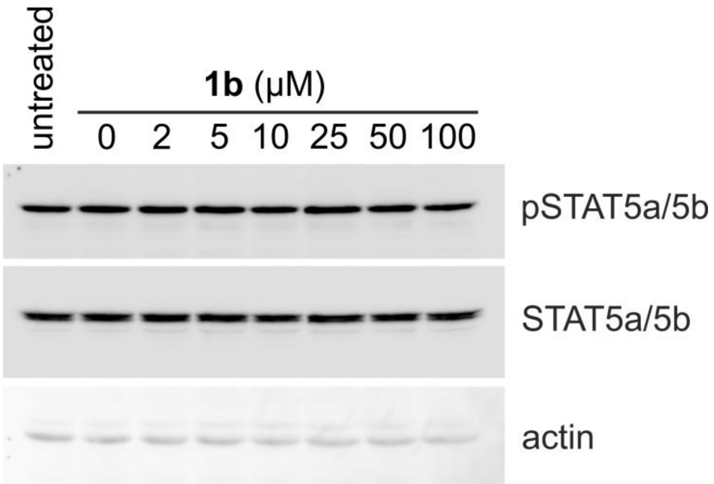


**Figure S3:** Western blot analysis of K562 cells treated with **1b** for 4 h.

**Figure S4**

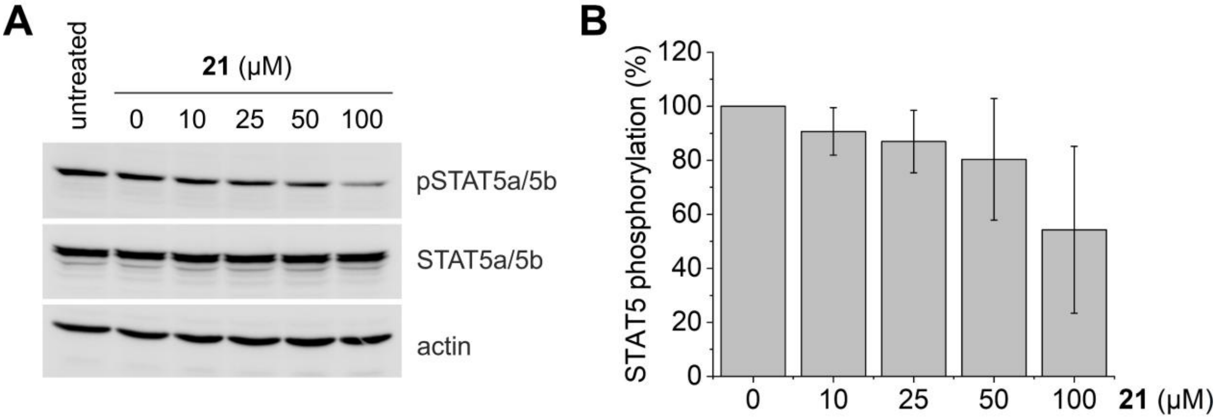


**Figure S4:** A) Repeat experiment of the data shown in Figure 4. Western blot analysis of K562 cells treated with **21** for 8 h. B) Combined quantification of the data shown in Figures 4 and S4A. Mean values and standard deviations are shown (n = 2).

**Figure S5**

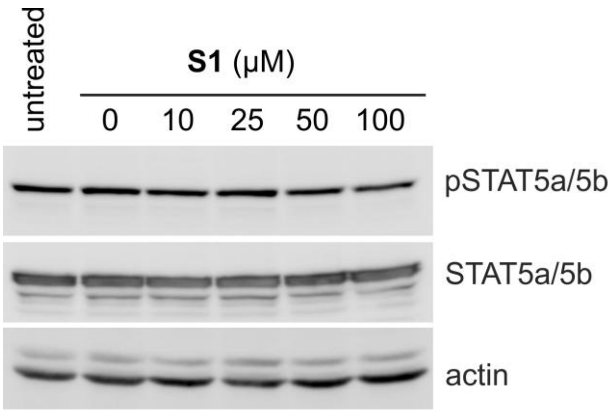


**Figure S5:** Western blot analysis of K562 cells treated with **S1** for 8 h.

## Scheme S1

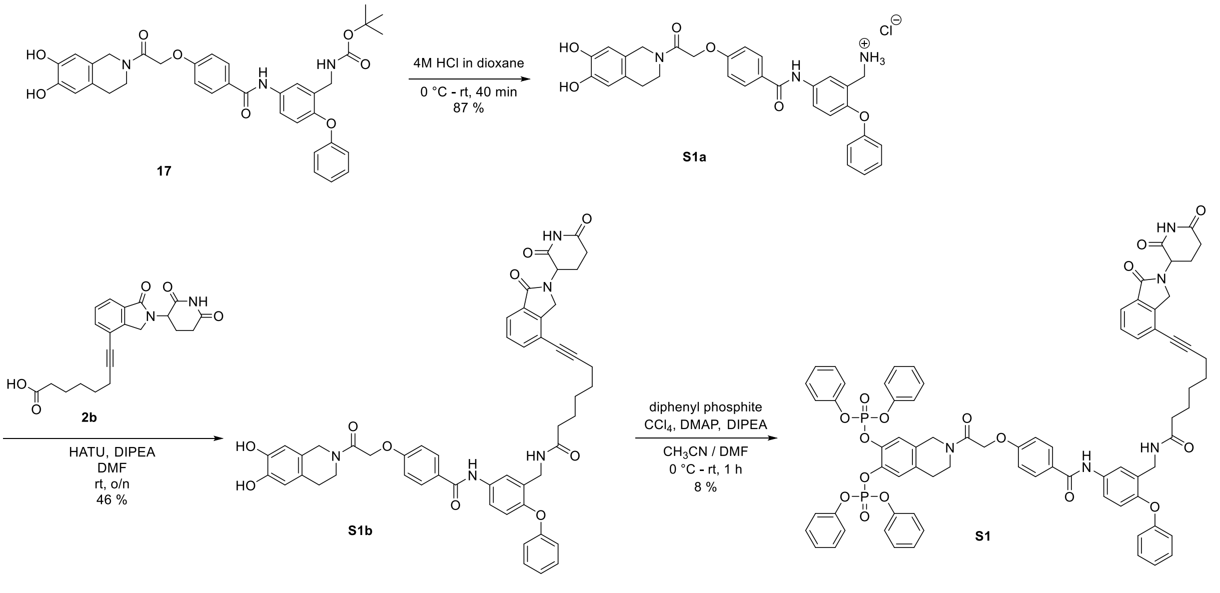


**Scheme S1**: Synthesis of **S1**.

## Plasmids, protein expression and purification

DNA coding for amino acids 128-717 of human STAT5b was amplified by PCR from template DNA and cloned into the BsaI site of a pE-SUMO plasmid (Life Sensors Inc.), resulting in a $His_6$-SUMO-tag modification of STAT5b. $His_6$-SUMO-STAT5b protein was expressed following the protocol previously described.[1] The fusion protein $His_6$-SUMO-STAT5b was first purified via affinity chromatography on His-Bind resin (Millipore), followed by enzymatic removal of the N-terminal $His_6$-SUMO tag with SUMO-Protease (Sigma-Aldrich) at 3 U per 100 µg fusion STAT5b. After digestion at 4°C over night, the protein sample was purified via affinity chromatography on His-Bind resin, which retains the $His_6$-SUMO tag. Subsequently, tag-free STAT5b was dialyzed against 20 mM HEPES (pH 7.0), 200 mM NaCl and 5 mM DTT, and was purified via size exclusion chromatography using a HiLoad 16/600 Superdex 200 pg (Cytiva) column on an Äkta Explorer. Tag-free STAT5b protein was snap frozen in liquid nitrogen and was used for fluoresence polarization assays with compounds **1a-c**, the results of which are shown in Table 1.

Expression constructs encoding STAT1,[2] STAT3,[3] MBP-STAT4,[4] MBP-STAT5a,[5] MBP-STAT5b[6] and STAT6[4] have been described (MBP: maltose binding protein). Protein expression and purification have also been described.[1, 7] His-tagged STAT1, STAT3, MBP-STAT4, MBP-STAT5a, MBP-STAT5b, and STAT6 proteins used for fluorescence polarization assays were purified twice by affinity chromatography on His-Bind resin (Millipore). After dialysis against a buffer containing 100 mM NaCl, 50 mM HEPES pH 7.5, 1mM EDTA, 1mM DTT, 10% (*v*/*v*) glycerol and 0.1% (*v*/*v*) NP-40, they were snap-frozen in liquid nitrogen. STAT5b used for ITC was purified over a single His-Bind resin column, and was dialyzed

against a buffer containing 10 mM Tris pH 8.0 and 50 mM NaCl using dialysis tubing with a 50 kDa cut-off, as previously described.[7] Protein samples used for ITC were not frozen prior to measurement.

**Fluorescence polarization assays**

The ability of a compound to displace a fluorescent-labelled peptide from the SH2-domain of the respective STAT protein was analyzed by competitive fluorescence polarization assays. The following peptide sequences were used: 5-carboxyfluorescein-GY($PO_3H_2$)DKPHVL for STAT1, 5-carboxyfluorescein-GY($PO_3H_2$)LPQTV-$NH_2$ for STAT3, 5-carboxyfluorescein-GY($PO_3H_2$)LPQNID for STAT4, 5-carboxyfluorescein-GY($PO_3H_2$)LVLDKW for STAT5a/b and 5-carboxyfluorescein-GY($PO_3H_2$)VPWQDLI for STAT6 with a final peptide concentration of 10 nM in the assay. The final protein concentration used represented the approximate $K_d$-value of the respective protein batches determined in a fluorescence binding assay: 83 nM for STAT1, 81 nM for STAT3, 32 nM for STAT4, 177 nM for STAT5a, 62 nM for MBP-STAT5b-$His_6$, 117 nM for tag-free STAT5b, 85 nM for STAT6. The assays were carried out in FP-assay buffer, which consisted of 10 mM Tris (pH 8.0), 50 mM NaCl, 1 mM DTT, 1 mM EDTA, 0.1 % Nonidet P-40 substitute and 2 % DMSO. A dilution series of the test compounds in water or DMSO was incubated with the respective protein for 60 min at room temperature, followed by the addition of fluorescent-labelled peptide. A negative control consistent of 10 nM peptide and 2 % water or DMSO and a positive control consistent of protein, 10 nM peptide and 2 % water or DMSO were further prepared. Samples were transferred to 384-well microtiter plates and fluorescence polarization was measured after 60 min in triplicates. An excitation wavelength of 485 nm was used to detect the emission at 535 nm. $IC_{50}$ values were determined by plotting the amount of peptide-bound protein against the concentration of the test compound. A logarithmic curve fit in Origin 2017 software was used to calculate percent binding.

**Isothermal titration calorimetry**

Experiments were carried out using a VP-ITC Micro Calorimeter (MicroCal) as previously described.[7] **1b** was dissolved in DMSO as a 5 mM stock and was diluted 1/50 in ITC buffer (final ligand concentration: 100 µM). In order to avoid errors caused by dilution heat, 2 % (*v*/*v*) DMSO was added to the buffer containing the protein. Proteins were degassed before the experiments using a ThermoVac sample degassing station. ITC experiments were carried out using the following parameters: 10 µM STAT5b, 100 µM **1b**, 28 injections with one 0.5 µL preinjection and 27 times 10 µL single injection volume, 150 s initial delay, 500 s spacing between injections, 20 µcal/s reference power, 220 rpm stirring at 25 °C. A low-noise integration approach was used for data analysis with NITPIC[8-9] and SEDPHAT.[10] A one-site

binding model was used for data fitting. Figures were generated using GUSSI.[11] The experiment was carried out in quadruplicate.

**Cell culture and treatment**

K562 cells were cultured in RPMI 1640 medium containing 10% FBS, 2 mM L-glutamine and penicillin/streptomycin as previously described.[7] Cells were treated with test compound or DMSO for the indicated incubation periods (4 h for **1b**, 8 h for **21** and **S1**) at a final DMSO concentration of 0.2%. Subsequently, cells were harvested by washing with ice-cold TBS (twice), followed by resuspension in TBS which was supplemented with protease/phosphatase inhibitors (100 ng/ml aprotinin, 1 mM $Na_3VO_4$, 10 mM NaF, 1 mM PMSF). Cells were lysed by applying three freeze-thaw cycles with liquid nitrogen. Lysates were cleared by centrifugation at 20,000 g at 4 °C for 25 minutes.

**Western blotting**

The components of cell lysates were separated by SDS-PAGE (10 % gel), transferred to nitrocellulose membrane (Bio-Rad) and detected using monoclonal rabbit primary antibodies (Cell Signaling), $\alpha$-rabbit-HRP secondary antibody (Dako) and Pierce ECL Plus chemiluminescence reagent (Thermo Scientific) as previously described (reference). Bands were visualized using an ImageQuant system (GE Healthcare) and quantitated using ImageJ software (NIH).[12]

**General information on compound synthesis**

The final products were purified by reversed phase (RP)-HPLC using a 1260 Infinity II from Agilent Technologies with a ReproSil Gold 120 C18 (250 mm x 10 mm; 5 µm particle size) column. Eluent A (0.1 % TFA in water) and eluent B (0.1 % TFA in $CH_3CN$) with a flow rate of 5 mL/min were used.

$^{1}$H, $^{13}$C and $^{31}$P-NMR spectra were recorded on Varian MERCURYplus 300, Varian MERCURYplus 400, Bruker AVANCE III HD 400 and Bruker Fourier 300 spectrometers. Chemical shifts (δ) are reported in parts per million (ppm), referenced to the deuteurated solvents as specified. Assignments were made based on HSQC, HMBC and COSY 2D-NMR spectra. The temperature dependend pseudo-rotation of the amide bonds of the presented compound class partly leads to a second set of signals due to the presence of two rotamers in a varying ratio. Signals are therefore either broadened or doubled showing different signal intensities. Rotamers were defined as R1 for major and R2 for minor form. In certain cases, spectra were recorded under temperature increase or decrease as noted for the concerned compound, resulting in one signal or distingushable signals for R1 and R2. If peak broadening

led to a complete disappearance of the respective signal, 2D NMR experiments were consulted.

High resolution mass spectra were recorded on an Impact II Bruker Daltonics and a MicrOTOF Bruker Daltonics spectrometer using electrospray ionisation. Infrared spectra were recorded on a JASCO FT/IR-4100 fourier transform spectrometer and UV/Vis spectra were recorded on a JASCO V-630 spectrophotometer. Melting points were measured using a Büchi melting point M-560 apparatus.

## Synthesis and spectroscopic characterization of compounds

### *Tert*-butyl hex-5-ynoate (8a)

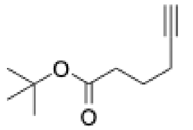

Synthesis of **8a** was carried out essentially as described in the literature.[13] In brief, to a solution of hex-5-ynoic acid **7a** (400 µL, 3.57 mmol, 1.0 eq.) in *tert*-butyl alcohol (2 mL) were added di-*tert*-butyl dicarbonate (1.22 mL, 5.35 mmol, 1.5 eq.) and DMAP (130 mg, 1.07 mmol, 0.3 eq.). After stirring overnight at room temperature, the solvent was removed *in vacuo* and the crude product was purified by column chromatography (3 % EtOAc in hexane, *v/v*) to yield **8a** as a colorless oil (382 mg, 64 %).

**$R_f$** = 0.57 (5 % EtOAc in hexane, *v/v*).
**$^1$H-NMR** (400 MHz, $CDCl_3$): δ = 2.35 (t, *J* = 7.4 Hz, 2H), 2.24 (td, *J* = 7.0, 2.7 Hz, 2H), 1.95 (t, *J* = 2.7 Hz, 1H), 1.84 – 1.76 (m, 2H), 1.44 (s, 9H) ppm.
**$^{13}$C-NMR** (75 MHz, $CDCl_3$): δ = 172.6, 83.7, 80.4, 69.0, 34.4, 28.3, 24.0, 18.0 ppm.
**HRMS** (ESI, pos): *m/z* $[M+H]^+$ calculated for $[C_{10}H_{17}O_2]^+$: 169.1223, found: 169.1229.
**IR** (film): $\tilde{\nu}$ = 3308 (m), 2980 (s), 2935 (w), 1731 (s), 1456 (w), 1368 (s), 1320 (w), 1291 (w), 1254 (m), 1148 (s), 1042 (w), 915 (m), 843 (m), 735 (s), 637 (m) $cm^{-1}$.
**UV / Vis** (DCM): $\lambda_{max}$ = 228 nm.

### *Tert*-butyl oct-7-ynoate (8b)

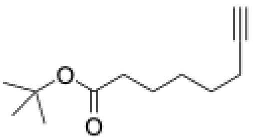

Synthesis of **8b** was carried out essentially as described in the literature.[13] In brief, to a solution of oct-7-ynoic acid **7b** (476 µL, 3.57 mmol, 1.0 eq.) in *tert*-butyl alcohol (10 mL) were added di-*tert*-butyl dicarbonate (1.23 mL, 5.36 mmol, 1.5 eq.) and DMAP (130 mg, 1.07 mmol, 0.3 eq.). After stirring overnight at room temperature, the solvent was removed *in vacuo*. Water (50 mL) was added to the residue, which was extracted with EtOAc (3 x 50 mL). The combined organic phases were washed with brine (1 x 80 mL), dried over $Na_2SO_4$, filtered and carefully concentrated *in vacuo*. The crude product was purified by column chromatography (2 % EtOAc in hexane, *v*/*v*) to yield **8b** as a colorless oil (617 mg, 88 %).

**$R_f$** = 0.40 (2 % EtOAc in hexane, *v/v*).
**$^1$H-NMR** (400 MHz, $CDCl_3$): δ = 2.24 – 2.16 (m, 4H), 1.93 (t, *J* = 2.6 Hz, 1H), 1.65 – 1.49 (m, 4H), 1.44 (s, 11H) ppm.
**$^{13}$C-NMR** (101 MHz, $CDCl_3$): δ = 173.2, 84.6, 80.2, 68.4, 35.6, 28.30, 28.26, 24.7, 18.4 ppm.
**HRMS** (ESI, pos): *m/z* $[M+Na]^+$ calculated for $[C_{12}H_{20}NaO_2]^+$: 219.1356, found: 219.1354.
**IR** (KBr): $\tilde{v}$ = 3418 (br), 2961 (w), 2924 (w), 1638 (m), 1616 (m), 1507 (w), 1488 (w), 1259 (m), 1219 (w), 1023 (w), 802 (w) $cm^{-1}$.
**UV / Vis** ($CHCl_3$): $\lambda_{max}$ = 277 nm.

***Tert*-butyl undec-10-ynoate (8c)**

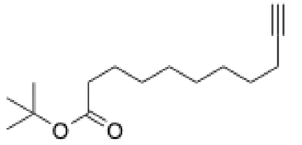

Synthesis of **8c** was carried out essentially as described in the literature.[13] In brief, to a solution of undec-10-ynoic acid **7c** (400 mg, 2.19 mmol, 1.0 eq.) in *tert*-butyl alcohol (2 mL) were added di-*tert*-butyl dicarbonate (755 µL, 3.29 mmol, 1.5 eq.) and DMAP (80 mg, 0.66 mmol, 0.3 eq.). After stirring overnight at room temperature, the solvent was removed *in vacuo* and the crude product was purified by column chromatography (3 % EtOAc in hexane, *v/v*) to yield **8c** as a colorless oil (356 mg, 68 %).

**$R_f$** = 0.43 (5 % EtOAc in hexane, *v/v*).
**$^1$H-NMR** (300 MHz, $CDCl_3$): δ = 2.23 – 2.14 (m, 4H), 1.93 (t, *J* = 2.6 Hz, 1H), 1.61 – 1.48 (m, 4H), 1.44 (s, 9H), 1.42 – 1.34 (m, 2H), 1.34 – 1.25 (m, 6H) ppm.
**$^{13}$C-NMR** (75 MHz, $CDCl_3$): δ = 173.4, 84.9, 80.1, 68.2, 35.7, 29.3, 29.2, 29.1, 28.8, 28.6, 28.3, 25.2, 18.5 ppm.
**HRMS** (ESI, pos): m/z $[M+H]^+$ calculated for $[C_{15}H_{27}O_2]^+$: 239.2006, found: 239.2014.

**IR** (film): ṽ = 3311 (m), 2979 (m), 2931 (s), 2857 (m), 1732 (s), 1458 (w), 1384 (m), 1367 (s), 1292 (w), 1253 (w), 1153 (s), 848 (w), 629 (w) $cm^{-1}$.
**UV / Vis** (DCM): $\lambda_{max}$ = 229 nm.

***Tert*-butyl 6-(2-(2,6-dioxopiperidin-3-yl)-1-oxoisoindolin-4-yl)hex-5-ynoate (9a)**

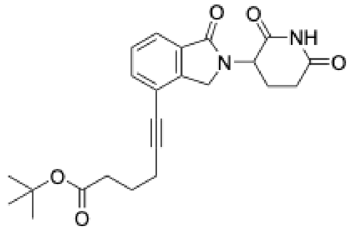


To a solution of 3-(4-bromo-1-oxoisoindolin-2-yl)piperidine-2,6-dione **6** (96 mg, 0.30 mmol, 1.0 eq.) in degassed DMF (1 mL) were added CuI (11 mg, 0.060 mmol, 0.2 eq.), $Pd(PPh_3)_2Cl_2$ (21 mg, 0.030 mmol, 0.1 eq.), degassed $NEt_3$ (1 mL, 7 mmol, 24 eq.) and **8a** (100 mg, 0.594 mmol, 2.0 eq.). The reaction mixture was stirred at 80 °C overnight, followed by the removal of all volatile components under reduced pressure. The crude product was purified by column chromatography (30 % acetone in hexane, *v*/*v*) to yield **9a** as an off-white solid (85 mg, 70 %).

$\mathbf{R_f}$ = (0.29, 3:2 hexane / acetone, *v/v*).
**$^1$H-NMR** (400 MHz, $CDCl_3$): δ = 8.13 (s, 1H, N**H**), 7.80 (dd, *J* = 7.6, 1.1 Hz, 1H), 7.57 (dd, *J* = 7.7, 1.1 Hz, 1H), 7.46 – 7.41 (m, 1H), 5.24 (dd, *J* = 13.3, 5.1 Hz, 1H), 4.49 (d, *J* = 16.7 Hz, 1H), 4.35 (d, *J* = 16.7 Hz, 1H), 2.97 – 2.79 (m, 2H), 2.51 (t, *J* = 7.2 Hz, 2H), 2.46 – 2.37 (m, 3H), 2.26 – 2.18 (m, 1H), 1.95 – 1.86 (m, 2H), 1.45 (s, 9H) ppm.
**$^{13}$C-NMR** (101 MHz, $CDCl_3$): δ = 172.5, 171.1, 169.5, 169.2, 143.7, 134.8, 131.7, 128.5, 123.5, 119.5, 95.1, 80.6, 77.1, 52.0, 47.1, 34.6, 31.7, 28.3, 24.2, 23.6, 19.1 ppm.
**HRMS** (ESI, pos): *m/z* $[M+H]^+$ calculated for $[C_{23}H_{27}N_2O_5]^+$: 411.1914, found: 411.1916.
**IR** (KBr): ṽ = 3422 (s), 3086 (w), 2979 (w), 1707 (s), 1670 (m), 1617 (w), 1483 (w), 1455 (m), 1416 (w), 1369 (m), 1332 (w), 1234 (m), 1209 (w), 1147 (m), 994 (w), 754 (m), 620 (w), 543 (w) $cm^{-1}$.
**UV / Vis** (DCM): $\lambda_{max}$ = 288, 228 nm.
**mp**: 210 °C.

***Tert*-butyl 8-(2-(2,6-dioxopiperidin-3-yl)-1-oxoisoindolin-4-yl)oct-7-ynoate (9b)**

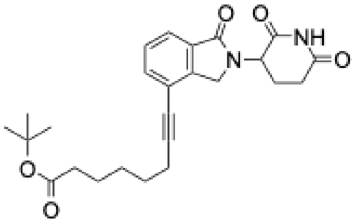

To a solution of 3-(4-bromo-1-oxoisoindolin-2-yl)piperidine-2,6-dione **6** (598 mg, 1.85 mmol, 1.0 eq.) in degassed DMF (6 mL) were added CuI (78 mg, 0.41 mmol, 0.2 eq.), $Pd(PPh_3)_2Cl_2$ (143 mg, 0.204 mmol, 0.1 eq.), degassed $NEt_3$ (5.0 mL, 36 mmol, 19 eq.) and **8b** (400 mg, 2.04 mmol, 1.1 eq.). The reaction mixture was stirred at 80 °C overnight. Upon completion of the reaction, 5 % LiCl-solution was added, followed by an extraction with EtOAc (3 x). The combined organic phases were washed with brine (1 x), dried over $Na_2SO_4$, filtered and concentrated *in vacuo*. The crude product was purified by column chromatography (30 % acetone in hexane, *v/v*) to yield **9b** as a colorless solid (302 mg, 37 %).

$\mathbf{R_f}$ = 0.44 (40 % acetone in hexane, *v/v*).
**$^1$H-NMR** (400 MHz, $CDCl_3$): δ = 8.10 (s, 1H, N**H**), 7.79 (dd, *J* = 7.6, 1.1 Hz, 1H), 7.56 (dd, *J* = 7.6, 1.1 Hz, 1H), 7.43 (t, *J* = 7.6 Hz, 1H), 5.24 (dd, $^3J_{H\text{-}H}$ = 13.3, 5.1 Hz, 1H), 4.49 (d, $^2J_{H\text{-}H}$ = 16.7 Hz, 1H), 4.35 (d, $^2J_{H\text{-}H}$ = 16.7 Hz, 1H), 2.98 – 2.79 (m, 2H), 2.50 – 2.37 (m, 3H), 2.27 – 2.18 (m, 3H), 1.69 – 1.58 (m, 4H), 1.54 – 1.45 (m, 2H), 1.43 (s, 9H) ppm.
**$^{13}$C-NMR** (76 MHz, $CDCl_3$): δ = 173.2, 171.1, 169.6, 169.2, 143.7, 134.8, 131.6, 128.5, 123.4, 119.7, 96.1, 80.3, 77.4, 52.0, 47.1, 35.6, 31.7, 28.5, 28.3, 24.7, 23.6, 19.5 ppm.
**HRMS** (ESI, pos): *m/z* $[M+Na]^+$ calculated for $[C_{25}H_{30}N_2NaO_5]^+$: 461.2047, found: 461.2061.
**IR** (KBr): ṽ = 3416 (br), 2931 (w), 2858 (w), 2227 (w), 1727 (s), 1705 (s), 1670 (s), 1637 (m), 1617 (m), 1483 (w), 1455 (w), 1433 (w), 1368 (m), 1332 (w), 1234 (m), 1208 (m), 1169 (m), 754 (w) $cm^{-1}$.
**UV / Vis** ($CHCl_3$): $\lambda_{max}$ = 299, 290, 258, 250 nm.
**mp**: 220°C (decomposition).

***Tert*-butyl 11-(2-(2,6-dioxopiperidin-3-yl)-1-oxoisoindolin-4-yl)undec-10-ynoate (9c)**

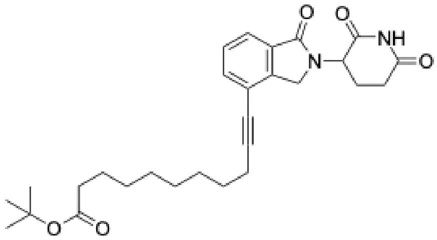

To a solution of 3-(4-bromo-1-oxoisoindolin-2-yl)piperidine-2,6-dione **6** (95 mg, 0.29 mmol, 1.0 eq.) in degassed DMF (1 mL) were added CuI (11 mg, 0.058 mmol, 0.2 eq.), $Pd(PPh_3)_2Cl_2$ (21 mg, 0.030 mmol, 0.1 eq.), degassed $NEt_3$ (1 mL, 7 mmol, 24 eq.) and **8c** (140 mg, 0.587 mmol, 2.0 eq.). The reaction mixture was stirred at 80 °C overnight, followed by the removal of all volatile components under reduced pressure. The crude product was purified by column chromatography (30 % acetone in hexane, *v/v*) to yield **9c** as an off-white solid (119 mg, 84 %).

**$R_f$** = 0.38 (3:2 hexane / acetone, *v/v*).
**$^1$H-NMR** (400 MHz, $CDCl_3$): δ = 8.53 (s, 1H, N**H**), 7.78 (dd, *J* = 7.6, 1.1 Hz, 1H), 7.55 (dd, *J* = 7.7, 1.1 Hz, 1H), 7.42 (t, *J* = 7.7 Hz, 1H), 5.25 (dd, *J* = 13.3, 5.2 Hz, 1H), 4.49 (d, *J* = 16.7 Hz, 1H), 4.40 – 4.28 (m, 1H), 2.97 – 2.77 (m, 2H), 2.42 (t, *J* = 7.1 Hz, 2H), 2.39 – 2.20 (m, 2H), 2.20 (t, *J* = 7.5 Hz, 2H), 1.64 – 1.52 (m, 4H), 1.48 – 1.39 (m, 11H), 1.36 – 1.28 (m, 6H) ppm.
**$^{13}$C-NMR** (101 MHz, $CDCl_3$): δ = 173.5, 171.4, 169.7, 169.2, 143.6, 134.8, 131.6, 128.5, 123.3, 119.8, 96.4, 80.2, 76.5, 51.9, 47.1, 35.6, 31.7, 29.4, 29.1, 29.04, 28.99, 28.7, 28.3, 25.2, 23.6, 19.6 ppm.
**HRMS** (ESI, pos): *m/z* $[M+Na]^+$ calculated for $[C_{28}H_{37}N_2O_5Na]^+$: 503.2516, found: 503.2511.
**IR** (KBr): $\tilde{\nu}$ = 3437 (s), 3086 (w), 2978 (w), 2929 (m), 2854 (m), 1716 (s), 1669 (s), 1608 (m), 1483 (m), 1454 (m), 1435 (m), 1369 (m), 1332 (m), 1235 (m), 1214 (m), 1151 (m), 1095 (w), 992 (w), 852 (w), 815 (w), 755 (m), 621 (w) $cm^{-1}$.
**UV / Vis** (DCM): $\lambda_{max}$ = 258, 228 nm.
**mp**: 225 °C.

**6-(2-(2,6-Dioxopiperidin-3-yl)-1-oxoisoindolin-4-yl)hex-5-ynoic acid (2a)**

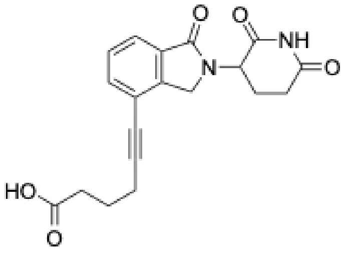


**9a** (65 mg, 0.16 mmol, 1.0 eq.) was dissolved in dry DCM (2 mL) and TFA (1 mL), and stirred for 3 h. After consumption of the starting material, 5 mL of 1M HCl were added and the product was extracted with EtOAc. The combined organic phases were washed with brine, dried over $Na_2SO_4$, filtered and concentrated *in vacuo*. The desired product **2a** was obtained as an off-white solid (50 mg, 89 %).

$R_f$ = 0.19 (10 % MeOH in DCM, *v/v*).

**$^1$H-NMR** (400 MHz, DMSO-$d_6$): δ = 10.98 (s, 1H), 7.71 (dd, *J* = 7.6, 1.3 Hz, 1H), 7.65 (dd, *J* = 7.7, 1.3 Hz, 1H), 7.52 (t, *J* = 7.6 Hz, 1H), 5.13 (dd, *J* = 13.3, 5.1 Hz, 1H), 4.46 (d, *J* = 17.8 Hz, 1H), 4.31 (d, *J* = 17.8 Hz, 1H), 2.97 – 2.85 (m, 1H), 2.64 – 2.55 (m, 1H), 2.55 – 2.51 (m, 2H), 2.45 (dd, *J* = 12.9, 4.5 Hz, 1H), 2.39 (t, *J* = 7.3 Hz, 2H), 2.06 – 1.99 (m, 1H), 1.85 – 1.76 (m, 2H) ppm.

**$^{13}$C-NMR** (75 MHz, DMSO-$d_6$): δ = 174.0, 172.9, 171.0, 167.7, 143.8, 134.2, 132.0, 128.6, 122.7, 118.7, 95.6, 76.8, 51.7, 47.0, 32.6, 31.2, 23.6, 22.4, 18.3 ppm.

**HRMS** (ESI, pos): *m/z* $[M+H]^+$ calculated for $[C_{19}H_{19}N_2O_5]^+$: 355.1288, found: 355.1293.

**IR** (KBr): ṽ = 3440 (m), 3085 (w), 2924 (w), 1707 (s), 1668 (s), 1607 (m), 1484 (w), 1454 (w), 1370 (w), 1333 (w), 1236 (m), 1183 (w), 1148 (w), 816 (w), 755 (m), 620 (w) $cm^{-1}$.

**UV / Vis** (MeOH): $\lambda_{max}$ = 254, 216 nm.

**mp**: 190 °C (decomposition).

**8-(2-(2,6-dioxopiperidin-3-yl)-1-oxoisoindolin-4-yl)oct-7-ynoic acid (2b) [14]**

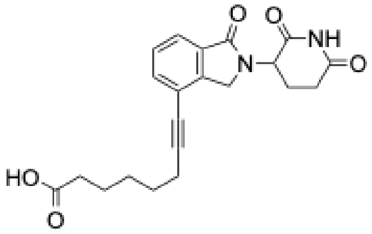


At 0 °C, TFA (1 mL) was added to a solution of **9b** (100 mg, 0.228 mmol, 1.0 eq.) in dry DCM (2 mL). The reaction mixture was warmed to room temperature and left to stir for 3 h. Afterwards, all volatiles were removed *in vacuo* and the residue was extracted with EtOAc (3 x 25 mL) and washed with 1M HCl (15 mL). The combined organic phases were dried over $Na_2SO_4$, filtered and concentrated *in vacuo*. Coevaporation with DCM (2 x) yielded **2b** as a yellow solid (89 mg, quant.).

**$^1$H-NMR** (400 MHz, DMSO-$d_6$): δ = 10.98 (s, 1H, N**H**), 7.70 (d, *J* = 7.5 Hz, 1H), 7.63 (d, *J* = 7.6 Hz, 1H), 7.51 (t, *J* = 7.6 Hz, 1H), 5.13 (dd, $^3J_{H-H}$ = 13.3, 5.1 Hz, 1H), 4.45 (d, $^2J_{H-H}$ = 17.7 Hz, 1H), 4.31 (d, $^2J_{H-H}$ = 17.7 Hz, 1H), 2.96 – 2.84 (m, 1H), 2.62 – 2.55 (m, 1H), 2.49 – 2.39 (m, 3H), 2.23 (t, *J* = 7.2 Hz, 2H), 2.05 – 1.97 (m, 1H), 1.62 – 1.49 (m, 4H), 1.49 – 1.37 (m, 2H) ppm.

**$^{13}$C-NMR** (76 MHz, DMSO-$d_6$): δ = 174.5, 172.9, 171.0, 167.7, 143.8, 134.1, 132.0, 128.6, 122.6, 118.9, 96.3, 76.5, 51.7, 47.0, 33.6, 31.2, 27.9, 27.8, 24.0, 22.4, 18.7 ppm.

**HRMS** (ESI, pos): *m/z* $[M+H]^+$ calculated for $[C_{21}H_{23}N_2O_5]^+$: 383.1601, found: 383.1608.

**IR** (KBr): ṽ = 3418 (br), 2933 (w), 2858 (w), 2227 (w), 1704 (s), 1670 (s), 1619 (m), 1484 (w), 1455 (m), 1433 (w), 1371 (w), 1332 (w), 1234 (w), 1208 (m), 1182 (w), 754 (w) $cm^{-1}$.

**UV / Vis** (MeOH): $\lambda_{max}$ = 298, 288, 256, 247, 224, 213 nm.

**mp**: 204 - 205°C.

**11-(2-(2,6-Dioxopiperidin-3-yl)-1-oxoisoindolin-4-yl)undec-10-ynoic acid (2c)**

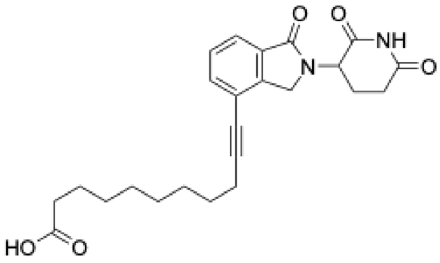

**9c** (70 mg, 0.15 mmol, 1.0 eq.) was dissolved in dry DCM (2 mL) and TFA (1 mL), and stirred for 3 h. After consumption of the starting material, 5 mL of 1M HCl were added and the product was extracted with EtOAc. The combined organic phases were washed with brine, dried over $Na_2SO_4$, filtered and concentrated *in vacuo*. The desired product **2c** was obtained as an off-white solid (51 mg, 82 %).

**$R_f$** = 0.22 (10 % MeOH in DCM, *v/v*).
**$^1$H-NMR** (300 MHz, DMSO-$d_6$): δ = 11.96 (s, 1H), 10.99 (s, 1H), 7.70 (d, *J* = 6.9 Hz, 1H), 7.63 (d, *J* = 7.0 Hz, 1H), 7.51 (t, *J* = 7.5 Hz, 1H), 5.14 (dd, *J* = 13.2, 5.1 Hz, 1H), 4.45 (d, *J* = 17.7 Hz, 1H), 4.30 (d, *J* = 17.7 Hz, 1H), 3.00 – 2.83 (m, 1H), 2.68 – 2.54 (m, 1H), 2.46 – 2.31 (m, 3H), 2.18 (t, *J* = 7.3 Hz, 2H), 2.08 – 1.99 (m, 1H), 1.64 – 1.36 (m, 6H), 1.36 – 1.21 (m, 6H) ppm.
**$^{13}$C-NMR** (75 MHz, DMSO-$d_6$): δ = 174.5, 172.8, 171.0, 167.7, 143.7, 134.1, 132.0, 128.6, 122.6, 118.9, 96.4, 76.4, 51.6, 47.0, 33.6, 31.2, 28.7, 28.5, 28.4, 28.3, 28.0, 24.5, 22.4, 18.7 ppm.
**HRMS** (ESI, pos): *m/z* $[M+H]^+$ calculated for $[C_{24}H_{29}N_2O_5]^+$: 425.2071, found: 425.2071.
**IR** (KBr): $\tilde{v}$ = 3438 (s), 3086 (w), 2929 (m), 2855 (m), 1709 (s), 1668 (s), 1483 (w), 1454 (w), 1435 (w), 1415 (w), 1371 (w), 1333 (w), 1235 (m), 1213 (m), 1182 (m), 1147 (w), 990 (w), 816 (w), 756 (m), 620 (w) $cm^{-1}$.
**UV / Vis** (MeOH): $\lambda_{max}$ = 256, 223 nm.
**mp**: 205 °C (decomposition).

**5-Nitro-2-phenoxybenzonitrile (11)**

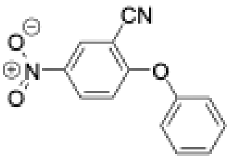

Synthesis of **11** was carried out essentially as described in the literature.[15] In brief, to a solution of 2-fluoro-5-nitrobenzonitrile **10** (100 mg, 0.602 mmol, 1.0 eq.) in dry DMF (1 mL) were added anhydrous $K_2CO_3$ (108 mg, 0.783 mmol, 1.3 eq.) and phenol (57 mg, 0.60 mmol, 1.0 eq.). The resulting suspension was heated to 80 °C for 2 h. After cooling to room temperature, water was added (10 mL) and the mixture was extracted with EtOAc (3 x 15 mL). The combined organic phases were washed with brine, dried over $Na_2SO_4$, filtered and concentrated *in vacuo* to yield **11** as an orange solid (127 mg, 91 %).

**$R_f$** = 0.45 (4:1 hexane / EtOAc , *v/v*).
**$^1$H-NMR** (300 MHz, $CDCl_3$): δ = 8.54 (d, *J* = 2.8 Hz, 1H), 8.29 (dd, *J* = 9.3, 2.7 Hz, 1H), 7.54 – 7.45 (m, 2H), 7.39 – 7.31 (m, 1H), 7.19 – 7.11 (m, 2H), 6.89 (d, *J* = 9.4 Hz, 1H) ppm.
**$^{13}$C-NMR** (75 MHz, $CDCl_3$): δ = 164.6, 153.3, 142.0, 130.8, 130.0, 129.7, 126.9, 120.9, 115.7, 114.0, 103.7 ppm.
**HRMS** (ESI, pos): *m/z* $[M+Na]^+$ calculated for $[C_{13}H_8N_2NaO_3]^+$: 263.0427, found: 263.0429.
**IR** (KBr): $\tilde{v}$ = 3102 (m), 3079 (s), 2239 (s), 1614 (s), 1579 (s), 1521 (s), 1479 (s), 1350 (s), 1274 (s), 1194 (s), 1163 (s), 1086 (s), 920 (s), 786 (s), 745 (s), 696 (s) $cm^{-1}$.
**UV / Vis** ($CHCl_3$): $\lambda_{max}$ = 298, 242 nm.
**mp**: 127 - 128°C.

**5-Amino-2-phenoxybenzonitrile (3)**

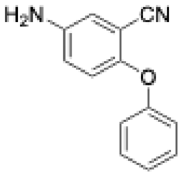


Synthesis of **3** was carried out essentially as described in the literature.[15] In brief, a suspension of **11** (125 mg, 0.521 mmol, 1.0 eq.) and iron powder (146 mg, 2.61 mmol, 5.0 eq.) in EtOH / AcOH / $H_2O$ (3 ml / 0.3 mL / 3 mL, *v/v*) was stirred for 1.5 h at 75 °C. After being cooled to room temperature, the volatiles were removed *in vacuo* and the residue was suspended in EtOAc. The precipitate was filtered and water was added to the filtrate. The organic and aqueous phases were seperated and the aqueous phase was extracted with EtOAc (2 x). The combined organic phases were washed with brine (1 x), dried over $Na_2SO_4$, filtered and concentrated *in vacuo*. The crude product was purified by column chromatography (3:2 hexane / EtOAc, *v/v*) to yield **3** as a yellow solid (95 mg, 87 %).

**$R_f$** = 0.30 (3:2 hexane / EtOAc, *v/v*).

**$^{1}$H-NMR** (400 MHz, $CD_3OD$): δ = 7.35 – 7.29 (m, 2H), 7.11 – 7.05 (m, 1H), 6.97 – 6.89 (m, 4H), 6.81 (d, *J* = 8.8 Hz, 1H) ppm.
**$^{13}$C-NMR** (101 MHz, $CD_3OD$): δ = 159.1, 150.7, 146.4, 130.9, 124.4, 122.4, 122.2, 118.8, 118.5, 117.2, 106.4 ppm.
**HRMS** (ESI, pos): *m/z* $[M+H]^+$ calculated for $[C_{13}H_{11}N_2O]^+$: 211.0866, found: 211.0872.
**IR** (KBr): ṽ = 3442 (s), 3357 (s), 3042 (w), 2231 (s), 1631 (s), 1590 (s), 1578 (s), 1498 (s), 1487 (s), 1442 (m), 1265 (s), 1228 (s), 1196 (s), 860 (s), 750 (s), 689 (s) $cm^{-1}$.
**UV / Vis** (MeOH): $\lambda_{max}$ = 338, 257, 220 nm.
**mp**: 66 - 67°C.

**Benzyl-4-hydroxybenzoate (12)**

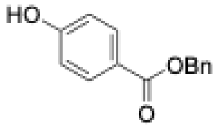


Synthesis of **12** was carried out essentially as described in the literature.[16] To a solution of 4-hydroxybenzoic acid (10.5 g, 76.2 mmol, 1.0 eq.) in dry DMF (115 mL) were added $KHCO_3$ (9.18 g, 91.7 mmol, 1.2 eq.) and benzyl bromide (9.8 mL, 90 mmol, 1.2 eq.). The resulting suspension was stirred at 40 °C for 5.5 h. Afterwards, water was added to the mixture, followed by an extraction with EtOAc (3 x). The combined organic phases were washed with sat. $NaHCO_3$ solution (1 x), dried over $Na_2SO_4$, filtered and concentrated *in vacuo*. The crude product was purified by column chromatography (3:1 - 1:1 hexane / EtOAc, *v/v*) to yield **12** as a colorless solid (14.7 g, 85 %).

**$R_f$** = 0.33 (3:1 hexane / EtOAc, *v/v*).
**$^{1}$H-NMR** (400 MHz, $CDCl_3$): δ = 8.03 – 7.95 (m, 2H), 7.47 – 7.31 (m, 5H), 6.92 – 6.85 (m, 2H), 6.81 – 6.66 (m, 1H, O**H**), 5.36 (s, 2H) ppm.
**$^{13}$C-NMR** (101 MHz, $CDCl_3$): δ = 167.1, 160.7, 136.1, 132.3, 128.7, 128.4, 128.2, 122.2, 115.5, 66.9 ppm.
**HRMS** (ESI, pos): *m/z* $[M+Na]^+$ calculated for $[C_{14}H_{13}NaO_3]^+$: 251.0679, found: 251.0671.
**IR** (KBr): ṽ = 3387 (br), 1685 (s), 1603 (s), 1586 (s), 1511 (m), 1281 (s), 1163 (s), 856 (m), 770 (m), 731 (m), 700 (m), 640 (m) $cm^{-1}$.
**UV / Vis** ($CHCl_3$): $\lambda_{max}$ = 256 nm.
**mp**: 112 - 113 °C (lit.: 110 °C).[16]

**Benzyl-4-(2-methoxy-2-oxoethoxy) benzoate (13)**

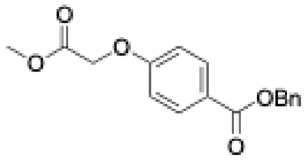

$K_2CO_3$ (16.4 g, 118 mmol, 1.9 eq.) and methyl bromoacetate (7.1 mL, 75 mmol, 1.2 eq.) were added to a solution of **12** (14.3 g, 62.5 mmol, 1.0 eq.) in dry DMF (100 mL). The resulting suspension was stirred at rt for 1 h. Water was added to the reaction mixture, followed by an extraction with EtOAc (3 x). The combined organic phases were washed with brine (1 x), dried over $Na_2SO_4$, filtered and concentrated *in vacuo*. The crude product was redissolved in hexane (3 x) and again concentrated *in vacuo* to yield **13** as a colorless solid (15.5 g, 83 %).

**$R_f$** = 0.37 (3:1 hexane / EtOAc, *v/v*).
**$^1$H-NMR** (400 MHz, $CDCl_3$): δ = 8.08 – 8.01 (m, 2H), 7.46 – 7.31 (m, 5H), 6.95 – 6.89 (m, 2H), 5.34 (s, 2H), 4.69 (s, 2H), 3.81 (s, 3H) ppm.
**$^{13}$C-NMR** (101 MHz, $CDCl_3$): δ = 168.8, 166.0, 161.6, 136.3, 131.9, 128.7, 128.3, 128.2, 123.8, 114.3, 66.6, 65.2, 52.5 ppm.
**HRMS** (ESI, pos): *m/z* $[M+H]^+$ calculated for $[C_{17}H_{17}O_5]^+$: 301.1071, found: 301.1070.
**IR** (KBr): $\tilde{v}$ = 3434 (br), 1763 (m), 1703 (m), 1607 (m), 1279 (s), 1175 (m), 850 (w), 771 (m), 700 (w) $cm^{-1}$.
**UV / Vis** ($CHCl_3$): $\lambda_{max}$ = 254 nm.
**mp**: 76 - 77 °C.

**4-(2-Methoxy-2-oxoethoxy) benzoic acid (4)**

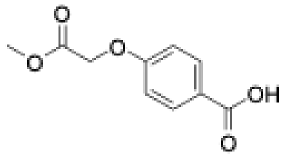

To a solution of **13** (15.3 g, 50.8 mmol, 1.0 eq.) in EtOH / EtOAc (1:1, 700 mL) was added Pd/C (10 wt%, 5.4 g, 50.9 mmol, 1.0 eq.) under $N_2$. The $N_2$ atmosphere was exchanged for $H_2$ and the mixture was stirred for 1 h. Upon completion of the reaction, the mixture was filtered through celite and washed with ethanol. The solvent was concentrated *in vacuo* to yield **4** as a colorless solid (15.3 g, 95 %).

**$^1$H-NMR** (400 MHz, $CD_3OD$): δ = 8.01 – 7.94 (m, 2H), 7.03 – 6.95 (m, 2H), 4.80 (s, 2H), 3.79 (s, 3H) ppm.
**$^{13}$C-NMR** (101 MHz, $CD_3OD$): δ = 170.8, 169.5, 163.2, 132.8, 125.1, 115.3, 65.9, 52.7 ppm.
**HRMS** (ESI, pos): *m/z* $[M+Na]^+$ calculated for $[C_{10}H_{10}NaO_5]^+$: 233.0420, found: 233.0425.
**IR** (KBr): ṽ = 3451 (br), 2963 (br), 2557 (m), 1740 (s), 1677 (s), 1604 (s), 1428 (s), 1293 (br), 1173 (s), 1073 (m), 1011 (s), 852 (m), 550 (m) $cm^{-1}$.
**UV / Vis** (MeOH): $\lambda_{max}$ = 250, 207 nm.
**mp**: 173 °C.

**Methyl 2-(4-((3-cyano-4-phenoxyphenyl)carbamoyl)phenoxy)acetate (14)**

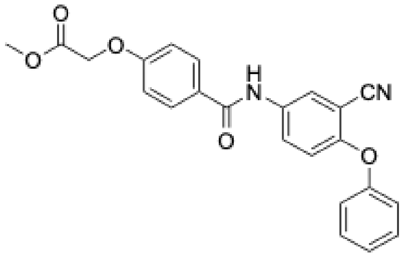

To a solution of **4** (1.25 g, 5.95 mmol, 1.0 eq.) in dry DMF (5 mL) were added EDC-HCl (1.20 g, 6.25 mmol, 1.05 eq.) and HOBt (803 mg, 5.95 mmol, 1.0 eq.) at 0 °C. After an activation time of 30 min at 0 °C and 30 min at rt, $NEt_3$ (3.3 mL, 24 mmol, 4.0 eq.) and **3** (1.25 g, 5.95 mmol, 1.0 eq.) were added. The reaction was stirred overnight, before 5 % LiCl-solution (50 mL) was added. The mixture was extracted with EtOAc (5 x 50 mL) and the combined organic phases were washed with brine (80 mL), dried over $Na_2SO_4$, filtered and concentrated *in vacuo*. The crude product was purified by column chromatography (0 - 50 % acetone in hexane, *v/v*) to yield **14** as a colorless solid (1.56 g, 65 %).

**$R_f$** = 0.45 (1:1 EtOAc / hexane, *v/v*).
**$^1$H-NMR** (400 MHz, acetone-$d_6$): δ = 9.69 (s, 1H, N**H**), 8.35 (d, *J* = 2.7 Hz, 1H), 8.06 – 7.94 (m, 3H), 7.50 – 7.40 (m, 2H), 7.30 – 7.20 (m, 1H), 7.16 – 7.11 (m, 2H), 7.09 – 7.05 (m, 2H), 7.03 (d, *J* = 9.1 Hz, 1H), 4.86 (s, 2H), 3.76 (s, 3H) ppm.
**$^{13}$C-NMR** (101 MHz, acetone-$d_6$): δ = 169.5, 165.9, 161.9, 157.2, 155.5, 136.4, 131.1, 130.3, 128.5, 128.4, 127.3, 125.4, 119.8, 119.8, 116.3, 115.2, 105.0, 65.6, 52.3 ppm.
**HRMS** (ESI, pos): *m/z* $[M+H]^+$ calculated for $[C_{23}H_{19}N_2O_5]^+$: 403.1288, found: 403.1280.
**IR** (KBr): ṽ = 3435 (br), 2236 (w), 1762 (m), 1660 (m), 1604 (m) 1508 (s), 1486 (s), 1404 (m), 1264 (s), 1225 (s), 1178 (m), 1071 (w), 691 (w) $cm^{-1}$.
**UV / Vis** ($CH_3CN$): $\lambda_{max}$ = 274, 212 nm.
**mp**: 178 °C.

**Methyl 2-(4-((3-(((*tert*-butoxycarbonyl)amino)methyl)-4-phenoxyphenyl)carbamoyl) phenoxy)acetate (15)**

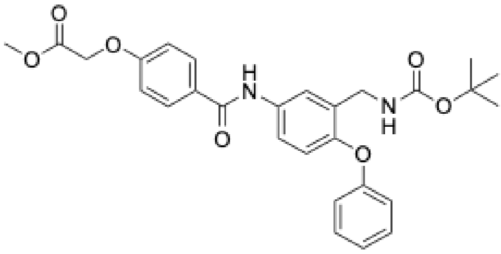

Synthesis of **15** was carried out according to the procedure described in the literature.[17] In brief, $NaBH_4$ (846 mg, 22.3 mmol, 7.0 eq.) was carefully added in small portions to a suspension of **14** (1.28 g, 3.18 mmol, 1.0 eq.), $NiCl_2$ x $H_2O$ (757 mg, 3.18 mmol, 1.0 eq.) and di-*tert*-butyl dicarbonate (1.5 mL, 6.4 mmol, 2.0 eq.) in dry MeOH (15 mL) at 0 °C. The resulting suspension was stirred at rt overnight. The solvent was removed *in vacuo* and the residue was purified by column chromatography (3:2 hexane / EtOAc, *v/v*) to yield **15** as a colorless foam (1.15 g, 72 %).

**$R_f$** = 0.23 (3:2 hexane / EtOAc, *v/v*).
**$^1$H-NMR** (400 MHz, $CDCl_3$): δ = 7.94 (s, 1H), 7.87 – 7.81 (m, 2H), 7.68 (d, *J* = 8.8 Hz, 1H), 7.46 (s, 1H, N**H**), 7.34 – 7.28 (m, 2H), 7.10 – 7.05 (m, 1H), 6.98 – 6.95 (m, 2H), 6.95 – 6.91 (m, 2H), 6.88 (d, *J* = 8.8 Hz, 1H), 5.00 (s, 1H, N**H**), 4.70 (s, 2H), 4.30 (d, *J* = 6.2 Hz, 2H), 3.82 (s, 3H), 1.41 (s, 9H) ppm.
**$^{13}$C-NMR** (101 MHz, $CDCl_3$): δ = 169.0, 165.2, 160.7, 157.7, 156.1, 151.1, 134.2, 131.1, 130.0, 129.2, 128.3, 123.2, 122.0, 121.3, 120.1, 117.9, 114.7, 79.6, 65.3, 52.6, 40.2, 28.5 ppm.
**HRMS** (ESI, pos): *m/z* $[M+Na]^+$ calculated for $[C_{28}H_{30}N_2NaO_7]^+$: 529.1945, found: 529.1936.
**IR** (KBr): ṽ = 3416 (br), 2977 (w), 1759 (s), 1694 (s), 1650 (s), 1606 (s), 1507 (s), 1487 (s), 1366 (m), 1250 (s), 1217 (s), 1177 (s), 1078 (w), 844 (m), 761 (m), 692 (w) $cm^{-1}$.
**UV / Vis** ($CHCl_3$): $\lambda_{max}$ = 278, 245 nm.
**mp**: 50 - 52°C.

**2-(4-((3-(((*Tert*-butoxycarbonyl)amino)methyl)-4-phenoxyphenyl)carbamoyl)phenoxy) acetic acid (16)**

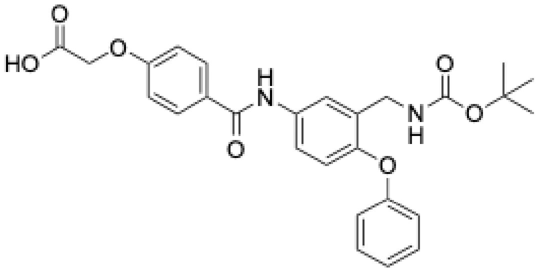

A suspension of **15** (1.04 g, 2.06 mmol, 1.0 eq.) in THF / 1M NaOH in water (1:1, 24 mL, *v/v*) was left to stir for 1 h at room temperature. After completion of the reaction, the mixture was acidified to pH 4 using 1M HCl. THF was evaporated under reduced pressure and the residue was extracted with EtOAc (3 x 25 mL). The combined organic phases were washed with brine (1 x 30 mL), dried over $Na_2SO_4$, filtered and concentrated *in vacuo*. After coevaporation with hexane (1 x) **16** was isolated as a colorless solid (1.13 g, 99 %).

**$^1$H-NMR** (400 MHz, $CD_3OD$): δ = 7.92 (d, *J* = 8.7 Hz, 2H), 7.67 (d, *J* = 2.6 Hz, 1H), 7.56 (dd, *J* = 8.7, 2.6 Hz, 1H), 7.33 (t, *J* = 7.8 Hz, 2H), 7.11 – 7.03 (m, 3H), 6.95 (d, *J* = 8.0 Hz, 2H), 6.86 (d, *J* = 8.7 Hz, 1H), 4.76 (s, 2H), 4.28 (s, 2H), 1.42 (s, 9H) ppm.
**$^{13}$C-NMR** (101 MHz, $CD_3OD$): δ = 172.2, 168.2, 162.4, 159.3, 158.4, 152.1, 136.0, 132.4, 130.9, 130.5, 129.0, 124.0, 123.0, 122.7, 120.7, 118.7, 115.5, 80.3, 65.9, 40.3, 28.8 ppm.
**HRMS** (ESI, pos): *m/z* $[M+Na]^+$ calculated for $[C_{27}H_{28}N_2NaO_7]^+$: 515.1789, found: 515.1794.
**IR** (KBr): ṽ = 3427 (br), 2978 (w), 1739 (m), 1691 (s), 1650 (s), 1606 (s), 1507 (s), 1488 (s), 1421 (m), 1367 (m), 1252 (s), 1219 (s), 1177 (s), 1073 (m), 844 (m), 761 (m), 691 (m) $cm^{-1}$.
**UV / Vis** (MeOH): $\lambda_{max}$ = 278, 206 nm.
**mp**: 119 - 123°C.

**1,2,3,4-Tetrahydroisoquinoline-6,7-diol hydrobromide (5)**

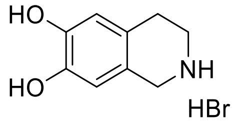


Synthesis of this compound according to the published procedure[18] has been described.[7]

***Tert*-butyl (5-(4-(2-(6,7-dihydroxy-3,4-dihydroisoquinolin-2(1*H*)-yl)-2-oxoethoxy) benzamido)-2-phenoxybenzyl)carbamate (17)**

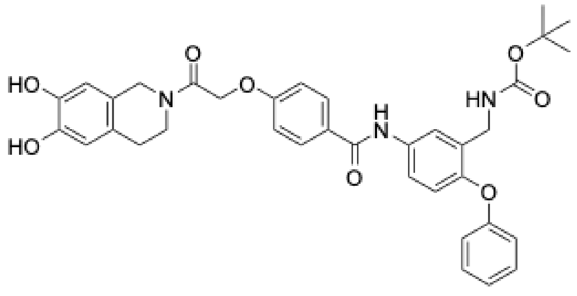


To a solution of **16** (1.98 g, 4.02 mmol, 1.0 eq.) in dry DMF (15 mL) were added HATU (1.68 g, 4.42 mmol, 1.1 eq.), DIPEA (3.5 mL, 20 mmol, 5.0 eq.) and **5** (989 mg, 4.02 mmol, 1.0 eq.). The mixture was stirred overnight at rt. Upon completion of the reaction, 5 % LiCl-solution (100 mL) was added, followed by an extraction with EtOAc (3 x 150 mL). The combined

organic phases were washed with 5 % LiCl-solution (1 x) and brine (1 x), dried over $Na_2SO_4$, filtered and concentrated *in vacuo*. The crude product was purified by column chromatography (5 % MeOH in DCM, *v/v*) to yield **17** as a colorless foam (1.19 g, 46 %).

$\mathbf{R_f}$ = 0.25 (5 % MeOH in DCM, *v/v*).
**$^1$H-NMR** (400 MHz, $CD_3OD$, rotameric mixture): δ = 7.94 – 7.87 (m, 2H), 7.66 (s, 1H), 7.58 – 7.53 (m, 1H), 7.36 – 7.28 (m, 2H), 7.11 – 7.03 (m, 3H), 6.94 (d, *J* = 8.1 Hz, 2H), 6.85 (d, *J* = 8.7 Hz, 1H), 6.62 – 6.55 (m, 2H, R1/R2), 4.99 – 4.93 (m, 2H), 4.58 (s, 1H, R2), 4.55 (s, 1H, R1), 4.28 (s, 2H), 3.75 (t, *J* = 6.0 Hz, 1H, R2), 3.71 (t, *J* = 6.1 Hz, 1H, R1), 2.79 (t, *J* = 5.9 Hz, 1H, R1), 2.70 (t, *J* = 6.0 Hz, 1H, R2), 1.42 (s, 9H) ppm.
**$^{13}$C-NMR** (101 MHz, $CD_3OD$, rotameric mixture): δ = 168.83 (**C**=O, R2), 168.80 (**C**=O, R1), 168.2, 162.5, 159.3, 158.4, 152.1, 145.6 (R2), 145.4 (R1), 145.3 (R1), 145.2 (R2), 136.0, 132.4, 130.9, 130.55 (R1), 130.51 (R2), 129.01 (R1), 128.95 (R2), 126.9 (R2), 126.4 (R1), 124.7 (R1), 124.4 (R2), 123.9, 123.0, 122.7, 120.7, 118.7, 116.1 (R2), 116.0 (R1), 115.7 (R2), 115.6 (R1), 114.0 (R1), 113.9 (R2), 80.3, 67.7 (R1), 67.6 (R2), 46.9 (R2), 45.1 (R1), 44.1 (R1), 41.8 (R2), 40.4, 29.5 (R1), 28.8, 28.5 (R2) ppm.
**HRMS** (ESI, pos): *m/z* $[M+Na]^+$ calculated for $[C_{36}H_{37}N_3NaO_8]^+$: 662.2473, found: 662.2487.
**IR** (KBr): $\tilde{\nu}$ = 3423 (br), 2978 (w), 1644 (s), 1607 (s), 1528 (m), 1506 (m), 1488 (s), 1454 (w), 1366 (w), 1280 (w), 1251 (m), 1219 (m), 1173 (m), 845 (w), 760 (w), 692 (w) $cm^{-1}$.
**UV / Vis** (MeOH): $\lambda_{max}$ = 278, 205 nm.
**mp**: 122 - 126°C.

***Tert*-butyl (5-(4-(2-(6,7-bis((diethoxyphosphoryl)oxy)-3,4-dihydroisoquinolin-2(1*H*)-yl)-2-oxoethoxy)benzamido)-2-phenoxybenzyl)carbamate (18)**

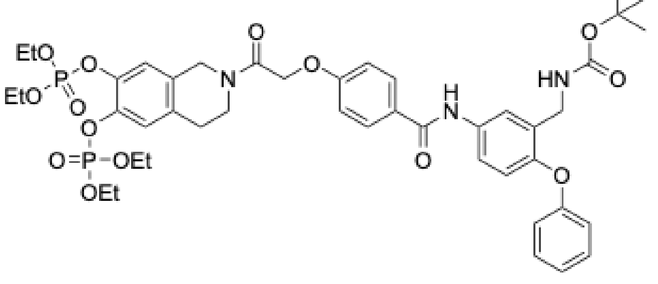


To a solution of **17** (1.18 g, 1.84 mmol, 1.0 eq.), $CCl_4$ (1.80 mL, 18.4 mmol, 10 eq.), DIPEA (1.25 mL, 7.36 mmol, 4.0 eq.) and a catalytic amount of DMAP in dry $CH_3CN$ (10 mL) was added diethyl phosphite (711 µL, 5.52 mmol, 3.0 eq.) at 0 °C. The reaction mixture was stirred at 0 °C for 30 min, followed by 30 min at rt. Upon completion of the reaction, $KH_2PO_4$ (50 mL, 0.5 M) was added and the resulting suspension was extraced with EtOAc (3 x). The combined

organic layers were washed with brine (1 x), dried over $Na_2SO_4$, filtered and concentrated *in vacuo*. The crude product was purified by column chromatography (5 % MeOH in EtOAc, *v*/*v*) to yield **18** as a colorless foam (1.37 g, 82 %).

**R$_f$** = 0.30 (5 % MeOH in EtOAc, *v/v*).
**$^1$H-NMR** (400 MHz, $CDCl_3$, rotameric mixture): δ = 8.41 – 7.89 (m, 1H, N**H**), 7.83 (d, *J* = 8.7 Hz, 1H, R1), 7.78 (d, *J* = 8.6 Hz, 1H, R2), 7.73 – 7.63 (m, 1H), 7.62 – 7.46 (m, 1H), 7.35 – 7.28 (m, 2H), 7.22 – 7.13 (m, 2H, R1/R2), 7.10 – 7.04 (m, 1H), 7.04 – 7.01 (m, 1H), 6.96 – 6.92 (m, 2H), 6.92 – 6.90 (m, 1H), 6.90 – 6.86 (m, 1H), 4.96 (s, 1H, N**H**), 4.84 (s, 1H, R2), 4.83 (s, 1H, R1), 4.68 (s, 2H), 4.34 – 4.29 (m, 2H), 4.29 – 4.18 (m, 8H), 3.82 – 3.73 (m, 2H), 2.86 (t, *J* = 5.8 Hz, 1H, R1), 2.68 (t, *J* = 6.0 Hz, 1H, R2), 1.41 (s, 9H), 1.39 – 1.33 (m, 12H) ppm.
**$^{31}$P-NMR** (162 MHz, $CDCl_3$, rotameric mixture): δ = -6.24 (R1), -6.33 (R2), -6.38 (R1/R2) ppm.
**$^{13}$C-NMR** (101 MHz, $CDCl_3$, rotameric mixture): δ = 166.6 (**C**=O, R1), 166.5 (**C**=O, R2), 165.5 (R1), 165.3 (R2), 160.5 (R1), 160.0 (R2), 157.7 (R2), 157.6 (R1), 156.0 (R1), 155.9 (R2), 150.8 (R1), 150.6 (R2), 140.3 – 139.7 (R1/R2), 134.6 (R2), 134.1 (R1), 132.1 (R1), 131.3 (R2), 130.9 (R1), 130.8 (R2), 130.1 (R2), 130.0 (R1), 129.88 (R1), 129.85 (R2), 129.3 (R1), 129.2 (R2), 128.0 (R2), 128.0 (R1), 123.02 (R1), 122.95 (R2), 121.9 (R2), 121.8 (R1), 121.3 (R1/R2), 121.2, 120.1, 119.5 (R1), 118.9 (R2), 117.7 (R1), 117.6 (R2), 114.54 (R2), 114.5 (R1), 79.6 (R1), 79.5 (R2), 67.7 (R1), 67.6 (R2), 65.3 – 64.8 (m, R1/R2), 46.7 (R2), 44.1 (R1), 42.8 (R2), 40.4 (R1), 40.2 (R2), 40.1 (R1), 29.0 (R2), 28.4, 27.6 (R1), 16.4 – 16.1 (m, R1/R2) ppm.
**HRMS** (ESI, pos): *m/z* $[M+Na]^+$ calculated for $[C_{44}H_{55}N_3NaO_{14}P_2]^+$: 934.3051, found: 934.3067.
**IR** (KBr): $\tilde{v}$ = 3417 (br), 2980 (w), 1712 (m), 1656 (s), 1606 (m), 1542 (m), 1508 (s), 1487 (s), 1454 (w), 1366 (w), 1277 (s), 1251 (s), 1217 (s), 1167 (s), 1032 (s), 972 (m), 894 (m), 761 (w) $cm^{-1}$.
**UV / Vis** ($CHCl_3$): $\lambda_{max}$ = 277 nm.
**mp**: 77 - 78°C.

**(5-(4-(2-(6,7-Bis((diethoxyphosphoryl)oxy)-3,4-dihydroisoquinolin-2(1*H*)-yl)-2-oxoethoxy)benzamido)-2-phenoxyphenyl)methanaminium chloride (19)**

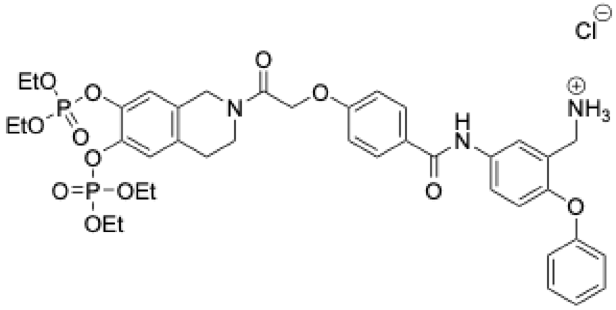

**Method 1**: A Schlenk flask containing HCl in dioxane (4 M, 27.5 mL, 110 mmol, 80 eq.) was cooled to 0 °C and **18** (1.26 g, 1.38 mmol, 1.0 eq.) was added. The ice bath was removed after 5 min and the mixture was left to stir for 1 h. Afterwards, the volatiles were removed *in vacuo* to yield the hydrochloride salt **19** as a colorless foam (1.15 g, 98 %).

$\mathbf{R_f}$ = 0.25 (7 % MeOH in DCM, *v/v*).
**$^1$H-NMR** (400 MHz, $CD_3OD$, rotameric mixture): δ = 7.99 – 7.96 (m, 1H, R1/R2), 7.96 – 7.91 (m, 2H, R1/R2), 7.57 – 7.52 (m, 1H, R1/R2), 7.43 (t, *J* = 7.9 Hz, 2H), 7.29 – 7.18 (m, 3H, R1/R2), 7.14 – 7.07 (m, 4H), 6.88 (d, *J* = 8.9 Hz, 1H), 5.04 (s, 2H), 4.76 (s, 1H, R2), 4.71 (s, 1H, R1), 4.33 – 4.22 (m, 8H), 4.19 (s, 2H), 3.85 – 3.77 (m, 2H, R1/R2), 2.97 (t, *J* = 5.5 Hz, 1H, R1), 2.86 (t, *J* = 5.7 Hz, 1H, R2), 1.37 (t, *J* = 7.1 Hz, 12H) ppm.
**$^{31}$P-NMR** (162 MHz, $CD_3OD$, rotameric mixture): δ = -7.29 (R2), -7.34 (R1) ppm.
**$^{13}$C-NMR** (101 MHz, $CD_3OD$, rotameric mixture): δ = 166.75 (R2), 166.74 (R1), 165.99 (R1), 165.96 (R2), 160.44 (R1), 160.39 (R2), 155.6, 151.5, 139.4 – 138.9 (m), 133.6, 132.1 (R2), 132.7 (R1), 130.0 (R1), 129.7 (R2), 129.1, 128.51 (R1), 128.48 (R2), 126.4 (R1), 126.3 (R2), 123.3, 123.0, 122.84, 122.76, 120.6 (R2), 120.4 (R1), 118.6 (R1), 118.4 (R2), 118.1, 117.1, 113.54 (R2), 113.48 (R1), 65.2, 64.53 (d, $^2J_{C\text{-}P}$ = 6.3 Hz, R2), 64.46 (d, $^2J_{C\text{-}P}$ = 6.2 Hz, R1), 44.4 (R2), 42.6 (R1), 41.1 (R1), 38.8 (R2), 37.6, 27.5 (R1), 26.5 (R2), 14.4 (R1), 14.3 (R2) ppm.
**HRMS** (ESI, pos): *m/z* $[M+H]^+$ calculated for $[C_{39}H_{48}N_3O_{12}P_2]^+$: 812.2708, found: 812.2721.
**IR** (KBr): $\tilde{v}$ = 3444 (br), 2986 (w), 1653 (s), 1605 (m), 1546 (w), 1508 (s), 1488 (s), 1423 (w), 1308 (m), 1259 (m), 1222 (s), 1033 (s), 973 (m), 895 (m), 760 (w) $cm^{-1}$.
**UV / Vis** (MeOH): $\lambda_{max}$ = 276, 208 nm.
**mp**: 113 - 115°C.

**(5-(4-(2-(6,7-Bis((diethoxyphosphoryl)oxy)-3,4-dihydroisoquinolin-2(1*H*)-yl)-2-oxoethoxy)benzamido)-2-phenoxyphenyl)methanamine (19a)**

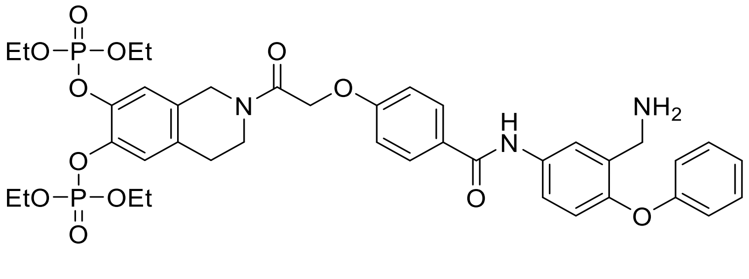


In an alternative procedure to remove the *N*-Boc group, **18** (300 mg, 0.329 mmol) was dissolved in a mixture of dry DCM and TFA (2 mL, 1/1, *v*/*v*) and stirred for 1 h at room temperature. After full consumption of the starting material, the pH value of the solution was adjusted to pH~7 and the product was extracted with ethyl acetate. The combined organic extracts were washed with brine, dried over $Na_2SO_4$ and all volatiles were removed under

reduced pressure. The crude product was purified by column chromatography (10 % MeOH in DCM, *v*/*v*) and the free amine **19a** was obtained as a colourless solid (167 mg, 63 %).

**$R_f$** = 0.11 (10 % MeOH in DCM, *v*/*v*).
**$^1$H-NMR** (400 MHz, $CD_3OD$, rotameric mixture): δ = 7.95 – 7.89 (m, 2H, R1/R2), 7.79 – 7.75 (m, 1H, R1/R2), 7.59 – 7.53 (m, 1H, R1/R2), 7.40 – 7.32 (m, 2H, R1/R2), 7.27 – 7.20 (m, 2H, R1/R2), 7.14 – 7.04 (m, 3H, R1/R2), 7.02 – 6.96 (m, 2H, R1/R2), 6.87 (d, *J* = 8.8 Hz, 1H), 5.00 (s, 2H), 4.75 (s, 1H, R2), 4.69 (s, 1H, R1), 4.34 – 4.21 (m, 8H), 3.89 (s, 2H), 3.84 – 3.76 (m, 2H, R1/R2), 2.95 (t, *J* = 5.9 Hz, 1H, R1), 2.84 (t, *J* = 6.0 Hz, 1H, R2), 1.43 – 1.31 (m, 12H) ppm.
**$^{31}$P-NMR** (162 MHz, $CD_3OD$, rotameric mixture): δ = - 7.24 (R2), -7.28 (R1), -7.33 (R1/R2) ppm.
**$^{13}$C-NMR** (75 MHz, $CD_3OD$, rotameric mixture): δ = 168.9, 168.2, 162.6 (R1), 162.5 (R2), 158.0, 153.4, 141.7 – 140.9 (m), 135.8, 134.2 (R2), 133.8 (R1), 132.0 (R1), 131.9 (R2), 131.2, 130.6 (R1), 130.5 (R2), 128.74 (R1), 128.68 (R2), 126.8, 125.1, 124.9, 124.6, 122.7 (R2), 122.5 (R1), 120.7 (R1), 120.5 (R2), 119.9, 119.6, 115.72 (R2), 115.67 (R1), 67.6, 66.6 (d, *J* = 6.3 Hz, R1/R2), 46.7 (R2), 44.8 (R1), 43.4 (R1), 41.1 (R2), 40.3, 29.6 (R1), 28.6 (R2), 16.5 (R1), 16.4 (R2) ppm.
**HRMS** (ESI, pos): *m*/*z* $[M+H]^+$ calculated for $[C_{39}H_{48}N_3O_{12}P_2]^+$: 812.2708, found: 812.2738.
**IR** (KBr): ṽ = 3444 (s), 1659 (s), 1605 (m), 1547 (w), 1508 (s), 1488 (s), 1421 (w), 1371 (w), 1308 (m), 1278 (m), 1220 (s), 1164 (m), 1033 (s), 972 (m), 894 (m), 855 (w), 761 (w), 693 (w) $cm^{-1}$.
**UV/Vis** (MeOH): $\lambda_{max}$ = 276, 205 nm.
**mp:** 92 °C.

**2-(2-(4-((3-((6-(2-(2,6-Dioxopiperidin-3-yl)-1-oxoisoindolin-4-yl)hex-5-ynamido)methyl)-4-phenoxyphenyl)carbamoyl)phenoxy)acetyl)-1,2,3,4-tetrahydroisoquinoline-6,7-diyl tetraethyl bis(phosphate) (20a)**

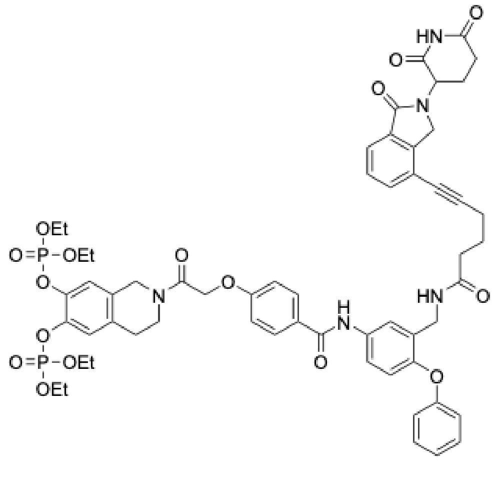

To a solution of **19a** (96 mg, 0.12 mmol, 1.2 eq.) in dry DMF (1.5 mL) were added **2a** (35 mg, 0.099 mmol, 1.0 eq.), HATU (45 mg, 0.12 mmol, 1.2 eq.) and DIPEA (84 µL, 0.49 mmol, 5.0 eq.). The resulting suspension was stirred at rt overnight, followed by the addition of 5 % LiCl-solution. The mixture was extracted with EtOAc, washed with brine, dried over $Na_2SO_4$, filtered and concentrated *in vacuo*. Purification of the crude product by column chromatography (5 % MeOH in DCM, *v/v*) yielded **20a** as a colorless solid (65 mg, 57 %).

**$R_f$** = 0.31 (5 % MeOH in DCM, *v/v*).
**$^1$H-NMR** (400 MHz, $CD_3OD$, rotameric mixture): δ = 7.88 – 7.81 (m, 2H), 7.78 – 7.74 (m, 1H), 7.70 (dd, *J* = 7.7, 1.1 Hz, 1H), 7.58 – 7.53 (m, 1H), 7.49 – 7.41 (m, 2H), 7.34 – 7.29 (m, 2H), 7.29 – 7.19 (m, 2H), 7.08 – 7.01 (m, 2H), 7.01 – 6.96 (m, 1H), 6.96 – 6.91 (m, 2H), 6.86 (d, *J* = 8.7 Hz, 1H), 5.10 (dd, *J* = 13.3, 5.1 Hz, 1H), 4.96 (s, 2H), 4.74 (s, 1H, R2), 4.70 (s, 1H, R1), 4.46 – 4.42 (m, 2H), 4.40 (s, 2H), 4.33 – 4.21 (m, 8H), 3.84 – 3.74 (m, 2H, R1/R2), 2.95 (t, *J* = 6.1 Hz, 1H, R1), 2.88 – 2.80 (m, 2H, R2), 2.76 – 2.68 (m, 1H), 2.52 (t, *J* = 7.1 Hz, 2H), 2.45 (dd, *J* = 12.9, 4.7 Hz, 1H), 2.39 (t, *J* = 7.3 Hz, 2H), 2.15 – 2.07 (m, 1H), 1.97 – 1.88 (m, 2H), 1.42 – 1.33 (m, 12H) ppm.
**$^{31}$P NMR** (162 MHz, $CD_3OD$, rotameric mixture) δ -7.23 (R2), -7.28 (R1), -7.34 (R1/R2) ppm.
**$^{13}$C-NMR** (76 MHz, $CD_3OD$, rotameric mixture): δ = 175.2, 174.5, 172.1, 171.0, 168.8, 168.0, 162.3 (R1), 162.2 (R2), 159.1, 152.0, 145.3, 141.5 – 140.9 (m), 136.0, 135.8, 134.1 (R2), 133.7 (R1), 132.8, 132.0 (R1), 131.8 (R2), 131.5, 130.8, 130.5 (R1), 130.4 (R2), 129.5, 128.9 (R1), 128.8 (R2), 123.9, 123.7, 123.3, 122.7, 122.6 (R2), 122.4 (R1), 120.82, 120.79, 120.6 (R1), 120.5 (R2), 118.6, 115.5, 96.4, 77.8, 67.6, 66.6, 66.5, 54.7, 53.6, 46.7 (R2), 44.8 (R1), 43.3 (R1), 41.0 (R2), 39.3, 35.9, 32.3, 29.6 (R1), 28.6 (R2), 25.8, 24.0, 19.7, 16.5, 16.4 ppm.
**HRMS** (ESI, pos): *m/z* $[M+H]^+$ calculated for $[C_{58}H_{64}N_5O_{16}P_2]^+$: 1148.3818, found: 1148.3827.
**IR** (KBr): $\tilde{\nu}$ = 3444 (s), 3070 (w), 2923 (s), 2851 (m), 2465 (w), 2228 (w), 1696 (s), 1661 (s), 1542 (m), 1508 (s), 1487 (s), 1454 (m), 1372 (m), 1276 (s), 1219 (s), 1170 (m), 1110 (m), 1033 (s), 973 (s), 895 (m), 846 (w), 818 (w), 753 (m), 693 (w), 618 (w), 519 (w), 482 (w) $cm^{-1}$.
**UV / Vis** (DCM): $\lambda_{max}$ = 276, 256 nm.

**2-(2-(4-((3-((8-(2-(2,6-Dioxopiperidin-3-yl)-1-oxoisoindolin-4-yl)oct-7-ynamido)methyl)-4-phenoxyphenyl)carbamoyl)phenoxy)acetyl)-1,2,3,4-tetrahydroisoquinoline-6,7-diyl tetraethyl bis(phosphate) (20b)**

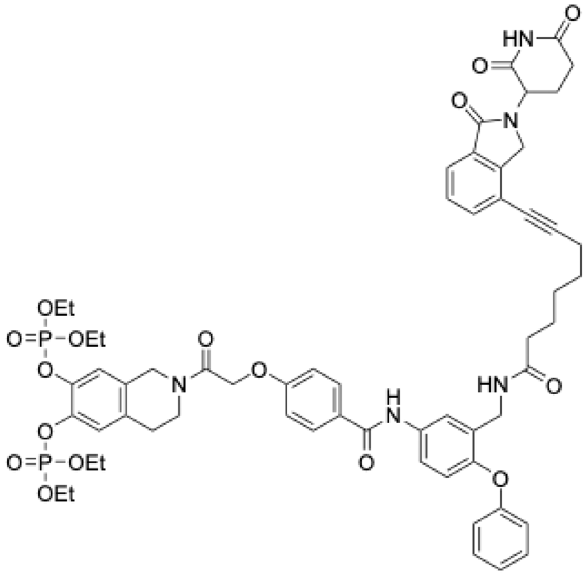

To a solution of **19** (178 mg, 0.210 mmol, 1.0 eq.) in dry DMF (3 mL) were added **2b** (79 mg, 0.21 mmol, 1.0 eq.), HATU (87 mg, 0.23 mmol, 1.1 eq.) and DIPEA (191 µL, 1.10 mmol, 5.0 eq.). The resulting suspension was stirred at rt overnight, followed by the addition of 5 % LiCl-solution (60 mL) and extraction with EtOAc (3 x 50 mL). The combined organic phases were washed with 5 % LiCl-solution (1 x 100 mL) and brine (1 x 100 mL), dried over $Na_2SO_4$, filtered and concentrated *in vacuo*. The crude product was purified by column chromatography (0 - 5 % MeOH in DCM, *v/v*) to yield **20b** as a colorless solid (156 mg, 63 %).

**$R_f$** = 0.33 (5 % MeOH in DCM, *v/v*).

**$^1$H-NMR** (400 MHz, $CD_2Cl_2$, rotameric mixture, recorded at 0°C): δ = 9.52 – 9.43 (m, 1H, N**H**), 9.13 – 8.98 (m, 1H, N**H**), 7.81 (d, *J* = 8.6 Hz, 1H, R1), 7.77 (d, *J* = 8.6 Hz, 1H, R2), 7.72 – 7.66 (m, 1H), 7.66 – 7.63 (m, 1H), 7.53 (dd, *J* = 8.8, 2.5 Hz, 1H), 7.49 (d, *J* = 7.6 Hz, 1H), 7.42 – 7.34 (m, 1H), 7.32 – 7.24 (m, 2H), 7.20 – 7.13 (m, 1H), 7.12 – 7.05 (m, 1H), 7.04 – 7.00 (m, 1H), 6.92 – 6.86 (m, 3H), 6.84 – 6.77 (m, 2H), 6.70 – 6.61 (m, 1H, N**H**), 5.11 (dd, *J* = 13.5, 5.2 Hz, 1H), 4.81 – 4.74 (m, 2H), 4.60 (s, 2H), 4.41 – 4.26 (m, 4H), 4.25 – 4.14 (m, 8H), 3.72 (t, *J* = 6.0 Hz, 1H, R2), 3.64 (t, *J* = 5.9 Hz, 1H, R1), 2.82 (t, *J* = 5.8 Hz, 1H, R1), 2.79 – 2.71 (m, 2H), 2.67 (t, *J* = 5.9 Hz, 1H, R2), 2.38 (t, *J* = 6.8 Hz, 3H), 2.19 – 2.12 (m, 3H), 1.60 – 1.46 (m, 4H), 1.43 – 1.35 (m, 2H), 1.35 – 1.30 (m, 12H) ppm.

**$^{31}$P-NMR** (162 MHz, $CD_2Cl_2$, rotameric mixture): δ = -6.43 (R1), -6.45 (R2), -6.49 (R1), -6.51 (R2) ppm.

**$^{13}$C-NMR** (101 MHz, $CD_2Cl_2$, rotameric mixture, recorded at 0°C): δ = 173.53 (R1), 173.46 (R2), 172.5 (R1), 172.4 (R2), 170.73 (R1), 170.70 (R2), 169.1, 166.58 (R1), 166.56 (R2), 165.6 (R2), 165.5 (R1), 160.8 (R1), 160.5 (R2), 157.9 (R2), 157.9 (R1), 150.4 (R1), 150.3 (R2), 144.2 (R1), 144.1 (R2), 140.4 – 139.7 (m, R1/R2), 135.2 (R2), 135.0 (R1), 134.4, 132.6 (R2), 131.9 (R1), 131.8, 130.6, 130.5 (R1), 130.09 (R2), 130.0, 129.5 (R1), 129.5 (R2), 128.5, 127.8 (R1),

127.7 (R2), 123.09 (R1), 123.06 (R2), 122.9, 121.93 (R2), 121.91 (R1), 121.8 (R2), 121.4 (R1), 121.2, 120.02 (R2), 119.99 (R1), 119.7, 119.5 (R1), 119.2 (R2), 117.7 (R1), 117.6 (R2), 114.5 (R2), 114.4 (R1), 96.5, 76.53 (R1), 76.50 (R2), 67.0 (R2), 66.9 (R1), 65.31 (t, $^{2}J_{C\text{-}P}$ = 5.8 Hz, R1/R2), 52.0, 47.3, 46.2 (R2), 44.0 (R1), 42.5 (R1), 40.1 (R2), 38.7, 36.5, 31.7 (R1), 28.9, 28.6, 28.4 (R2), 28.3 (R1), 27.8 (R2), 25.5, 23.3, 19.4, 16.2 (R2), 16.2 (R1) ppm.

**HRMS** (ESI, pos): *m/z* $[M+H]^+$ calculated for $[C_{60}H_{68}N_5O_{16}P_2]^+$: 1176.4131, found: 1176.4138.

**IR** (KBr): ṽ = 3422 (br), 2932 (w), 2226 (w), 1696 (m), 1654 (s), 1606 (m), 1544 (w), 1508 (m), 1486 (m), 1266 (m), 1216 (m), 1033 (s), 973 (w), 846 (m), 753 (w) $cm^{-1}$.

**UV / Vis** ($CHCl_3$): $\lambda_{max}$ = 278, 258 nm.

**mp**: 94 - 99°C.

**2-(2-(4-((3-((11-(2-(2,6-Dioxopiperidin-3-yl)-1-oxoisoindolin-4-yl)undec-10-ynamido) methyl)-4-phenoxyphenyl)carbamoyl)phenoxy)acetyl)-1,2,3,4-tetrahydroisoquinoline-6,7-diyl tetraethyl bis(phosphate) (20c)**

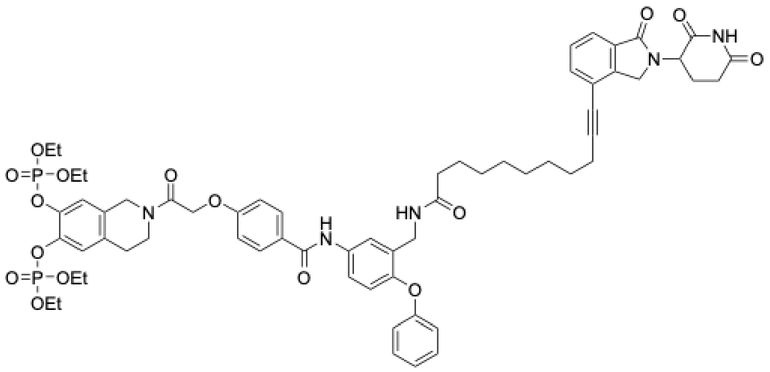


To a solution of **19a** (87 mg, 0.11 mmol, 1.2 eq.) in dry DMF (1.5 mL) were added **2c** (38 mg, 0.090 mmol, 1.0 eq.), HATU (41 mg, 0.11 mmol, 1.2 eq.) and DIPEA (76 µL, 0.45 mmol, 5.0 eq.). The resulting suspension was stirred at rt overnight, before 5 % LiCl-solution was added. The crude product was extracted with EtOAc, washed with brine, dried over $Na_2SO_4$, filtered and concentrated *in vacuo*. Purification by column chromatography (5 % MeOH in DCM, *v/v*) yielded **20c** as a colorless solid (68 mg, 62 %).

**$R_f$** = 0.35 (5 % MeOH in DCM, *v/v*).

**$^1$H-NMR** (400 MHz, $CD_3OD$, rotameric mixture): δ = 8.10 – 8.01 (m, 1H, N**H**), 7.94 – 7.87 (m, 2H), 7.73 – 7.69 (m, 1H), 7.68 – 7.65 (m, 1H), 7.56 (d, *J* = 7.6 Hz, 1H), 7.52 (dt, *J* = 8.8, 2.9 Hz, 1H), 7.44 (t, *J* = 7.6 Hz, 1H), 7.33 – 7.27 (m, 2H), 7.25 – 7.17 (m, 2H, R1/R2), 7.09 – 7.00 (m, 3H), 6.93 (d, *J* = 7.9 Hz, 2H), 6.86 (d, *J* = 8.8 Hz, 1H), 5.14 (dd, *J* = 13.4, 5.2 Hz, 1H), 4.93 (s, 2H), 4.72 (s, 1H, R2), 4.69 (s, 1H, R1), 4.45 (s, 2H), 4.42 – 4.37 (m, 2H), 4.32 – 4.20 (m, 8H), 3.85 – 3.75 (m, 2H), 2.93 (t, *J* = 6.6 Hz, 1H, R2), 2.89 – 2.74 (m, 3H, R1), 2.53 – 2.45 (m, 1H),

2.41 (t, $J$ = 7.0 Hz, 2H), 2.22 – 2.12 (m, 3H), 1.66 – 1.50 (m, 4H), 1.49 – 1.39 (m, 2H), 1.39 – 1.33 (m, 12H), 1.33 – 1.26 (m, 6H) ppm.

**$^{31}$P-NMR** (162 MHz, $CD_3OD$, rotameric mixture) δ = -7.27 (R2), -7.32 (R1), -7.37 (R1/R2) ppm.

**$^{13}$C-NMR** (76 MHz, $CD_3OD$, rotameric mixture): δ = 175.9, 174.2, 171.8, 170.9, 168.5, 167.7, 162.02 (R1), 161.94 (R2), 158.9, 151.89, 144.9, 141.5 – 140.7 (m), 135.6, 133.8, 133.2, 132.6, 131.6 (R1), 131.3 (R2), 131.2, 130.6, 130.40 (R1), 130.35 (R2), 129.4, 128.8 (R1), 128.7 (R2), 123.8, 123.5, 123.4, 122.6, 122.5 (R2), 122.2 (R1), 120.8, 120.6, 120.4 (R1), 120.2 (R2), 118.5, 115.43 (R2), 115.37 (R1), 97.3, 77.1, 67.6, 66.4, 66.3, 54.5, 53.3, 46.7 (R2), 44.7 (R1), 43.2 (R1), 40.9 (R2), 39.2, 36.9, 32.2, 30.2, 30.0, 29.8, 29.7, 29.5 (R1), 29.4, 28.5 (R2), 26.7, 24.0, 20.0, 16.44, 16.35 ppm.

**IR** (KBr): ṽ = 3435 (s), 2984 (w), 2928 (m), 2853 (m), 1698 (s), 1658 (s), 1605 (m), 1542 (w), 1508 (s), 1487 (s), 1454 (m), 1371 (m), 1278 (s), 1217 (s), 1169 (m), 1112 (w), 1033 (s), 974 (m), 894 (m), 817 (w), 753 (m), 693 (m) $cm^{-1}$.

**UV / Vis** (MeOH): $\lambda_{max}$ = 257, 206 nm.

**2-(2-(4-((3-((6-(2-(2,6-Dioxopiperidin-3-yl)-1-oxoisoindolin-4-yl)hex-5-ynamido)methyl)-4-phenoxyphenyl)carbamoyl)phenoxy)acetyl)-1,2,3,4-tetrahydroisoquinoline-6,7-diyl bis(dihydrogen phosphate) (1a)**

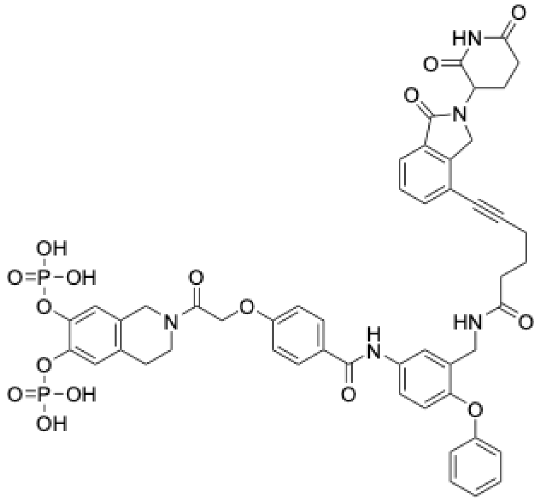


Synthesis of **1a** was carried out essentially as described in the literature.[7] In brief, **20a** (17 mg, 0.015 mmol, 1.0 eq.) was dissolved in dry DCM (1 mL) and cooled to 0 °C. A solution of TMSI in DCM (1M, 120 µL, 0.120 mmol, 8.0 eq.) and BSTFA (48 µL, 0.18 mmol, 12 eq.) were added and the mixture was left to stir for 10 min at 0 °C. After completion of the reaction indicated by TLC, all volatiles were removed *in vacuo*. The crude product was purified by reversed-phase

column chromatography (1:1 $CH_3CN$ / $H_2O$, *v/v*) and RP-HPLC (30 % - 70 % $CH_3CN$ in $H_2O$, 20 min) to isolate **1a** as a colorless solid (13 mg, 87 %).

**$R_f$** = 0.78 (1:1 $CH_3CN$ / $H_2O$, *v/v*).
**$^1$H-NMR** (400 MHz, DMSO-$d_6$, rotameric mixture): δ = 10.97 (s, 1H, N**H**), 10.12 (s, 1H, N**H**), 8.30 (t, *J* = 5.8 Hz, 1H, N**H**), 7.92 – 7.85 (m, 2H), 7.78 (d, *J* = 2.7 Hz, 1H), 7.70 (d, *J* = 7.4 Hz, 1H), 7.68 – 7.60 (m, 2H), 7.50 (t, *J* = 7.6 Hz, 1H), 7.38 – 7.30 (m, 2H), 7.24 – 7.15 (m, 2H), 7.07 (t, *J* = 7.4 Hz, 1H), 7.03 (d, *J* = 8.9 Hz, 2H), 6.94 – 6.87 (m, 3H), 5.11 (dd, *J* = 13.3, 5.1 Hz, 1H), 5.02 (s, 2H), 4.66 (s, 1H, R2), 4.56 (s, 1H, R1), 4.45 (d, *J* = 17.7 Hz, 1H), 4.31 (d, *J* = 17.7 Hz, 1H), 4.23 (s, 1H, R2), 4.22 (s, 1H, R1), 3.72 – 3.63 (m, 2H, R1/R2), 2.94 – 2.83 (m, 2H), 2.73 (t, *J* = 5.8 Hz, 1H), 2.58 – 2.55 (m, 1H), 2.46 – 2.37 (m, 2H), 2.35 – 2.26 (m, 3H), 2.01 – 1.93 (m, 1H), 1.87 – 1.77 (m, 2H) ppm.
**$^{31}$P-NMR** (162 MHz, DMSO-$d_6$, rotameric mixture) δ = -6.44 (R2), -6.50 (R1) ppm.
**$^{13}$C-NMR** (76 MHz, DMSO-$d_6$, rotameric mixture): δ = 173.2, 172.1, 171.1, 168.2, 166.3, 165.2, 161.0, 157.9, 149.1, 144.3, 135.8, 134.5, 132.1, 131.1, 130.2, 129.7, 128.9, 127.3, 122.9, 122.0 (R1/R2), 121.0, 120.7, 120.3, 120.0 (R1 or R2), 119.9 (R1 or R2), 119.0, 117.2, 114.5, 96.2, 77.0, 66.2, 52.0, 47.4, 45.2 (R2), 43.5 (R1), 42.2 (R1 or R2), 39.7 (R1 or R2), 37.4, 34.4, 28.3, 26.9, 24.6, 22.5, 18.7 ppm.
**HRMS** (ESI, neg): *m/z* $[M-H]^-$ calculated for $[C_{50}H_{46}N_5O_{16}P_2]^-$: 1034.2420, found: 1034.2420
**IR** (KBr): ṽ = 3442 (s), 1673 (s), 1542 (w), 1508 (m), 1487 (m), 1420 (s), 1372 (w), 1309 (w), 1210 (s), 1142 (w), 1081 (w), 1024 (w), 925 (w), 846 (w), 753 (w) $cm^{-1}$.
**UV / Vis** (MeOH): $\lambda_{max}$ = 280, 202 nm.

**2-(2-(4-((3-((8-(2-(2,6-Dioxopiperidin-3-yl)-1-oxoisoindolin-4-yl)oct-7-ynamido)methyl)-4-phenoxyphenyl)carbamoyl)phenoxy)acetyl)-1,2,3,4-tetrahydroisoquinoline-6,7-diyl bis(dihydrogen phosphate) (1b)**

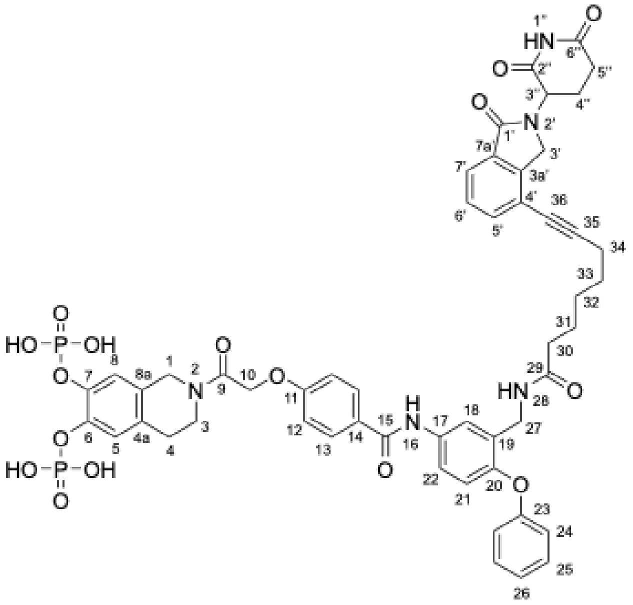

Synthesis of **1b** was carried out by the procedure described in the literature.[14] In brief, **20b** (80 mg, 0.068 mmol, 1.0 eq.) was dissolved in dry DCM (5.6 mL) and cooled to 0 °C. A solution of TMSI in DCM (1 M, 544 µL, 0.544 mmol, 8.0 eq.) was added dropwise and the mixture was left to stir for 20 min at 0 °C. After completion of the reaction indicated by TLC, all volatiles were removed *in vacuo* under cooling. The residue was suspended in 1:1:0.03 $CH_3CN$ / $H_2O$ / TFA (800 µL / 800 µL / 48 µL), stirred for 10 min and purified by reversed-phase column chromatography (1:1 $CH_3CN$ / $H_2O$) to yield **1b** as a yellow solid (87 mg, quant.). The product was further purified by RP-HPLC (30 % - 70 % $CH_3CN$ in $H_2O$, 20 min) to isolate **1b** as a colorless solid (37 mg, 51 %).

**R$_f$** = 0.32 (70 % $H_2O$ / $CH_3CN$, *v/v*).

**$^1$H-NMR** (400 MHz, DMSO-d$_6$, rotameric mixture, recorded at 5 °C): δ = 11.04 (s, 1H, **H-1“**), 10.21 – 10.14 (m, 1H, **H-16**), 8.31 (t, *J* = 5.9 Hz, 1H, **H-28**), 7.89 (d, *J* = 8.7 Hz, 2H, **H-13**), 7.80 – 7.76 (m, 1H, **H-18**), 7.69 (d, *J* = 7.5 Hz, 1H, **H-7‘**), 7.66 – 7.61 (m, 1H, **H-22**), 7.60 (d, *J* = 7.6 Hz, 1H, **H-5‘**), 7.48 (t, *J* = 7.6 Hz, 1H, **H-6‘**), 7.33 (t, *J* = 7.9 Hz, 2H, **H-25**), 7.18 – 7.10 (m, 2H, **H-5,8**), 7.08 – 7.04 (m, 1H, **H-26**), 7.02 (d, *J* = 9.0 Hz, 2H, **H-12**), 6.93 – 6.86 (m, 3H, **H-21,24**), 5.12 (dd, *J* = 13.3, 5.2 Hz, 1H, **H-3“**), 5.01 (s, 1H, R1, **H-10**), 4.99 (s, 1H, R2, **H-10**), 4.63 (s, 1H, R2, **H-1**), 4.54 (s, 1H, R1, **H-1**), 4.42 (d, *J* = 17.8 Hz, 1H, **H-3‘**), 4.27 (d, *J* = 17.8 Hz, 1H, **H-3‘**), 4.20 (s, 2H, **H-27**), 3.72 – 3.62 (m, 2H, R1/R2, **H-3**), 2.94 – 2.81 (m, 2H, R1, **H-5“,4**), 2.75 – 2.65 (m, 1H, R2, **H-4**), 2.59 – 2.56 (m, 1H, **H-5“**), 2.44 (t, *J* = 7.2 Hz, 3H, **H-**

**34,4“**), 2.13 (t, *J* = 7.3 Hz, 2H, **H-30**), 2.02 – 1.93 (m, 1H, **H-4“**), 1.60 – 1.49 (m, 4H, **H-31,33**), 1.45 – 1.31 (m, 2H, **H-32**) ppm.

**$^{31}$P-NMR** (162 MHz, DMSO-$d_6$, rotameric mixture, recorded at 5 °C): δ = -6.35 (R2), -6.39 (R1) ppm.

**$^{13}$C-NMR** (101 MHz, DMSO-$d_6$, rotameric mixture, recorded at 5 °C): δ = 172.7 (R2, **C-6”**), 172.6 (R1, **C-6”**), 172.1 (R2, **C-29**), 172.0 (R1, **C-29**), 170.8 (R2, **C-2”**), 170.7 (R1, **C-2”**), 167.5 (**C-1’**), 165.74 (R2, **C-9**), 165.67 (R1, **C-9**), 164.6 (R2, **C-15**), 164.5 (R1, **C-15**), 160.42 (R2, **C-11**), 160.39 (R1, **C-11**), 157.4 (**C-23**), 148.4 (**C-20**), 143.4 (**C-3a’**), 141.3 – 140.7 (m, **C-6,7**), 135.4 (R2, **C-17**), 135.3 (R1, **C-17**), 133.8 (**C-5’**), 131.6 (**C-7a’**), 130.7 (**C-19**), 130.4 (R2, **C-4a**), 130.3 (R1, **C-4a**), 129.6 (**C-25**), 129.11 (R1, **C-13**), 129.06 (R2, **C-13**), 128.8 (R2, **C-8a**), 128.5 (R1, **C-8a**), 128.3 (**C-6’**), 126.8 (**C-14**), 122.3 (**C-26,7’**), 121.5 (**C-5**), 120.2 (**C-18**), 119.9 (**C-22**), 119.7 (**C-21,8**), 119.5 (**C-8**), 118.5 (**C-4’**), 116.6 (**C-24**), 113.91 (R2, **C-12**), 113.85 (R1, **C-12**), 96.0 (**C-35**), 76.0 (**C-36**), 65.4 (**C-10**), 51.3 (**C-3”**), 46.6 (**C-3’**), 44.3 (R2, **C-1**), 42.7 (R1, **C-1**), 41.2 (**C-3**), 38.6 (**C-3**), 36.8 (R2, **C-27**), 36.7 (R1, **C-27**), 34.91 (R2, **C-30**), 34.86 (R1, **C-30**), 30.8 (**C-5”**), 27.7 (**C-32,4**), 27.6 (**C-31**), 27.0 (**C-4**), 24.5 (**C-33**), 22.0 (**C-4”**), 18.3 (**C-34**) ppm.

**HRMS** (ESI, neg): *m/z* $[M-H]^-$ calculated for $[C_{52}H_{50}N_5O_{16}P_2]^-$: 1062.2733, found: 1062.2756.

**IR** (KBr): $\tilde{v}$ = 3437 (br), 2930 (w), 2226 (w), 1651 (br), 1607 (s), 1544 (w), 1507 (s), 1488 (s), 1384 (m), 1216 (s), 960 (w), 847 (w), 752 (w) $cm^{-1}$.

**UV / Vis** ($H_2O$): $\lambda_{max}$ = 280, 258 nm.

**mp**: 179 - 182°C (decomposition).

**2-(2-(4-((3-((11-(2-(2,6-Dioxopiperidin-3-yl)-1-oxoisoindolin-4-yl)undec-10-ynamido) methyl)-4-phenoxyphenyl)carbamoyl)phenoxy)acetyl)-1,2,3,4-tetrahydroisoquinoline-6,7-diyl bis(dihydrogen phosphate) (1c)**

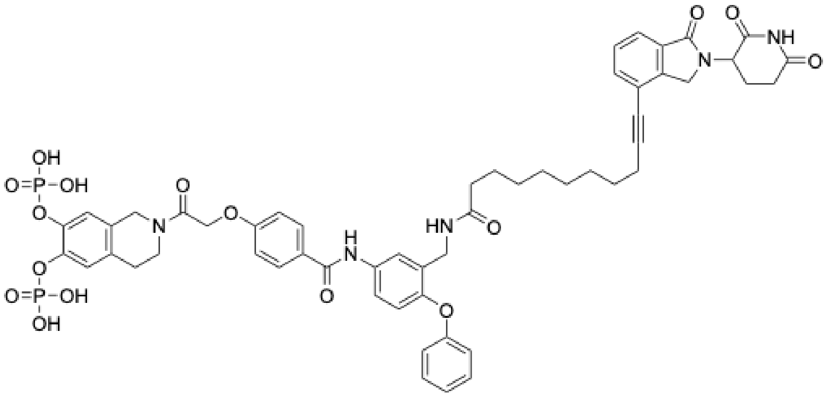

Synthesis of **1c** was carried out essentially as described in the literature.[7] In brief, **20c** (64 mg, 0.053 mmol, 1.0 eq.) was dissolved in dry DCM (2 mL) and cooled to 0 °C. A solution of TMSI in DCM (1M, 420 µL, 0.420 mmol, 8.0 eq.) and BSTFA (170 µL, 0.630 mmol, 12 eq.) were added and the mixture was left to stir for 20 min. After completion of the reaction indicated by TLC, all volatiles were removed *in vacuo*. The crude product was purified by reversed-phase column chromatography (1:1 $CH_3CN$ / $H_2O$, *v/v*) and RP-HPLC (30 % - 70 % $CH_3CN$ in $H_2O$, 20 min) to isolate **1c** as a colorless solid (48 mg, 81 %).

**$R_f$** = 0.73 (1:1 $CH_3CN$ / $H_2O$, *v/v*).
**$^1$H-NMR** (400 MHz, DMSO-$d_6$, rotameric mixture): δ = 7.91 – 7.86 (m, 2H), 7.73 (d, *J* = 2.6 Hz, 1H), 7.69 (dd, *J* = 7.7, 1.1 Hz, 1H), 7.62 – 7.57 (m, 2H), 7.50 (t, *J* = 7.6 Hz, 1H), 7.37 – 7.30 (m, 2H), 7.18 – 7.11 (m, 2H), 7.08 – 7.05 (m, 1H), 7.05 – 7.00 (m, 2H), 6.93 – 6.85 (m, 3H), 5.08 (dd, *J* = 13.3, 5.1 Hz, 1H), 4.98 (s, 2H), 4.62 (s, 1H, R2), 4.53 (s, 1H, R1), 4.42 (d, *J* = 17.5 Hz, 1H), 4.28 (d, *J* = 17.8 Hz, 1H), 4.19 (s, 2H), 3.56 (1H, R1/R2), 3.58 (1H, R1/R2), 2.91 – 2.80 (m, 2H), 2.71 (s, 1H), 2.63 – 2.54 (m, 1H), 2.40 (t, *J* = 7.1 Hz, 3H), 2.12 – 2.05 (m, 2H), 2.02 (m, 1H), 1.55 – 1.43 (m, 4H), 1.39 – 1.30 (m, 2H), 1.27 – 1.18 (m, 6H) ppm.
**$^{31}$P-NMR** (162 MHz, DMSO-$d_6$, rotameric mixture) δ = -6.39 (R2), -6.45 (R1) ppm.
**$^{13}$C-NMR** (101 MHz, DMSO-$d_6$, rotameric mixture): δ = 172.8, 172.6, 170.8, 167.9, 166.0, 164.8, 160.6, 157.5, 148.9, 143.6, 141.6 – 140.3 (m), 135.3, 134.0, 131.7, 130.8, 130.7 (R2), 130.5 (R1), 129.8, 129.3, 129.1 (R1), 128.8 (R2), 128.6, 127.1, 122.6, 122.5, 121.4 (R1/R2), 120.7, 120.3, 119.7, 119.3 (R2), 119.4 (R1), 118.8, 116.9, 114.2, 96.5, 76.2, 65.7, 51.6, 47.0, 44.6 (R2), 42.8 (R1), 41.4, 38.9, 37.0, 35.2, 31.0, 28.6, 28.5, 28.2, 28.1, 27.84, 27.77 (R1), 27.0 (R2), 25.1, 22.2, 18.6 ppm.
**HRMS** (ESI, neg): *m/z* $[M-H]^-$ calculated for $[C_{55}H_{56}N_5O_{16}P_2]^-$: 1104.3203, found: 1104.3203.

**IR** (KBr): ṽ = 3440 (s), 1671 (s), 1546 (w), 1508 (m), 1483 (s), 1420 (s), 1385 (w), 1344 (w), 1309 (w), 1207 (s), 1156 (w), 1052 (w), 1030 (w), 925 (w), 852 (w), 733 (w), 678 (w) $cm^{-1}$.
**UV / Vis** (MeOH): $\lambda_{max}$ = 279, 235 nm.

**Prodrug 21**

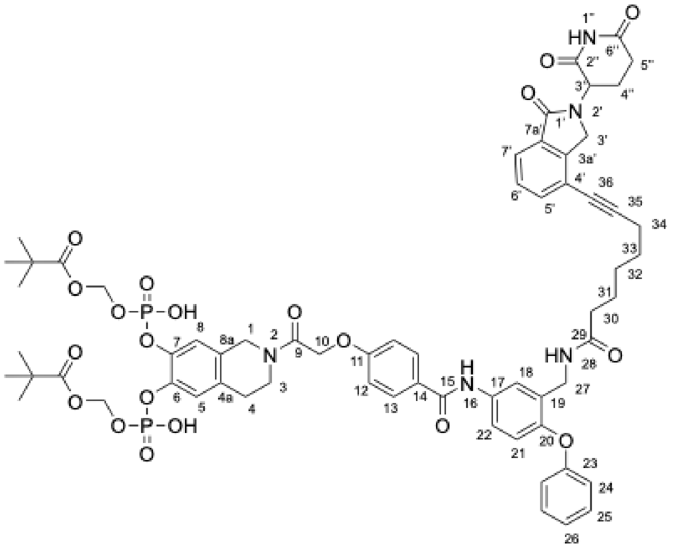

DIPEA (249 µL, 1.43 mmol, 10 eq.) and POM-I (214 µL, 1.43 mmol, 10 eq.) were added to a suspension of **1b** (152 mg, 0.143 mmol, 1.0 eq.) in $CH_3CN$ (1.5 mL). The reaction mixture was stirred overnight at rt. All volatiles were removed *in vacuo* and the residue was purified by column chromatography (5-20 % MeOH in DCM), followed by reversed-phase column chromatography (80 % - 60 % $H_2O$ / $CH_3CN$, *v/v*) to isolate **21** as a colorless solid (26 mg, 14 %).

$R_f$ = 0.42 (60 % $H_2O$ / $CH_3CN$, *v/v*).
**$^1$H-NMR** (400 MHz, DMSO-d$_6$, rotameric mixture): δ = 10.23 (s, 1H, **H-1''**), 10.14 (s, 1H, **H-16**), 8.28 – 8.18 (m, 1H, R1/R2, **H-28**), 7.93 – 7.87 (m, 2H, **H-13**), 7.83 – 7.75 (m, 1H, **H-18**), 7.69 (d, *J* = 7.6 Hz, 1H, **H-7'**), 7.66 – 7.62 (m, 1H, **H-22**), 7.62 – 7.58 (m, 1H, **H-5'**), 7.49 (t, *J* = 7.6 Hz, 1H, **H-6'**), 7.38 – 7.31 (m, 2H, **H-25**), 7.16 – 7.07 (m, 2H, R1/R2, **H-5,8**), 7.07 – 7.04 (m, 1H, **H-26**), 7.04 – 6.99 (m, 1H, R1, **H-12**), 6.96 (d, *J* = 8.2 Hz, 1H, R2, **H-12**), 6.94 – 6.86 (m, 3H, **H-21,24**), 5.47 (d, $^3J_{P-H}$ = 11.6 Hz, 4H, -OC**H$_2$**OC(O)C($CH_3$)$_3$), 5.11 (dd, *J* = 13.4, 5.1 Hz, 1H, **H-3''**), 5.03 – 4.95 (m, 2H, R1/R2, **H-10**), 4.56 (s, 1H, R2, **H-1**), 4.46 (s, 1H, R1, **H-1**), 4.45 (d, *J* = 17.5 Hz, 1H, **H-3'**), 4.29 (d, *J* = 17.5 Hz, 1H, **H-3'**), 4.25 – 4.17 (m, 2H, R1/R2, **H27**), 3.69 – 3.60 (m, 2H, R1/R2, **H-3**), 2.93 – 2.82 (m, 1H, **H-5''**), 2.81 – 2.73 (m, 1H, R1, **H-4**), 2.65 – 2.57 (m, 2H, R2, **H-5'',4**), 2.46 – 2.39 (m, 3H, **H-34,4''**), 2.19 – 2.11 (m, 2H, **H-30**), 2.03 – 1.94 (m, 1H, **H-4''**), 1.62 – 1.51 (m, 4H, **H-31,33**), 1.47 – 1.37 (m, 2H, **H-32**), 1.12 – 1.08 (m, 18H, -OCH$_2$OC(O)C**($CH_3$)$_3$**) ppm.

$^{31}$**P-NMR** (162 MHz, DMSO-d$_6$, rotameric mixture): δ = -8.70 ppm.
$^{13}$**C-NMR** (101 MHz, DMSO-d$_6$, rotameric mixture): δ = 176.6 (-OCH$_2$O**C**(O)C(CH$_3$)$_3$), 172.8 (**C-6"**), 172.1 (**C-29**), 170.9 (**C-2"**), 167.6 (**C-1'**), 166.1 (R2, **C-9**), 165.8 (R1, **C-9**), 164.7 (**C-15**), 160.6 (R1, **C-11**), 160.5 (R2, **C-11**), 157.7 (**C-23**), 148.7 (**C-20**), 143.6 (**C-3a**), 143.3 – 142.7 (m, **C-6,7**), 135.7 (**C-17**), 134.0 (**C-5'**), 131.9 (**C-7a'**), 130.9 (**C-19**), 129.8 (**C-25**), 129.3 (**C-13**), 128.5 (**C-6'**), 127.2 (**C-14**), 126.1 (R1/R2, **C-4a/8a**), 125.9 (R1/R2, **C-4a/8a**), 122.5 (**C-7',26**), 120.72 (**C-8**), 120.67 (**C-18**), 120.3 (**C-22**), 119.8 (**C-21**), 118.8 (R1/R2, **C-5,4'**), 118.3 (R1/R2, **C-5**), 116.9 (**C-24**), 114.1 (**C-12**), 96.3 (**C-35**), 83.2 (-O**C**H$_2$OC(O)C(CH$_3$)$_3$), 76.3 (**C-36**), 65.9 (**C-10**), 51.6 (**C-3"**), 47.0 (**C-3'**), 45.1 (R2, **C-1**), 43.2 (R1, **C-1**), 41.8 (**C-3**), 39.4 (**C-3**), 38.0 (-OCH$_2$OC(O)**C**(CH$_3$)$_3$), 37.0 (**C-27**), 35.1 (**C-30**), 31.1 (**C-5"**), 28.1 (**C-4**), 27.9 (**C-32**), 27.8 (**C-31**), 27.1 (**C-4**), 26.6 (-OCH$_2$OC(O)C(**C**H$_3$)$_3$), 24.7 (**C-33**), 22.2 (**C-4"**), 18.6 (**C-34**) ppm.
**HRMS** (ESI, neg): *m/z* [M-H]$^-$ calculated for [$C_{64}H_{70}N_5O_{20}P_2$]$^-$: 1290.4095, found: 1290.4079.
**mp**: 230°C (decomposition).

**(5-(4-(2-(6,7-Dihydroxy-3,4-dihydroisoquinolin-2(1*H*)-yl)-2-oxoethoxy)benzamido)-2-phenoxyphenyl)methanaminium chloride (S1a)**

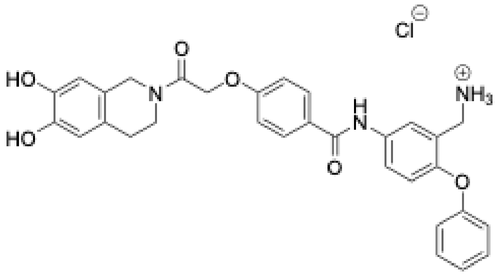

A Schlenk flask containing HCl in dioxane (4 M, 9 mL, 36 mmol, 60 eq.) was cooled to 0 °C and **17** (378 mg, 0.591 mmol, 1.0 eq.) was added. The ice bath was removed after 5 min and the mixture was left to stir for 40 min. Afterwards, the volatiles were removed *in vacuo* to yield **S1a** as a yellow foam (297 mg, 87 %).

$^1$**H-NMR** (400 MHz, CD$_3$OD, rotameric mixture): δ = 7.98 – 7.90 (m, 3H), 7.56 – 7.51 (m, 1H), 7.46 – 7.39 (m, 2H), 7.23 – 7.17 (m, 1H), 7.14 – 7.06 (m, 4H), 6.91 – 6.86 (m, 1H), 6.63 – 6.54 (m, 2H), 4.99 (s, 2H), 4.60 (s, 1H, R2), 4.55 (s, 1H, R1), 4.20 (s, 2H), 3.80 – 3.70 (m, 2H), 2.80 (t, *J* = 5.9 Hz, 1H, R1), 2.71 (t, *J* = 6.0 Hz, 1H, R2) ppm.
$^{13}$**C-NMR** (76 MHz, CD$_3$OD, rotameric mixture): δ = 168.81 (R2), 168.77 (R1), 168.3, 162.7, 157.7, 153.6, 145.6 (R2), 145.4 (R1), 145.3 (R1), 145.2 (R2), 135.7, 131.2, 130.60 (R1), 130.55 (R2), 128.62 (R1), 128.55 (R2), 126.9 (R2), 126.4 (R1), 125.4, 125.2, 125.0, 124.9, 124.7 (R1), 124.4 (R2), 120.2, 119.3, 116.1 (R2), 116.0 (R1), 115.7 (R2), 115.7 (R1), 114.0

(R1), 113.9 (R2), 67.6 (R1), 67.5 (R2), 46.9 (R2), 45.1 (R1), 44.1 (R1), 41.8 (R2), 39.8, 29.5 (R1), 28.5 (R2) ppm.

**HRMS** (ESI, pos): *m/z* $[M+H]^+$ calculated for $[C_{31}H_{30}N_3O_6]^+$: 540.2129, found: 540.2152.

**IR** (KBr): ṽ = 3433 (br), 2923 (w), 1644 (s), 1606 (s), 1530 (w), 1505 (m), 1488 (s), 1425 (w), 1374 (w), 1222 (s), 1181 (m), 760 (w), 693 (w) $cm^{-1}$.

**UV / Vis** (MeOH): $\lambda_{max}$ = 278, 210 nm.

**mp**: 132°C (decomposition).

**4-(2-(6,7-Dihydroxy-3,4-dihydroisoquinolin-2(1*H*)-yl)-2-oxoethoxy)-*N*-(3-((8-(2-(2,6-dioxopiperidin-3-yl)-1-oxoisoindolin-4-yl)oct-7-ynamido)methyl)-4-phenoxyphenyl) benzamide (S1b)**

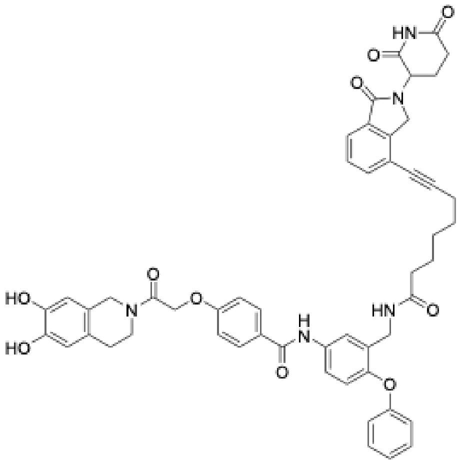

To a solution of **S1a** (130 mg, 0.226 mmol, 1.0 eq.) and **2b** (86 mg, 0.226 mmol, 1.0 eq.) in dry DMF (2.5 mL) were added DIPEA (196 µL, 1.13 mmol, 5.0 eq.) and HATU (95 mg, 0.249 mmol, 1.1 eq.). The resulting suspension was stirred overnight at rt, followed by the addition of 5 % LiCl-solution (80 mL) and extraction with EtOAc (3 x 80 mL). The combined organic phases were washed with 5 % LiCl-solution (1 x 100 mL) and brine (1 x 100 mL), dried over $Na_2SO_4$, filtered and concentrated *in vacuo*. The crude product was purified by column chromatography (5 - 7 % MeOH in DCM, *v/v*) to yield **S1b** as an off-white solid (93 mg, 46 %).

$\mathbf{R_f}$ = 0.15 (5 % MeOH in DCM, *v/v*).

**$^1$H-NMR** (400 MHz, $CD_3OD$, rotameric mixture): δ = 7.94 – 7.85 (m, 2H), 7.77 – 7.74 (m, 1H), 7.70 (d, *J* = 7.5 Hz, 1H), 7.54 (d, *J* = 7.6 Hz, 1H), 7.48 – 7.41 (m, 2H), 7.36 – 7.28 (m, 2H), 7.11 – 7.00 (m, 3H), 6.94 (d, *J* = 8.1 Hz, 2H), 6.84 (dd, *J* = 8.7, 1.5 Hz, 1H), 6.63 – 6.54 (m, 2H), 5.11 (dd, *J* = 13.3, 5.2 Hz, 1H), 4.93 (s, 2H), 4.56 (s, 1H, R2), 4.54 (s, 1H, R1), 4.45 – 4.37 (m, 4H), 3.78 – 3.68 (m, 2H), 2.91 – 2.82 (m, 1H), 2.81 – 2.75 (m, 2H), 2.75 – 2.67 (m,

1H), 2.56 – 2.47 (m, 1H), 2.43 (t, *J* = 6.8 Hz, 2H), 2.25 (t, *J* = 7.2 Hz, 2H), 2.18 – 2.10 (m, 1H), 1.73 – 1.57 (m, 4H), 1.56 – 1.47 (m, 2H) ppm.
**$^{13}$C-NMR** (76 MHz, $CD_3OD$, rotameric mixture): δ = 176.1, 174.7, 172.2, 171.1, 168.8, 168.0, 162.5, 159.2, 152.0, 145.6 – 145.2 (m), 145.3, 136.1, 135.8, 132.9, 131.6, 130.9, 130.6 (R1), 130.5 (R2), 129.6, 128.92 (R1), 128.85 (R2), 126.9 (R2), 126.5 (R1), 124.7 (R1), 124.4 (R2), 124.0, 123.6, 123.2, 122.6, 121.0, 120.8, 118.7, 116.1 (R2), 116.0 (R1), 115.7 (R2), 115.6 (R1), 114.0 (R1), 113.9 (R2), 97.3, 77.4, 67.6 (R1), 67.5 (R2), 55.8, 53.8, 46.9 (R1), 45.1 (R2), 44.1 (R1), 43.8 (R2), 41.8, 39.3, 36.9, 32.4, 29.53 (R1), 29.46 (R1), 29.4 (R2), 28.5 (R2), 26.5, 24.0, 20.0 ppm.
**HRMS** (ESI, pos): *m/z* $[M+H]^+$ calculated for $[C_{52}H_{50}N_5O_{10}]^+$: 904.3552, found: 904.3562.
**IR** (KBr): $\tilde{v}$ = 3414 (br), 2930 (w), 2470 (w), 1696 (s), 1639 (s), 1528 (w), 1486 (s), 1455 (s), 1384 (m), 1221 (br), 1174 (s), 847 (s), 752 (m) $cm^{-1}$.
**UV / Vis** (MeOH): $\lambda_{max}$ = 279, 257, 206 nm.
**mp**: 162°C (decomposition).

**2-(2-(4-((3-((8-(2-(2,6-Dioxopiperidin-3-yl)-1-oxoisoindolin-4-yl)oct-7-ynamido)methyl)-4-phenoxyphenyl)carbamoyl)phenoxy)acetyl)-1,2,3,4-tetrahydroisoquinoline-6,7-diyl tetraphenyl bis(phosphate) (S1)**

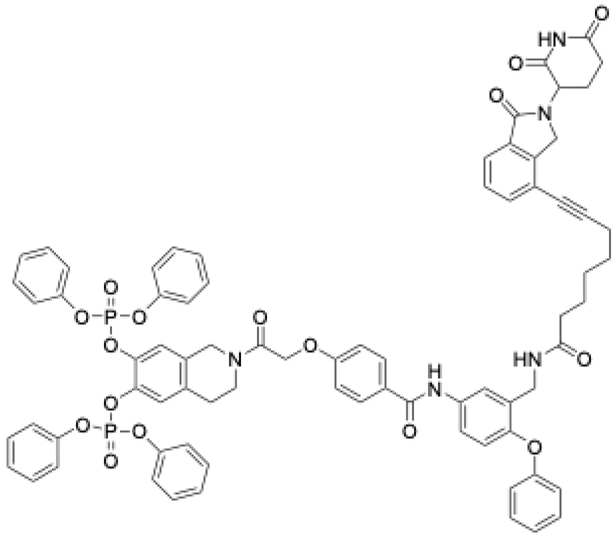

To a solution of **S1b** (60 mg, 0.066 mmol, 1.0 eq.) in dry $CH_3CN$ (3 mL) and DMF (1 mL) were added $CCl_4$ (64 µL, 0.66 mmol, 10 eq.), DIPEA (45 µL, 0.27 mmol, 4.0 eq.) and a catalytic amount of DMAP. The reaction mixture was cooled to 0 °C. Diphenyl phosphite (38 µL, 0.20 mmol, 3.0 eq.) was added and the mixture was left to stir for 1 h. Afterwards, all volatiles were removed *in vacuo* and the crude product was purified by fast column chromatography (5 % MeOH in DCM, *v*/*v*) to yield **S1** as a colorless solid (7 mg, 8 %).

**R$_f$** = 0.33 (5 % MeOH in DCM, *v/v*).

**$^1$H-NMR** (400 MHz, $CD_2Cl_2$, rotameric mixture): δ = 8.69 – 8.47 (m, 1H, N**H**), 8.36 – 7.93 (m, 1H, N**H**), 7.85 (d, *J* = 8.5 Hz, 1H), 7.75 (d, *J* = 8.5 Hz, 1H), 7.72 – 7.66 (m, 1H), 7.62 – 7.55 (m, 1H), 7.52 (d, *J* = 7.6 Hz, 2H), 7.40 (t, *J* = 7.6 Hz, 1H), 7.36 – 7.25 (m, 11H), 7.25 – 7.14 (m, 13H), 7.10 – 7.05 (m, 1H), 6.98 (d, *J* = 8.5 Hz, 1H), 6.96 – 6.91 (m, 2H), 6.89 – 6.79 (m, 2H), 6.12 – 6.03 (m, 1H, N**H**), 5.15 (dd, *J* = 13.2, 5.1 Hz, 1H), 4.82 (s, 2H), 4.65 (s, 2H), 4.43 (d, *J* = 16.9 Hz, 1H), 4.39 – 4.36 (m, 2H), 4.33 (d, *J* = 17.0 Hz, 1H), 3.77 (t, *J* = 6.1 Hz, 1H, R2), 3.72 (t, *J* = 5.8 Hz, 1H, R1), 2.90 – 2.85 (m, 1H, R1), 2.81 – 2.75 (m, 2H, R1/R2), 2.74 – 2.67 (m, 1H, R2), 2.43 (t, *J* = 6.7 Hz, 3H), 2.21 – 2.11 (m, 3H), 1.67 – 1.54 (m, 4H), 1.52 – 1.40 (m, 2H) ppm.

**$^{31}$P-NMR** (162 MHz, $CD_2Cl_2$, rotameric mixture): δ = -17.26 (R2), -17.35 (R1) ppm.

**$^{13}$C-NMR** (101 MHz, $CDCl_3$, rotameric mixture): δ = 172.8 (R1), 172.7 (R2), 171.5 (R1), 171.4 (R2), 169.8 (R2), 169.8 (R1), 169.0, 166.5 (R1), 166.4 (R2), 165.3 (R2), 165.1 (R1), 160.6 (R1), 160.0 (R2), 157.8 (R1), 157.7 (R2), 150.6 (R1), 150.4 (R2), 150.3 (R2), 150.2 (R1), 143.82 (R1), 143.78 (R2), 140.0 – 139.7 (m), 134.7 (R2), 134.4 (R1), 134.3 (R1), 134.2 (R2), 132.9 (R2), 132.0 (R1), 131.5, 130.8 (R2), 130.7 (R1), 130.4 (R1), 130.2 (R2), 129.93 (R2), 129.90 (R1), 129.87 (R1), 129.8 (R2), 129.3 (R1), 129.2 (R2), 128.3, 128.0, 125.8 (R2), 125.7 (R1), 123.1, 123.0 (R1), 122.9 (R2), 122.1 (R1), 122.0, 121.4 (R2), 121.2 (R1), 121.1 (R2), 120.10 (R1), 120.05 (R2), 119.7 (R2), 119.5, 119.1 (R1), 117.5 (R1), 117.4 (R2), 114.52 (R1), 114.46 (R2), 96.0, 76.6, 67.7 (R2), 67.6 (R1), 51.9, 47.1, 46.6 (R2), 44.0 (R1), 42.6 (R1), 40.3 (R2), 38.8, 36.6, 31.5, 29.0 (R1), 28.33 (R2), 28.29 (R1), 28.11 (R2), 28.06 (R1), 27.6 (R2), 25.2, 23.3, 19.2 ppm.

**HRMS** (ESI, pos): *m/z* $[M+H]^+$ calculated for $[C_{76}H_{68}N_5O_{16}P_2]^+$: 1368.4131, found: 1368.4129.

**mp**: 96 - 97°C.

**NMR spectra**

$^{1}$H-NMR of compound **1a**

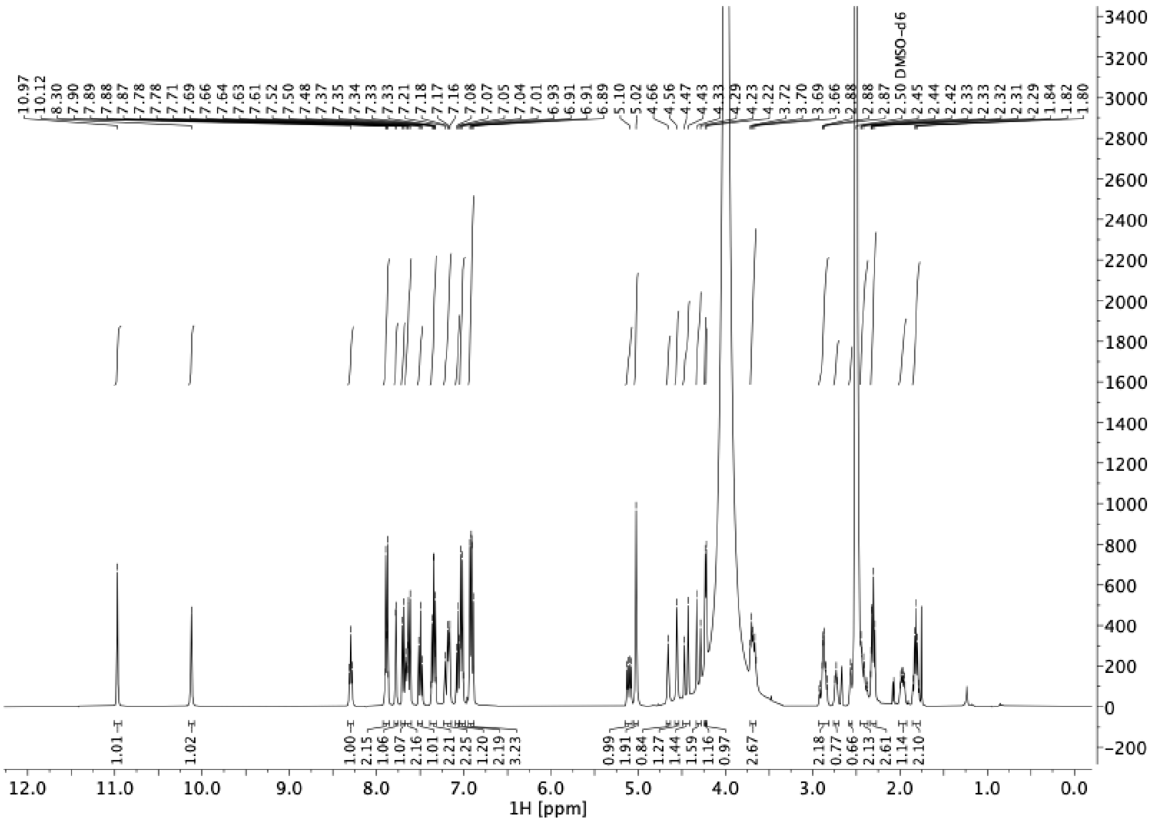


$^{31}$P-NMR of compound **1a**

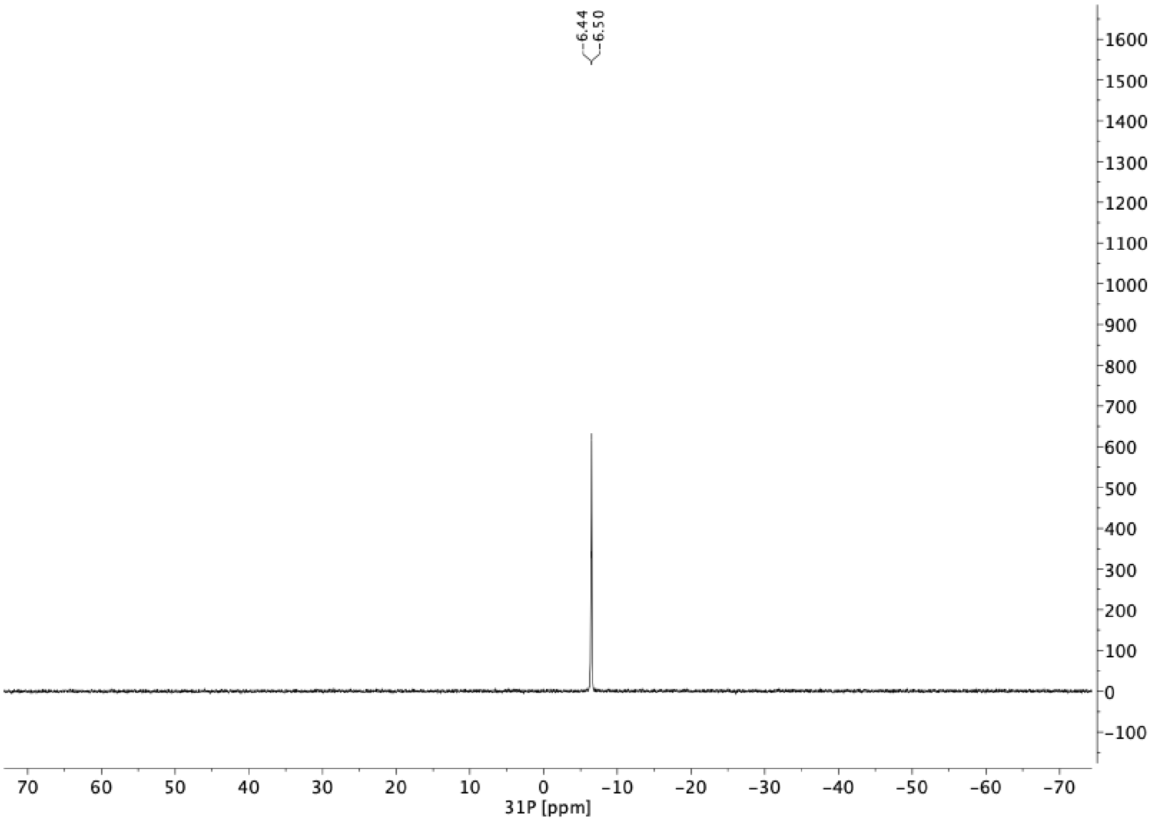

$^{13}$C-NMR of compound **1a**

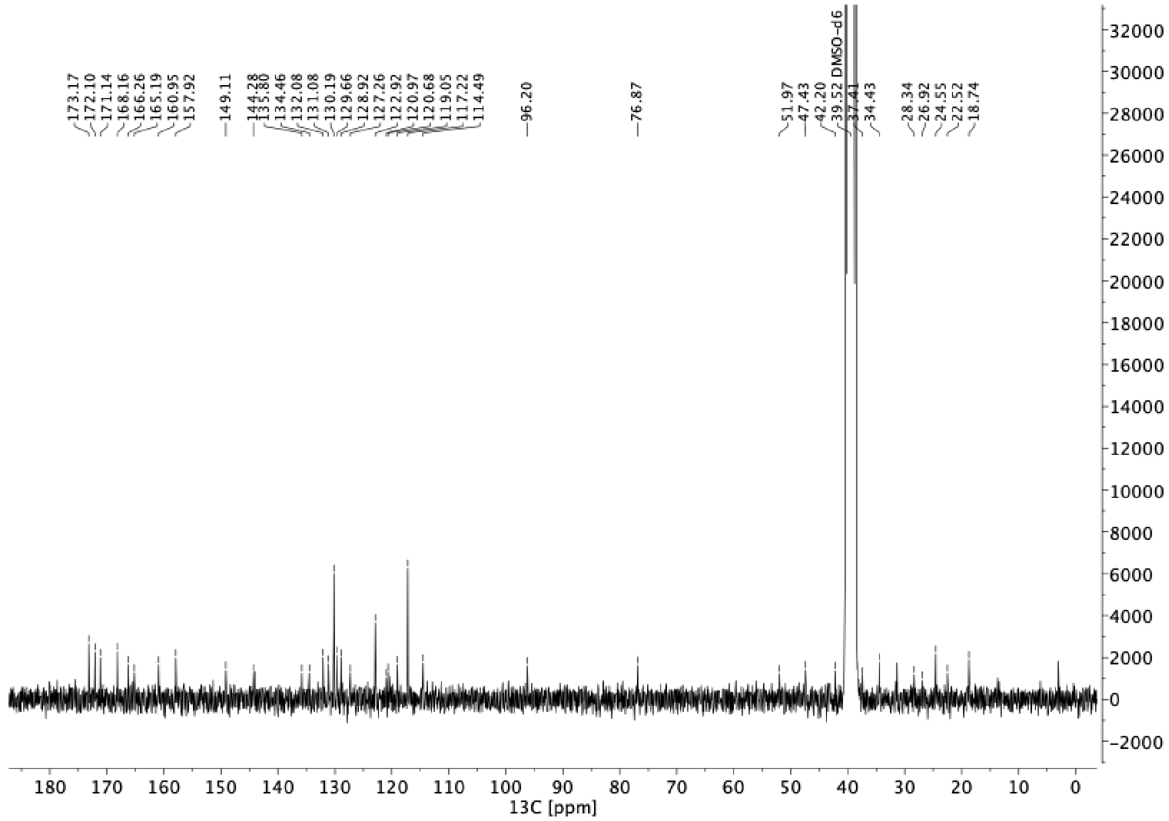

$^{1}$H-NMR of compound **1b**

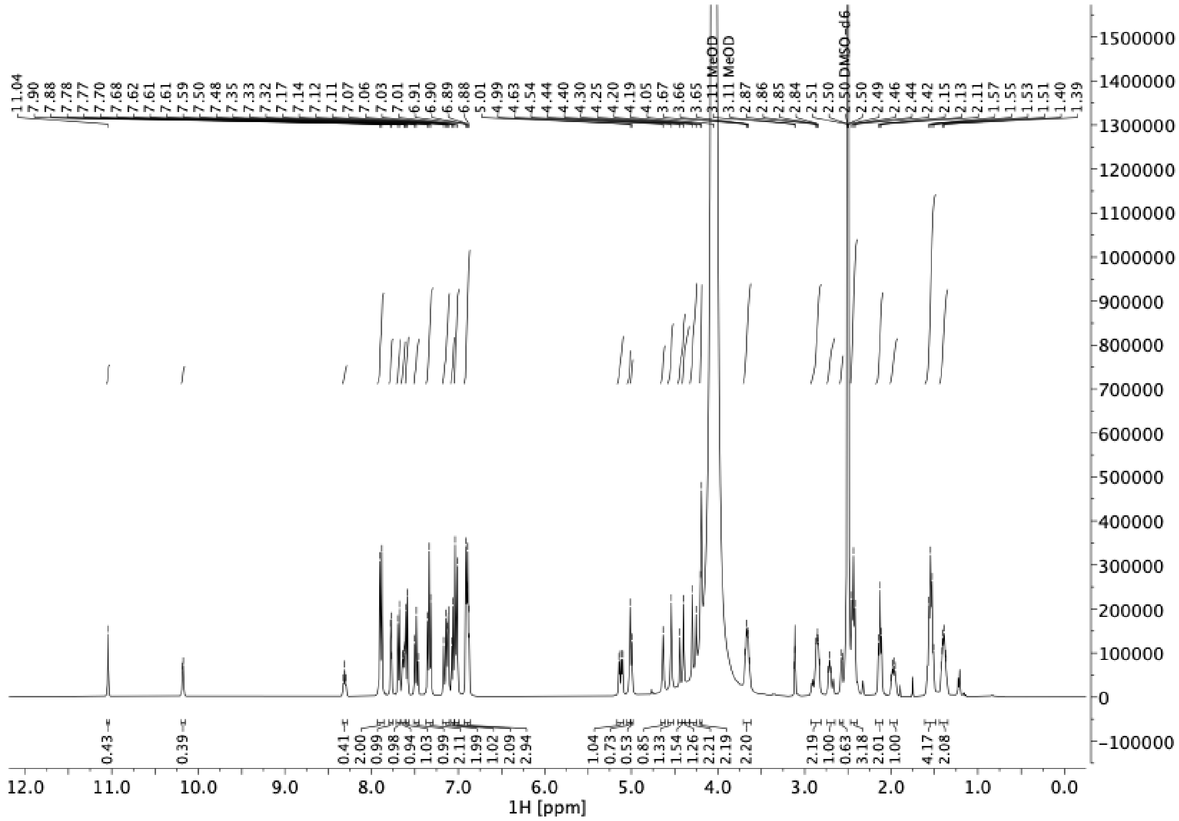

$^{31}$P-NMR of compound **1b**

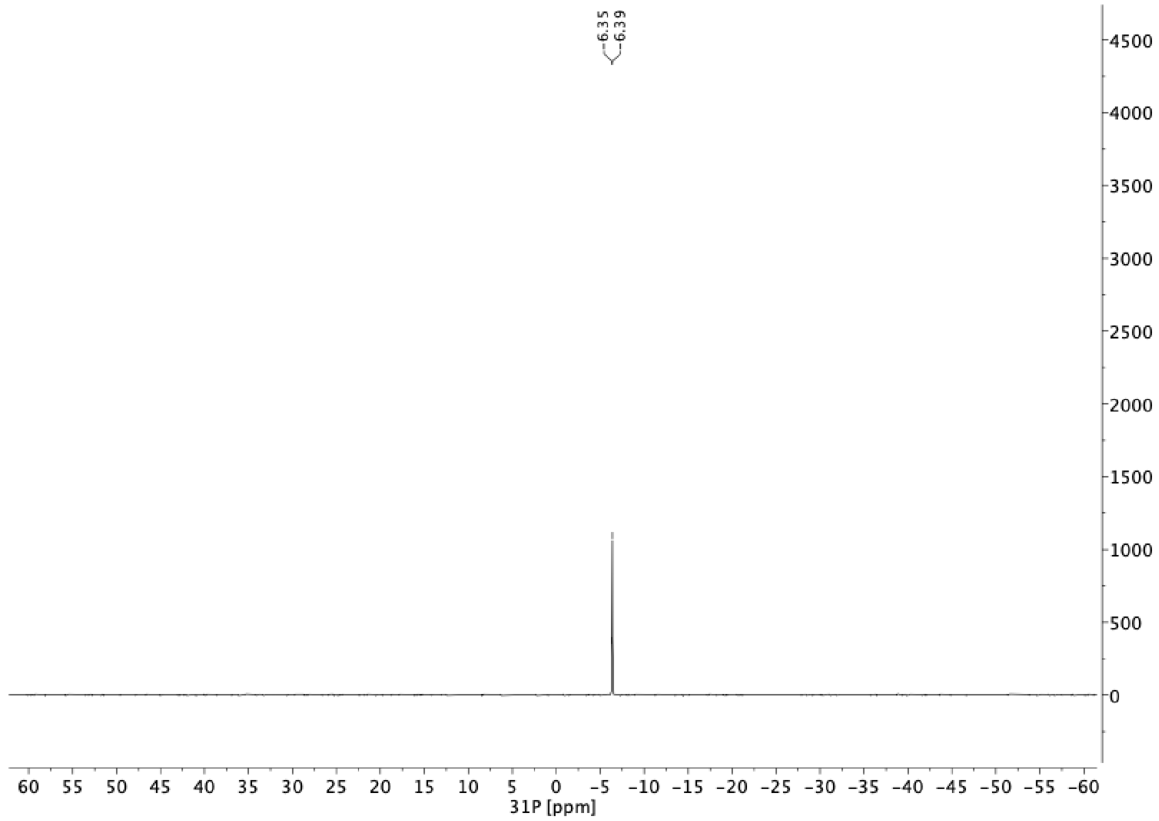


$^{13}$C-NMR of compound **1b**

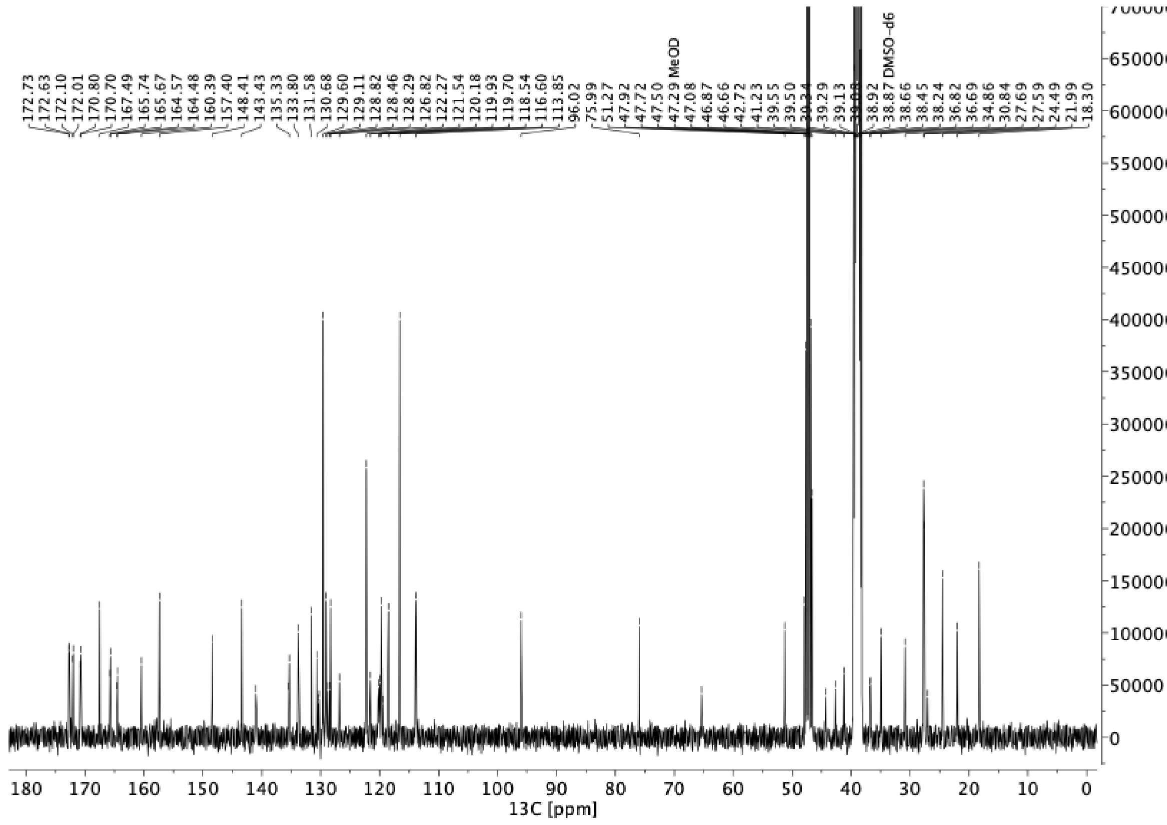

$^{1}$H-NMR of compound **1c**

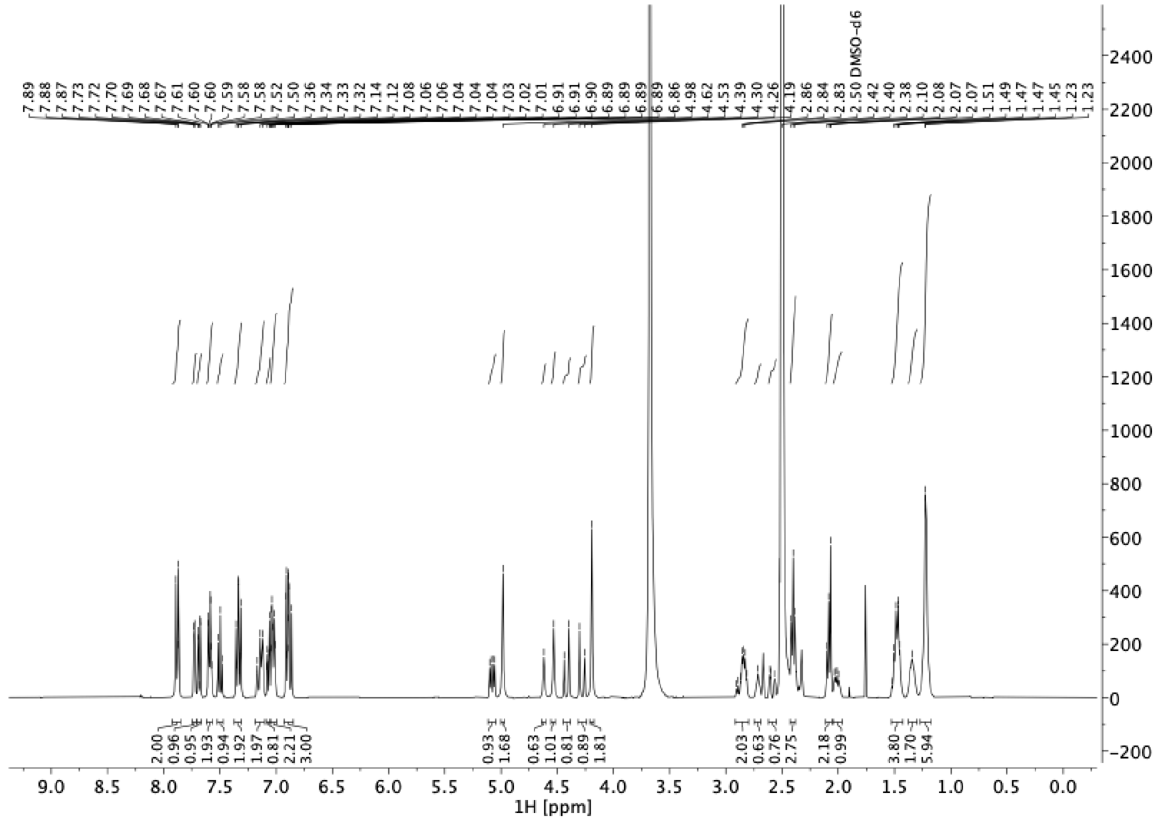

$^{31}$P-NMR of compound **1c**

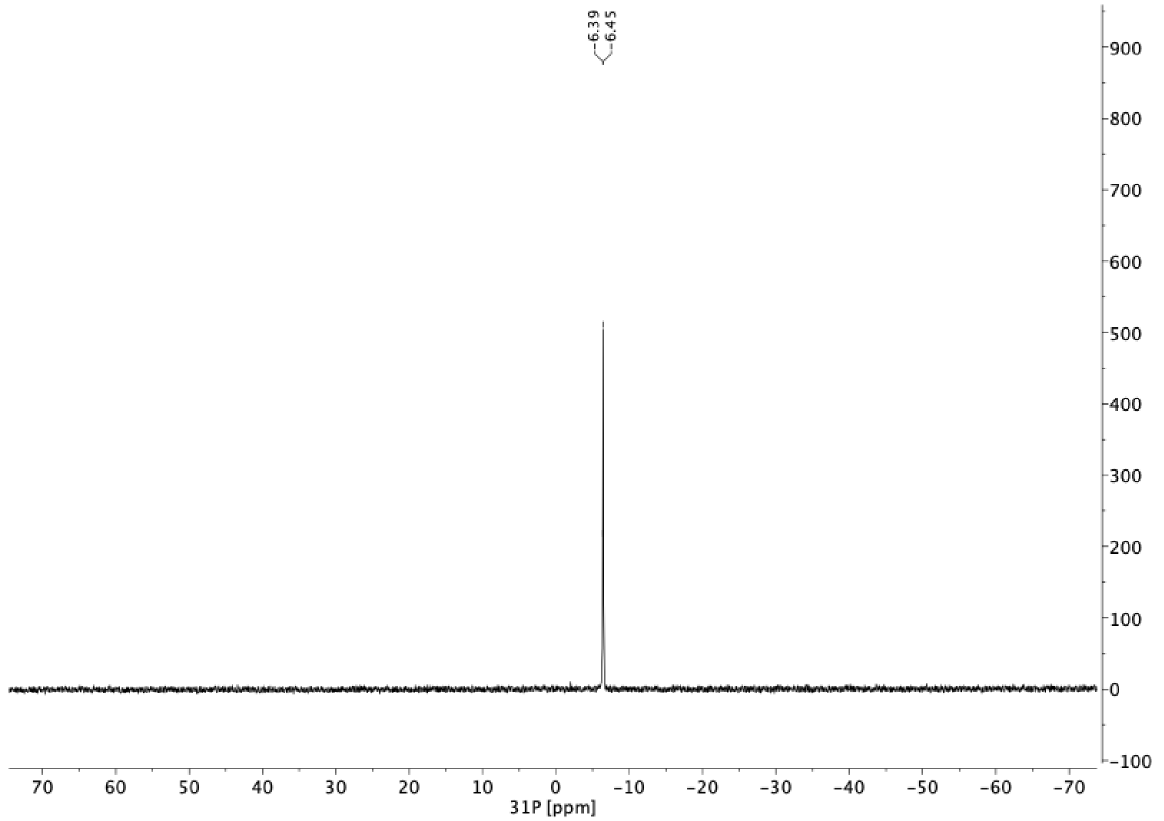

$^{13}$C-NMR of compound **1c**

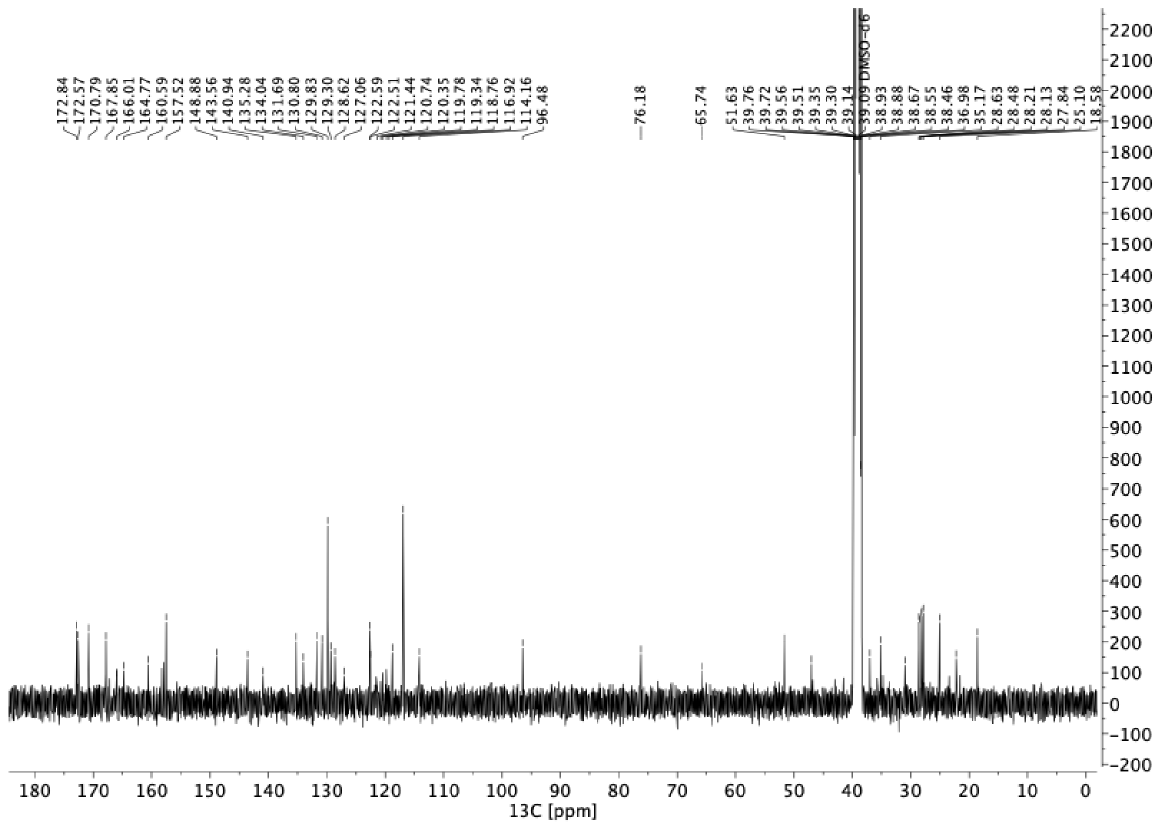

$^{1}$H-NMR of compound **21**

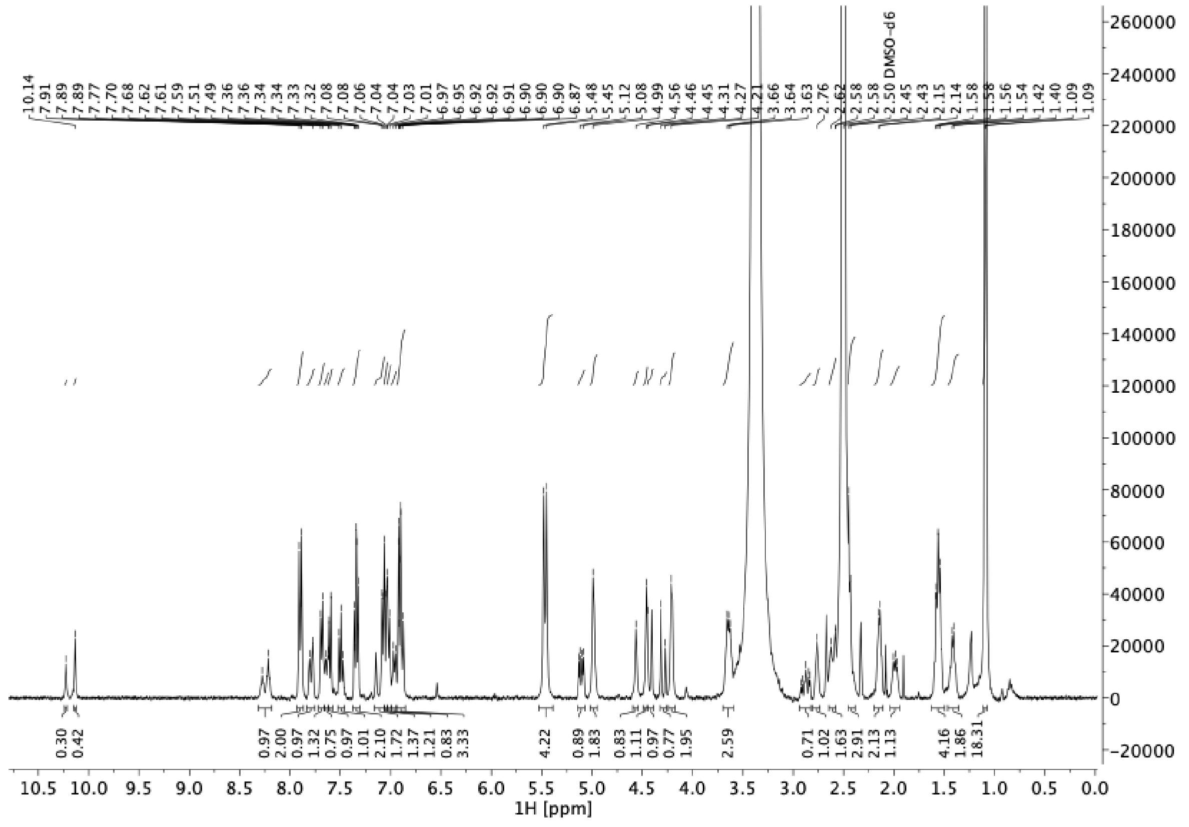

<sup></sup>$^{31}$P-NMR of compound **21**

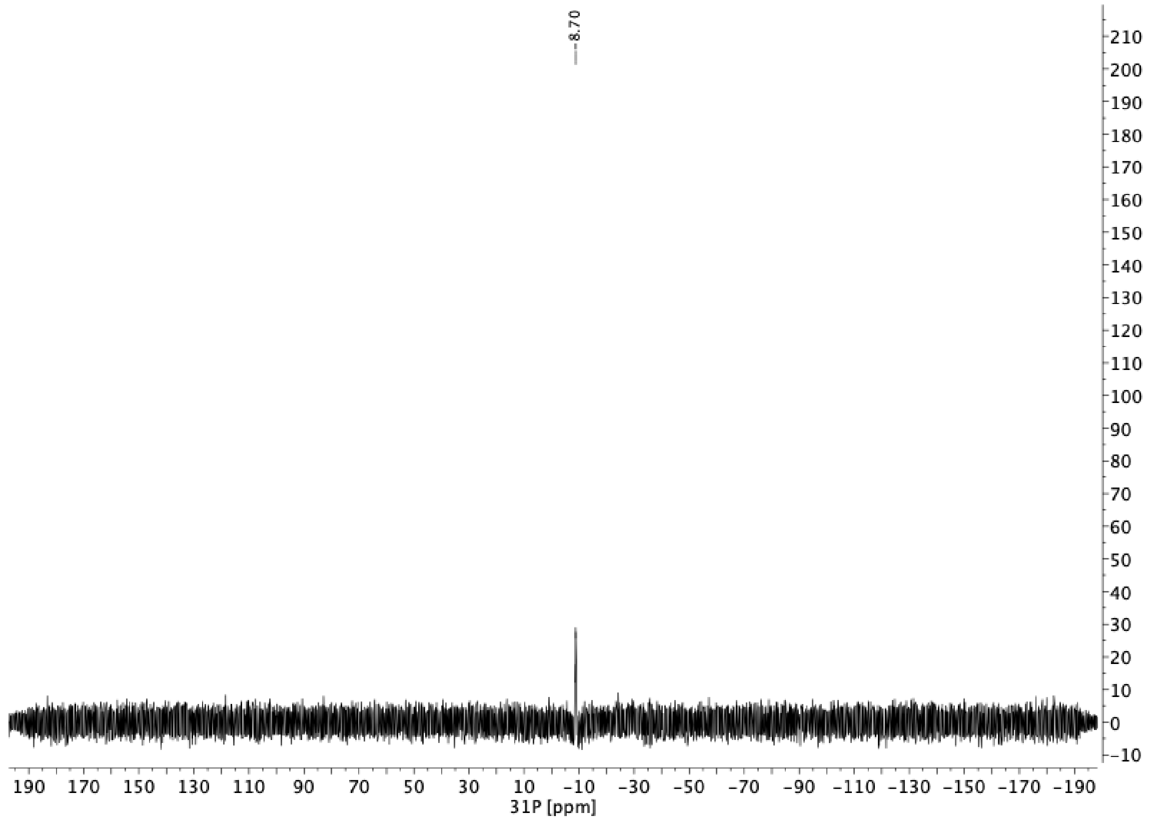


$^{13}$C-NMR of compound **21**

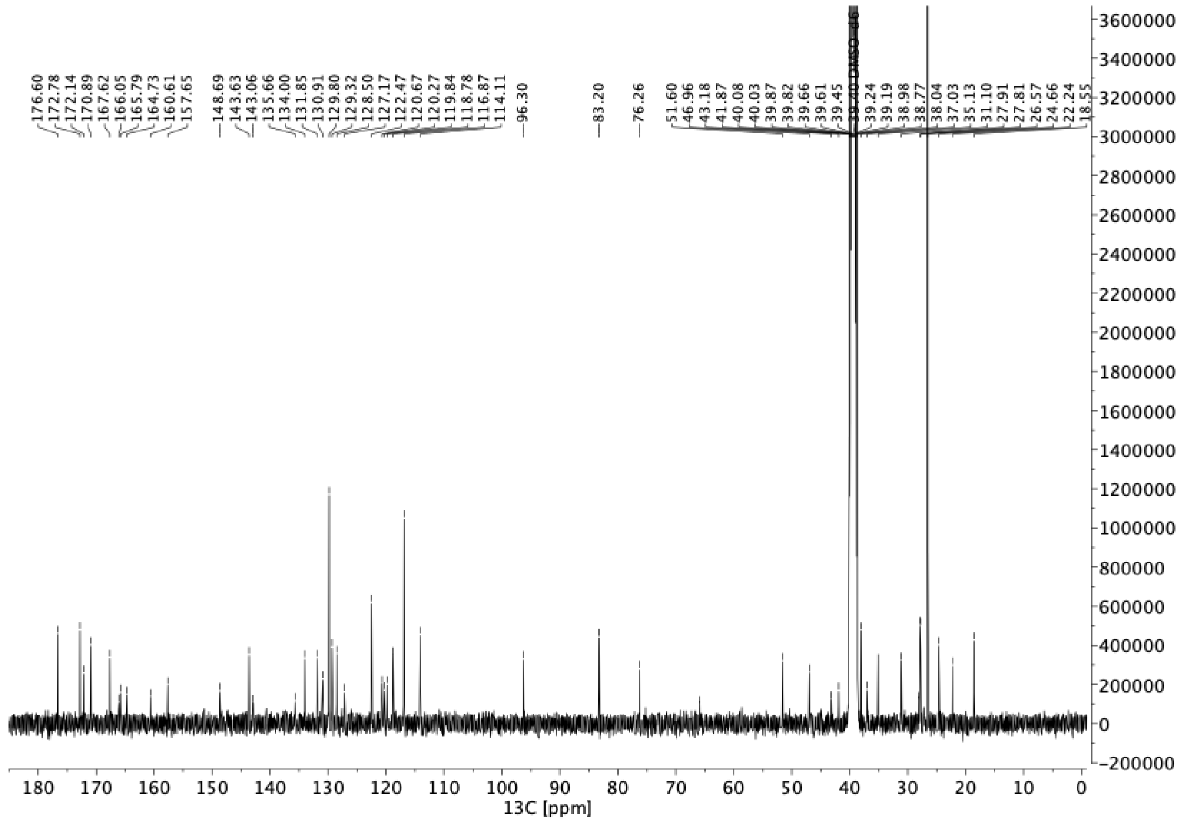

$^{1}$H-NMR of compound **S1**

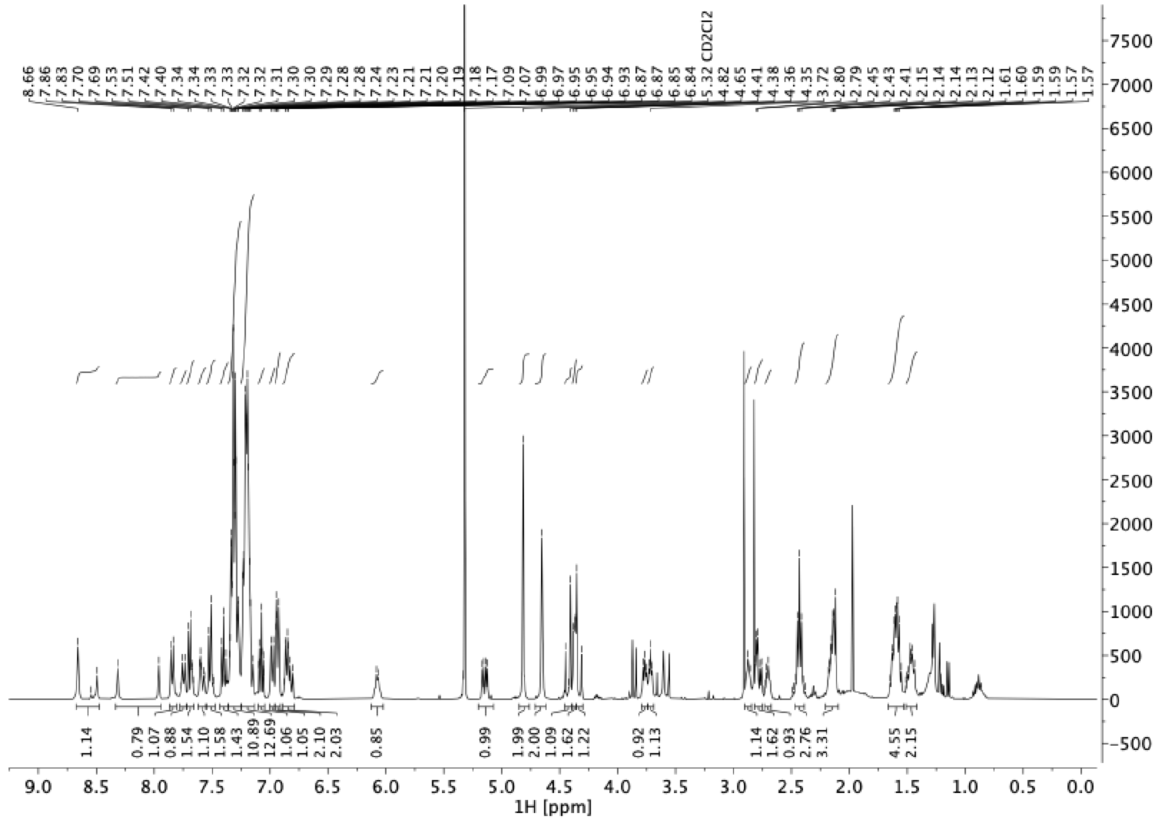


$^{31}$P-NMR of compound **S1**

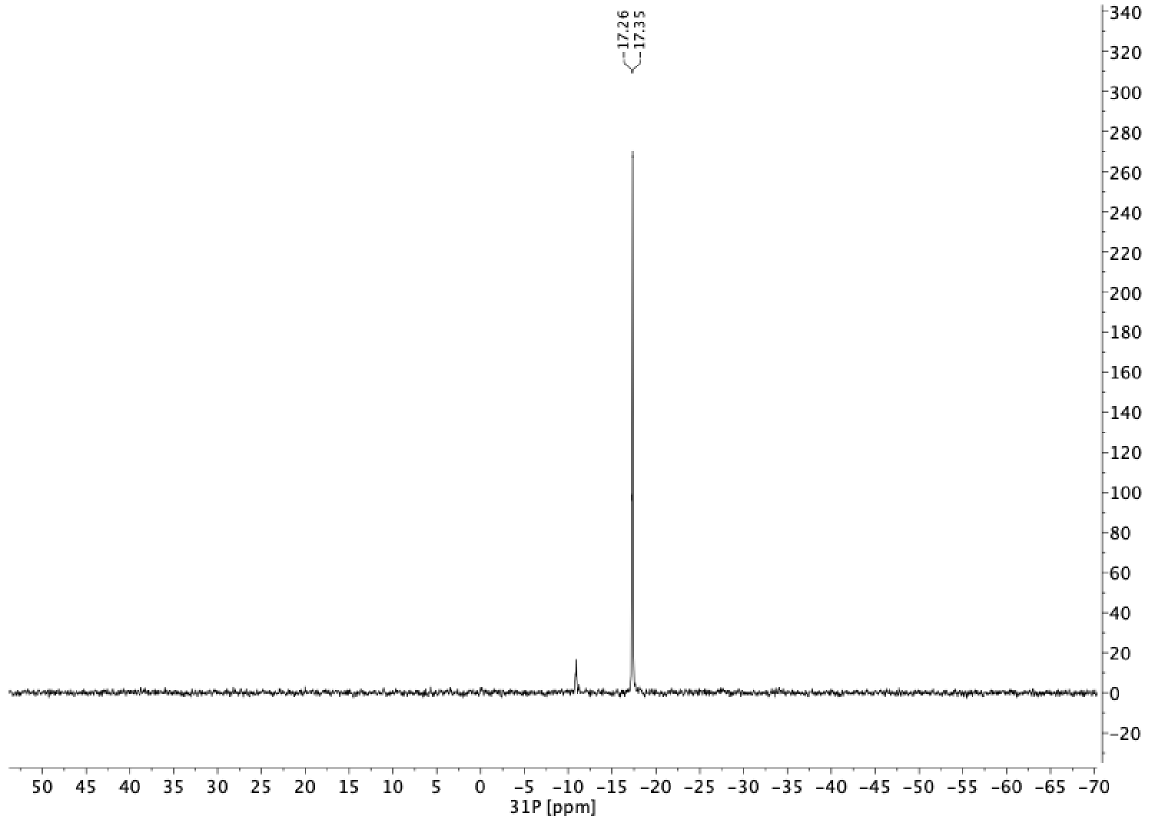

$^{13}$C-NMR of compound **S1**

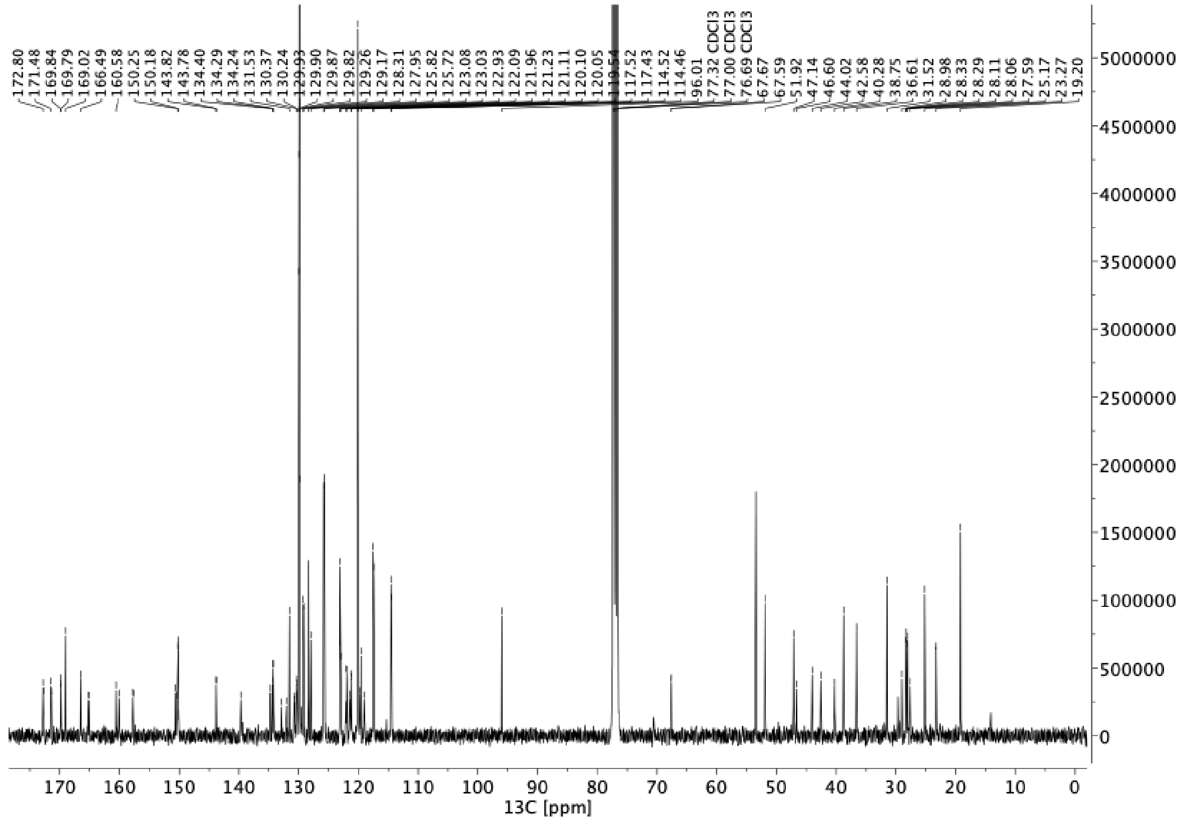

**Supporting references**


[1] J. Gräb, A. Berg, L. Blechschmidt, B. Klüver, S. Rubner, D. Y. Fu, J. Meiler, M. Gräber, T. Berg "The STAT5b Linker Domain Mediates the Selectivity of Catechol Bisphosphates for STAT5b over STAT5a", *ACS Chem. Biol.* **2019**, *14*, 796-805.

[2] J. Schust, B. Sperl, A. Hollis, T. U. Mayer, T. Berg "Stattic: a small-molecule inhibitor of STAT3 activation and dimerization", *Chem. Biol.* **2006**, *13*, 1235-1242.

[3] J. Schust, T. Berg "A high-throughput fluorescence polarization assay for signal transducer and activator of transcription 3", *Anal. Biochem.* **2004**, *330*, 114-118.

[4] M. Gräber, W. Janczyk, B. Sperl, N. Elumalai, C. Kozany, F. Hausch, T. A. Holak, T. Berg "Selective targeting of disease-relevant protein binding domains by O-phosphorylated natural product derivatives", *ACS Chem. Biol.* **2011**, *6*, 1008-1014.

[5] N. Elumalai, A. Berg, K. Natarajan, A. Scharow, T. Berg "Nanomolar Inhibitors of the Transcription Factor STAT5b with High Selectivity over STAT5a", *Angew. Chem. Int. Ed.* **2015**, *54*, 4758-4763.

[6] J. Müller, J. Schust, T. Berg "A high-throughput assay for signal transducer and activator of transcription 5b based on fluorescence polarization", *Anal. Biochem.* **2008**, *375*, 249-254.

[7] T. Münzel, A. Berg, C. Protzel, S. Schäfer, A. Jensen-Feinhals, T. Berg "Stafib-2-CR: an Improved Nanomolar and Selective Inhibitor of the Transcription Factor STAT5b Developed by Conformational Restriction of Stafib-2", *Chem. Eur. J.* **2025**, e02809.

[8] S. Keller, C. Vargas, H. Zhao, G. Piszczek, C. A. Brautigam, P. Schuck "High-precision isothermal titration calorimetry with automated peak-shape analysis", *Anal. Chem.* **2012**, *84*, 5066-5073.

[9] T. H. Scheuermann, C. A. Brautigam "High-precision, automated integration of multiple isothermal titration calorimetric thermograms: new features of NITPIC", *Methods* **2015**, *76*, 87-98.

[10] J. C. D. Houtman, P. H. Brown, B. Bowden, H. Yamaguchi, E. Appella, L. E. Samelson, P. Schuck "Studying multisite binary and ternary protein interactions by global analysis of isothermal titration calorimetry data in SEDPHAT: Application to adaptor protein complexes in cell signaling", *Protein Sci.* **2007**, *16*, 30-42.

[11] C. A. Brautigam, in *Methods Enzymol., Vol. 562* (Ed.: J. L. Cole), Academic Press, **2015**, pp. 109-133.

[12] C. A. Schneider, W. S. Rasband, K. W. Eliceiri "NIH Image to ImageJ: 25 years of image analysis", *Nat. Methods* **2012**, *9*, 671-675.

[13] M. Mendez Perez, N. Rackelmann, L. Bialy, S. Guessregen, M. Will, T. Boehme, A. Villar Garea, M. H. Korn, M. Besenius, J. Riedel, U. Werner, M. Podeschwa "Insulin conjugates", *US20200181223A1* **2020**.

[14] H. Zhou, L. Bai, R. Xu, Y. Zhao, J. Chen, D. McEachern, K. Chinnaswamy, B. Wen, L. Dai, P. Kumar, C. Y. Yang, Z. Liu, M. Wang, L. Liu, J. L. Meagher, H. Yi, D. Sun, J. A. Stuckey, S. Wang "Structure-Based Discovery of SD-36 as a Potent, Selective, and Efficacious PROTAC Degrader of STAT3 Protein", *J. Med. Chem.* **2019**, *62*, 11280-11300.

[15] Q. Liu, F. Huang, X. Yuan, K. Wang, Y. Zou, J. Shen, Y. Xu "Structure-Guided Discovery of Novel, Potent, and Orally Bioavailable Inhibitors of Lipoprotein-Associated Phospholipase A2", *J. Med. Chem.* **2017**, *60*, 10231-10244.

[16] N. Elumalai, A. Berg, S. Rubner, L. Blechschmidt, C. Song, K. Natarajan, J. Matysik, T. Berg "Rational development of Stafib-2: a selective, nanomolar inhibitor of the transcription factor STAT5b", *Sci. Rep.* **2017**, *7*, 819.

[17] S. Caddick, D. B. Judd, A. K. d. K. Lewis, M. T. Reich, M. R. V. Williams "A generic approach for the catalytic reduction of nitriles", *Tetrahedron* **2003**, *59*, 5417-5423.

[18] H. Zheng, Y. Dong, L. Li, B. Sun, L. Liu, H. Yuan, H. Lou "Novel Benzo[a]quinolizidine Analogs Induce Cancer Cell Death through Paraptosis and Apoptosis", *J. Med. Chem.* **2016**, *59*, 5063-5076.
